# Transition Metal-Free Regioselective Phosphonation of Pyridines: Scope and Mechanism

**DOI:** 10.1021/acsorginorgau.2c00055

**Published:** 2023-02-02

**Authors:** Valentin Quint, Thi Hong Van Nguyen, Gary Mathieu, Saloua Chelli, Martin Breugst, Jean-François Lohier, Annie-Claude Gaumont, Sami Lakhdar

**Affiliations:** † Normandie University, LCMT, ENSICAEN, UNICAEN, CNRS, 6, Boulevard Maréchal Juin, Caen 14000-France; ‡ CNRS, Université Paul Sabatier, Laboratoire Hétérochimie Fondamentale et Appliquée (LHFA, UMR5069), 118 Route de Narbonne, 31062 Cedex 09 Toulouse, France; § Institut für Chemie, Technische Universität Chemnitz, 09111 Chemnitz, Germany

**Keywords:** C−H functionalization, DFT calculations, mechanisms, phosphonation, pyridines, reactivity

## Abstract

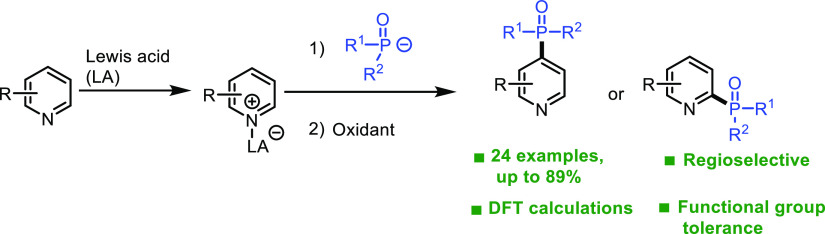

Regioselective phosphonation of pyridines is an interesting transformation in synthesis and medicinal chemistry. We report herein a metal-free approach enabling access to various 4-phosphonated pyridines. The method consists of simply activating the pyridine ring with a Lewis acid (BF_3_·OEt_2_) to facilitate the nucleophilic addition of a phosphine oxide anion. The formed sigma complex is subsequently oxidized with an organic oxidant (chloranil) to yield the desired adducts in good to excellent yields. We furthermore showed that access to C2-phosphoinated pyridines can be achieved in certain cases with strong Lewis basic phosphorus nucleophiles or with strong Lewis acidic pyridines. Both experimental and computational mechanistic investigations were undertaken and allowed us to understand the factors controlling the reactivity and selectivity of this reaction.

## Introduction

Pyridines are key motifs in agrochemicals, functional materials, transition metal catalysis, and organocatalysis.^1−4^ Thus, the development of practical methods for the selective functionalization of these heterocycles remains an important challenge for both academia and pharmaceutical industry. Apart from the selective activation of C–H bonds of pyridine to forge C–C, C–N, and C–O bonds, the introduction of C–P bonds is of high importance as its enables access to phosphonates, phosphine oxides, and phosphines, which are highly important scaffolds in materials science, biochemistry, and catalysis.^5^ More importantly, the development of site-selective phosphonation has attracted much attention during the last decades, given the capability of those approaches to furnish organophosphorus compounds in straightforward and step-economical fashions.^6,7^

For instance, the addition of triphenylphosphine to a pyridinium ion, followed by deprotonation, has allowed the group of McNally to synthesize a large variety of phosphonium ions that were converted to highly useful C4-functionalized pyridines.^8−10^

**Scheme 1:**
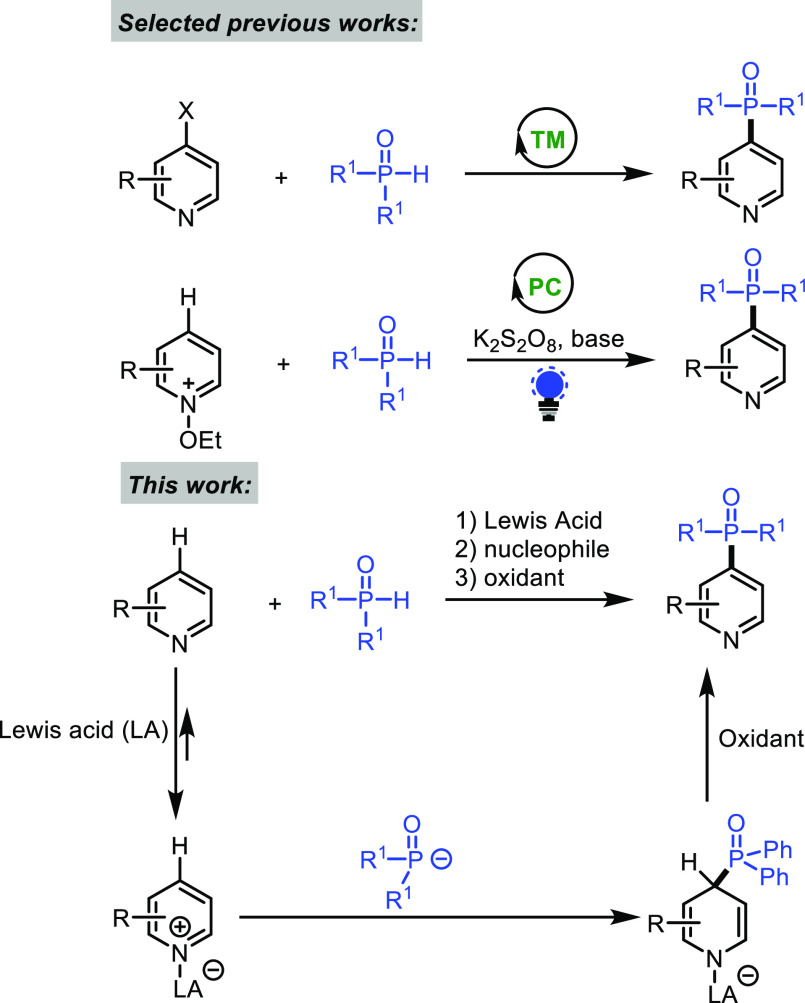
Different Approaches for the Site-selective Phosphonation of pyridines. TM: Transition Metal, PC: Photocatalyst

Moreover, a plethora of interesting approaches, mainly using transition metal catalysis, allowing direct access to phosphosphonated pyridines from readily available starting materials have emerged. Recently, the field has gained much interest with the renaissance of the field of photoredox catalysis.^11−16^ For instance, the Hong group has elegantly achieved the C4-phosphonation of pyridines by combing *N*-ethoxypyridinium salts with secondary phosphine oxides in the presence of an oxidant (K_2_S_2_O_8_) and an organophotocatalyst under blue light irradiation (Scheme 1).^17^

In contrast to transition metal and photocatalytic methods outlined above, the use of polar chemistry to achieve selective C-4 phosphonation has not been extensively investigated.^18^ As part of our interest to develop mechanistically driven approaches for the formation of C–P,^19−23^ we hypothesized that the use of oxidative Chichibabin-type reaction, that is, nucleophilic addition of a phosphine oxide anion to an activated pyridine followed by oxidative aromatization, would provide a straightforward access to C-4 phosphonated pyridines through the mechanism outlined in Scheme 1.^24^

## Results and Discussion

To test our hypothesis, we investigated the reaction of 3-cyanopyridine (**1a**) with diphenyl phosphine oxide (**2a**) in the presence of a Lewis acid, a base, and an oxidant. To ensure full activation of pyridine and to avoid the complexation of the nucleophile with the Lewis acid, 1.1 equiv of the latter was added to the pyridine in THF at −78 °C. To this mixture was added diphenylphosphine oxide anion, which was generated in situ by adding a base to diphenylphosphine oxide (**2a**) in THF. Finally, the oxidant was added to obtain the desired phosphonated pyridine (**3a**)

As shown in Table 1, we first evaluated the effect of the base on the reaction by taking BF_3_·OEt_2_ as the Lewis acid and chloranil as the oxidant. Unsurprisingly, no reaction took place when the relatively weak Brønsted bases such as NaHCO_3_ (entry 1) and NaOH (entry 2) were employed. However, 79% of **3a** was isolated by column chromatography and characterized by X-ray diffraction.^25^ When *t*BuOK was used as a base, a good conversion was observed (entry 3). Unsurprisingly, the reaction did not proceed in the absence of a base (entry 4). Keeping *t*BuOK as the base of choice, we next examined the effect of the Lewis acid on the reaction. While no reaction occurred in the absence of BF_3_·OEt_2_ (entry 5), modest or low yields were obtained when BCl_3_ or 0.2 equiv of BF_3_·OEt_2_ was employed, respectively (entries 6 and 7). The nature of the oxidant turned out to be a key factor for the feasibility of the reaction as mild oxidants such as air, S_8_, O_2_, and I_2_ failed to drive the reaction (entries 8–11). Although 54% of the isolated yield of **3a** was obtained when DDQ was used as the oxidant (entry 12), it remained less efficient as chloranil. Other solvents like acetonitrile and diethylether (entries 13 and 14) were tested and gave lower yields than that obtained with THF. Finally, increasing the temperature to −40 °C slightly diminished the yield of the reaction. Taken together, the best reaction conditions used to explore the scope were those depicted in entry 3 of Table 1.

**Table 1:**
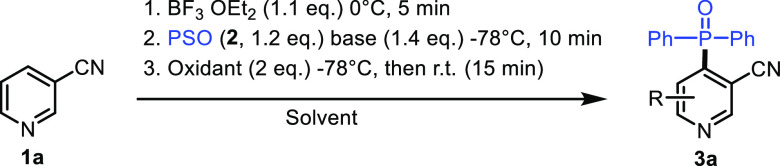
Optimization of the C4-Phosphonation of 3-Cyanopyridine^a^

entry	base	oxidant	solvent	**3a**, yield [%]^b^
1	NaHCO_3_	chloranil	THF	
2	NaOH	chloranil	THF	
3	*t*BuOK	chloranil	THF	79
4		chloranil	THF	
5	*t*BuOK^c^	chloranil	THF	
6	*t*BuOK^d^	chloranil	THF	52
7	*t*BuOK^e^	chloranil	THF	17
8	*t*BuOK	air	THF	
9	*t*BuOK	S_8_	THF	
10	*t*BuOK	O_2_	THF	
11	*t*BuOK	I_2_	THF	
12	*t*BuOK	DDQ	THF	54
13	*t*BuOK	chloranil	ACN	64
14	*t*BuOK	chloranil	Et_2_O	62
15	*t*BuOK^f^	chloranil	THF	71

a Reaction conditions: 3-cyanopyridine **1a** (1 mmol, 1 equiv), BF_3_·OEt_2_ (1.1 mmol, 1.1 equiv), diphenylphosphine oxide **2a** (1.2 mmol, 1.2 equiv), *t*BuOK (1.4 mmol, 1.4 equiv), chloranil (2 mmol), solvent (2 mL) at −78 °C, 10 min.

b Isolated yield.

c In the absence of BF_3_·OEt_2_.

d BCl_3_ instead of BF_3_·OEt_2_.

e 0.2 equiv of BF_3_·OEt_2_.

f *T* = −40 °C.

**Figure 1:**
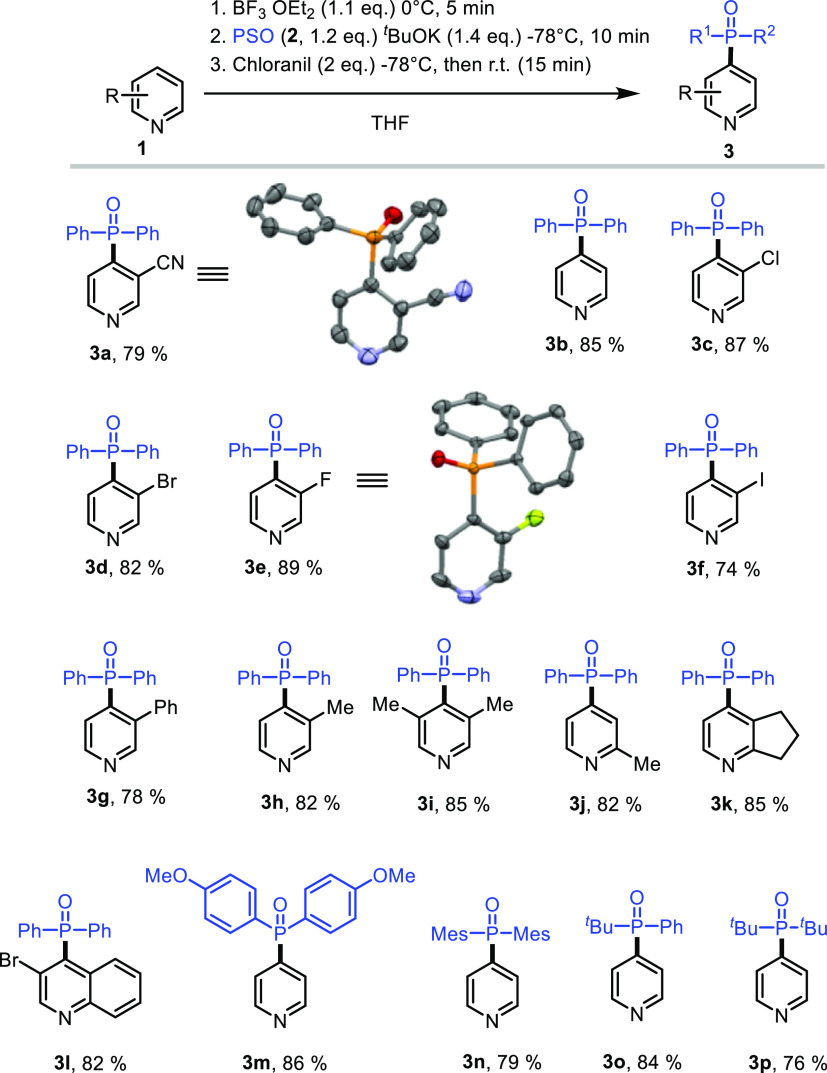
Substrate scope for metal-free phosphonation.

With the optimized reaction conditions in hand, we next explored the scope of the reaction (Figure 1). The parent pyridine gave only C-4 regioisomer **3b** in 85% yield, and products resulting from C2- or C6-phosphonations could not be detected under the reaction conditions. The same regioselectivity was observed with different pyridines bearing either electron-withdrawing (**3c–3g**) or -donating (**3h**) groups at the C3-position. Importantly, halogens [chloro (**3c**), bromo (**3d**), fluoro (**3e**),^26^ and idodo (**3f**)] groups were all tolerated under our reaction conditions. The reaction proceeds smoothly with disubstituted pyridine, furnishing the C4-adduct (**3i**) in good yield (85%). Moreover, the reaction works well with 2-methylpyridine (**3h**, 82%), 6,7-dihydro-5*H*-cyclopenta[*b*]pyridine (**3k**, 83%), and 3-bromoquinoline (**3l**, 82%). We further tested other phosphine oxides with diphenyl phosphine oxide (**2a**). As shown in Figure 1, diaryl phosphine oxides (**3m** and **3n**) resulted in good yields (79–86%). Finally, alkyl arylphosphine oxide and dialkyphosphine oxide were also compatible with the reaction conditions and gave the desired adducts **3o** and **3p** in good yields.

**Figure 2:**
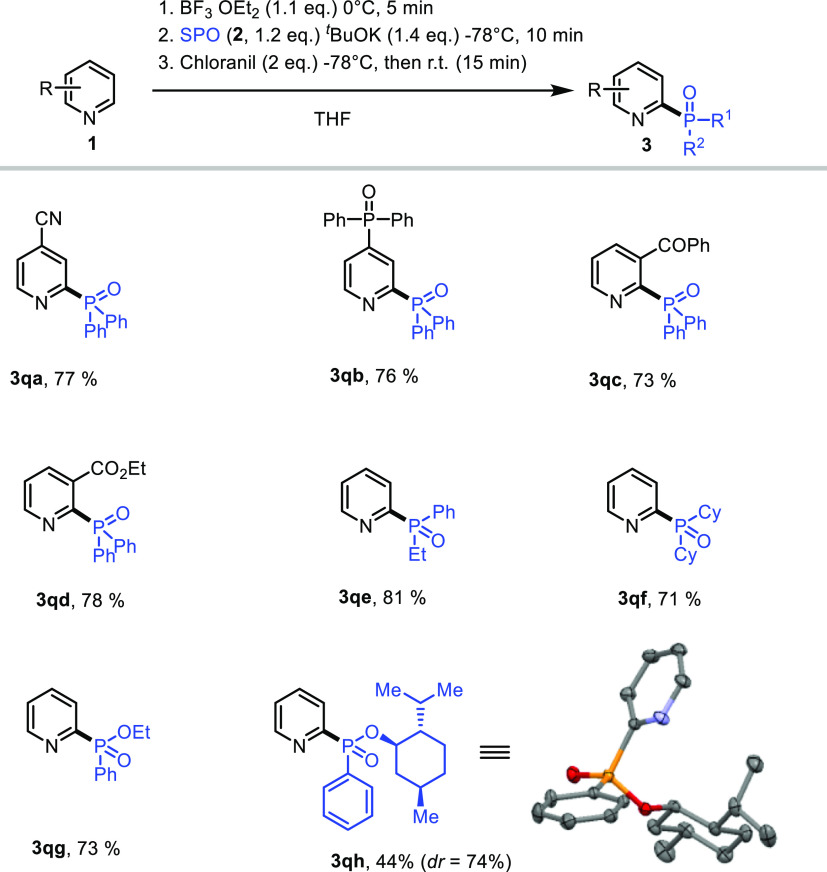
C2-regioselectivity of the phosphonation of pyridines.

**Figure 3:**
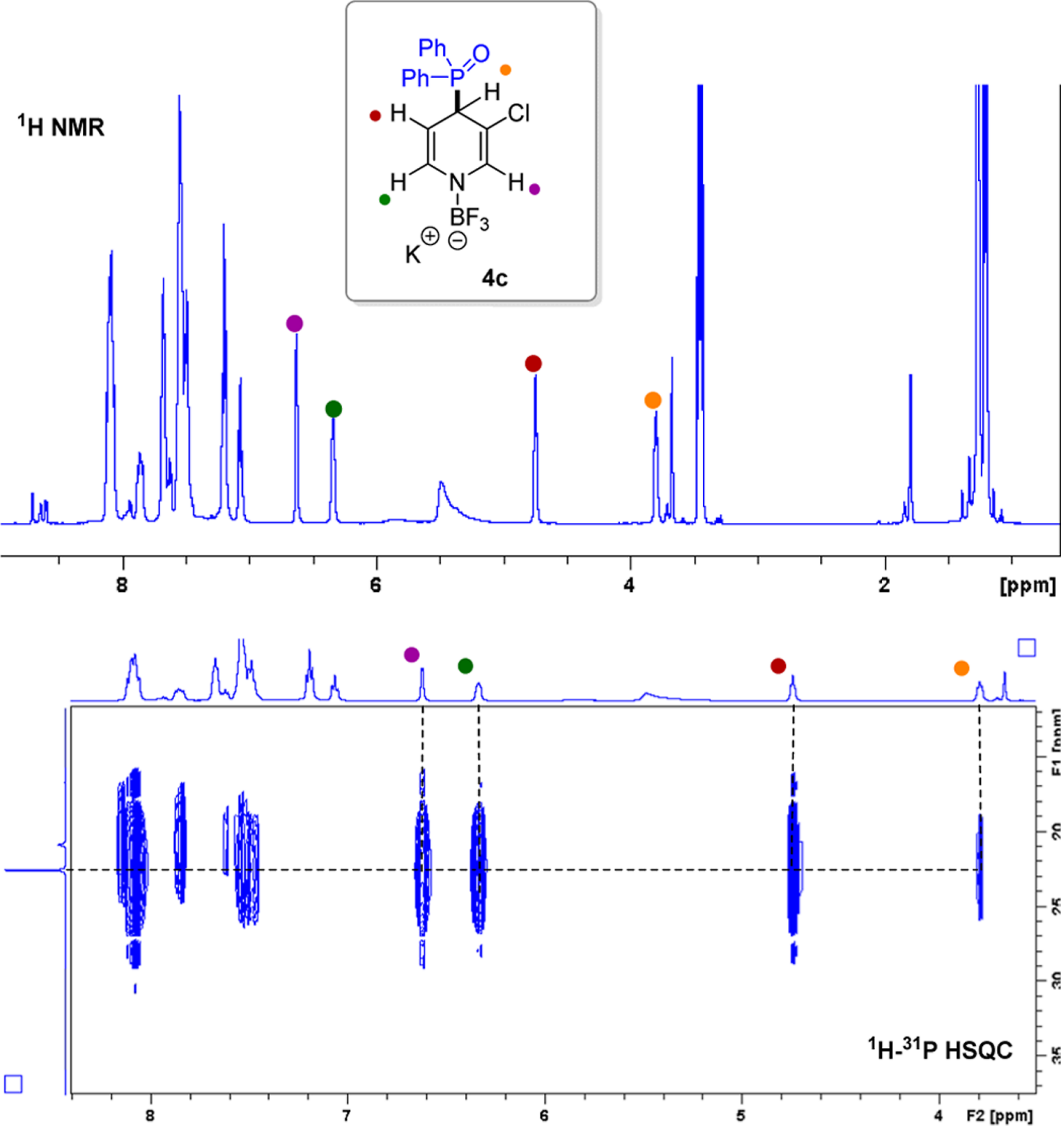
^1^H–^31^P HSQC spectrum of intermediate **4c**.

Evidently, the C2-regioisomer could be obtained when the C4-position of the pyridine is occupied with cyano (**3qa**) or diphenylphosphine oxide groups (**3qb**). Interestingly, pyridine-bearing carbonyls at the C3 position led to the exclusive formation of C2-phosphonated pyridines **3qc** and **3qd**. However, unlike *tert*-butyl(phenyl)- and di-*tert*-butyl-phosphine oxide that gave only C4-adducts under the optimized reaction conditions (Figure 1, **3o** and **3p**), the C2-regioisomers were obtained when the reaction was carried out with ethyl(phenyl)phosphine oxide (**3qe**) and dicyclohexylphosphine oxide (**3qf**). The same C2-regioselectivity was observed with phosphinate (**3qg**) in good yield (73%). Importantly, highly diasteroselective C2-phosphonation (*dr* = 74%) could be achieved with a chiral phosphinate, leading to **3qh** in fair yield (55%) (Figure 2). Crystallization of this mixture leads to the isolation of a pure diastereomer in 44% yield, which was characterized by X-ray diffraction.^27^

**Scheme 2:**
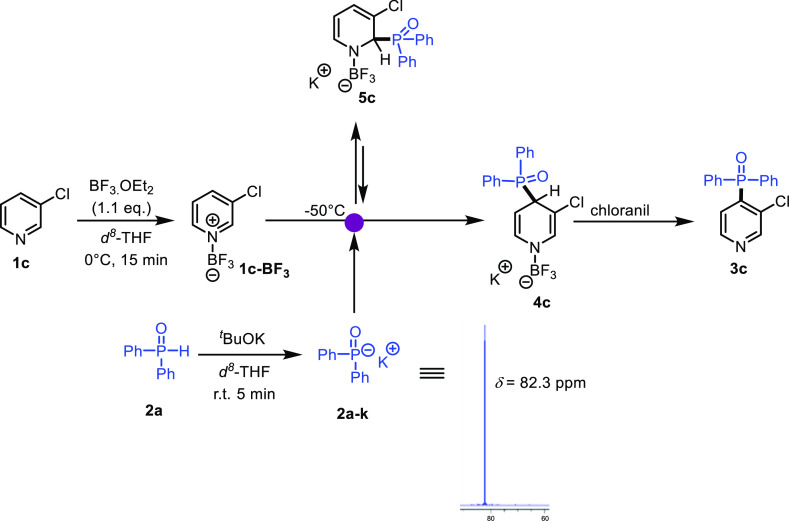
Investigation of the Reaction Mechanism of the Phosphonation of Pyridine **1c** by ^31^P NMR Spectroscopy

Having examined the scope of the phosphonation reaction, we turned our attention to the mechanism of this metal-free transformation. In accordance with previous work by Moore,^28^ the addition of BF_3_·OEt_2_ to pyridine **1c** leads to a fast and quantitative formation of the expected Lewis base–Lewis base adduct (**1c-BF**_**3**_) as attested by ^1^H and ^11^B NMR spectroscopy experiments. When 1 equiv of *t*BuOK was added to diphenyl phosphine oxide (**2a**), a new signal relaxing at δ_P_ = 82.3 ppm corresponding to anion **2a–k** was observed in deuterated THF (Scheme 2).

The addition of this anion to the complex **1c-BF**_**3**_ results in the immediate appearance of a new signal at 22.8 ppm as attested by ^31^P NMR. To elucidate the structure of the new species, 2D heteronuclear single quantum correlation (^1^H–^31^P HSQC) was performed in *d*^8^-THF at −50 °C. As shown in Figure 3, the phosphorus signal at 22.8 ppm correlates with all dihydropyridine protons (Figure 3). The ^1^H NMR spectrum shows coupling of these protons with the phosphorus nucleus. These spectroscopic results suggest the formation of sigma complex **4c**.^29^

The spectroscopic information outlined above strongly support the mechanism depicted in Scheme 2, where the reaction starts with the formation of adduct **1-BF**_**3**_. The regioselectivity (C4 vs C2) attack depends on the Lewis basicity of the phosphine oxide anion.^30^ Indeed, strong Lewis bases such as ethyl(phenyl)phosphine oxide- (**1qe**), di-cyclohexylphosphine oxide-anion (**1qf**), and ethylphenylphosphinate-anions react irreversibly at the C-2 position of **1-BF**_**3**_. By contrast, weaker Lewis bases such as diarylphosphine oxide anions react reversibly at the C-2 position due to steric clash between substituent of the nucleophile and fluorine atoms of **1-BF**_**3**_, which lead to the exclusive formation of the thermodynamic adducts (C-4) (Scheme 2). The high regioselectivity observed in the cases of **3qc** and **3qd** might be explained by the high Lewis acidity of the activated pyridine **1-BF**_**3**_.

**Figure 4:**
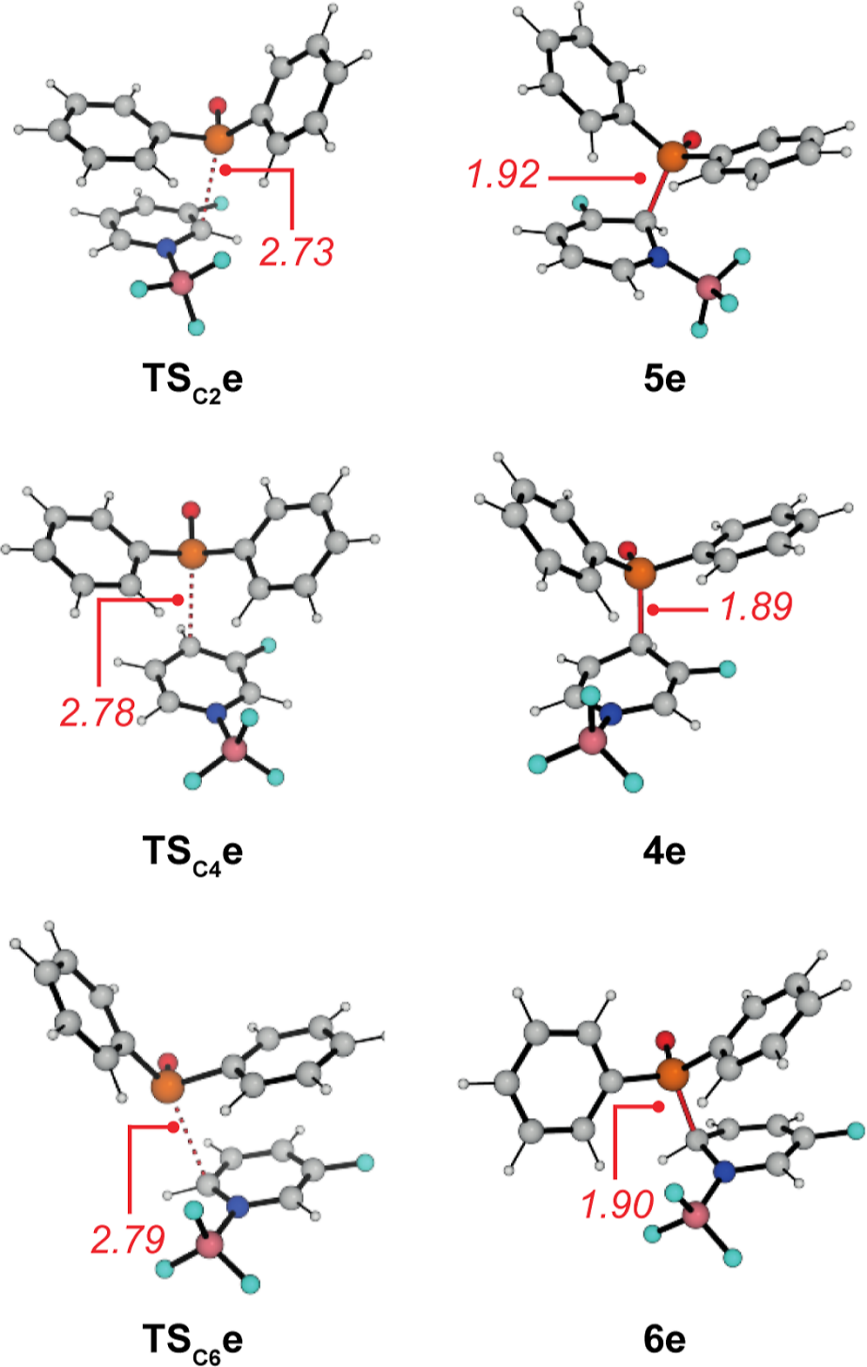
Calculated transition states and σ-complexed with selected bond length (in Å) for the phosphonation of **1e-BF**_**3**_.

To further support the mechanistic proposal, we performed a computational investigation at the DLPNO-CCSD(T)/def2-TZVPPD/SMD(THF)//ωB97X-D/6-311+G(d,p) for selected **1-BF**_**3**_ adducts. In line with the experimental findings discussed above, our calculations also predict a strong interaction energy between the free pyridine and BF_3_ (−79 < Δ*G* < −67 kJ mol^–1^). We then focused on the formation of the C–P bond as the key step of this transformation and considered a potential nucleophilic attack at C2, C4, and C6 of the activated pyridine (Table 2). In line with the high reactivity of both reaction partners, the computed barriers for this step are all relatively low and indicate rapid reactions even at lower temperatures. In all cases, phosphonation at C4 leads to the thermodynamically most stable sigma complexes **4**, which are substantially more stable than the isomeric structures **5** and **6**. In contrast, the kinetic preference is not as clear throughout this series. In line with the increasing electron deficiency, the activation free energies generally decrease within the series **1a** → **1g** → **1h**. However, substantially smaller differences were calculated for the activation free energies of **TS**_**C2**_, **TS**_**C4**_, and **TS**_**C6**_ compared to the sigma complexes. Selected structures for these transition states are shown in Figure 4 for the reaction of the 3-fluorinated pyridine complex **1e-BF**_**3**_. In all transition-state structures, the C–P bonds are very long (2.73–2.79 Å) indicating very early transition states. Even in the sigma complexes, these bonds are still slightly elongated (1.89–1.92 Å compared to 1.82–1.83 Å for the other C_Ar_–P bonds). Interestingly, the most electron-deficient substrates studied computationally (**1qc**) now also features a substantial kinetic preference for an attack at either C2 or C6. The computational investigations predict a preferential kinetic attack at the C6 position, which is experimentally not observed (see Figure 2). Given the low calculated barriers and the bimolecular character of this reaction step, a substantial part of the activation energy stems from entropic contributions, which can be difficult to calculate accurately^31,32^ and could be responsible for this deviation. In general, the computational investigations further support the hypothesis of thermodynamic control of the phosphonation reaction.

**Table 2:**
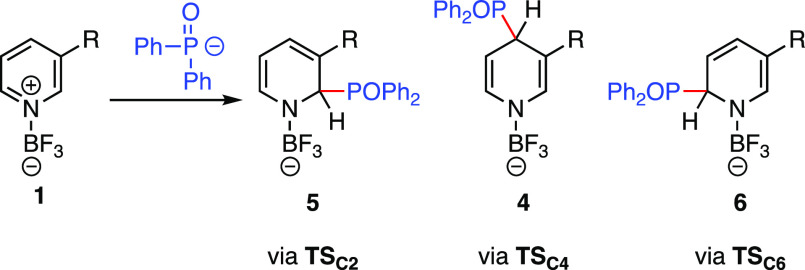
Computational Results for the Regioselective Phosphonation^a^

R	TS_C2_	5	TS_C4_	4	TS_C6_	6
H (**1b**)	+59	–4	+54	–31		
3-F (**1e**)	+46	–20	+50	–43	+42	–6
3-Ph (**1g**)	+45	–18	+40	–36	+49	–15
3-COPh (**1qc**)	+28	–55	+37	–83	+23	–59

a DLPNO-CCSD(T)/def2-TZVPPD/SMD(THF)//ωB97X-D/6-311+G(d,p), kJ mol^–1^.

In conclusion, we have developed a metal-free BF_3_·OEt_2_-mediated phosphonation of various pyridines. The reaction is practically simple, highly yielding, and completely C4-regioselective. Mechanistic investigations have allowed the characterization of the sigma complex, formed from the nucleophilic addition of the phosphine oxide anion to activated pyridines (1-BF_3_), as a key intermediate in this transformation. Interestingly, the C2-regioselectivity observed with dialkyl and alkylaryl phopshine oxides was attributed to the high Lewis basicity of the corresponding anions. The use of this completely regioselective approach for the site-selective functionalization of pyridines with other nucleophiles is being studied in our laboratories and will be reported in due course.

## Data Availability

The data underlying this study are available in the published article and its online Supporting Information.

## Supporting Information Available

Reaction optimization studies, synthetic procedures, and characterization data for the phosphonation reaction, spectroscopic data for new compounds, and copies of NMR spectra (PDF)

## Dedication

This article is dedicated to the memory of Prof. Mohamed Baker Rammah (University of Monastir) (1947–2022).
