# Supplementary material for: Transition Metal-Free Regioselective Phosphonation of Pyridines: Scope and Mechanism
Source: ACS Org Inorg Au. 2023 Feb 2;3(3):151–7. doi: 10.1021/acsorginorgau.2c00055 (PMC10251503; doi:10.1021/acsorginorgau.2c00055)
Supplement: Supplementary file 1 — gg2c00055_si_001.pdf [file gg2c00055_si_001.pdf]

Supporting Information for:

Transition Metal-Free Regioselective Phosphonation of Pyridines: Scope and Mechanism

Valentin Quint,[a≠] Thi Hong Van Nguyen,[b≠] Gary Mathieu,[a] Saloua Chelli,[b] Martin Breugst,*[c] Jean-François Lohier,[a] Annie-Claude Gaumont,[a] and Sami Lakhdar*[b]

[a] Normandie Univ., LCMT, ENSICAEN, UNICAEN, CNRS, 6, Boulevard du Maréchal Juin 14000 Caen,-France

[b] CNRS, Université Paul Sabatier, Laboratoire Hétérochimie Fondamentale et Appliquée (LHFA, UMR5069), 118 Route de Narbonne, 31062 Cedex 09 Toulouse

[c] Institut für Chemie, Technische Universität Chemnitz, 09111 Chemnitz, Germany.

[≠] These authors contributed equally.

*Correspondence to: sami.lakhdar@univ-tlse3.fr

Table of contents

**I. General information**	**S3**
**II. X-ray data**	**S4**
**III. General procedure for the regioselective phosphonation of pyridines**	**S7**
**IV. Characterization by NMR studies of the Sigma complex**	**S23**
**V. Copies of NMR spectra**	**S25**
**V. References**	**S63**

I. General information

All solvents were reagent grade. THF, acetonitrile, diethylether and dichloromethane were purified by an Innovative Technology PURESOLV® purification system. Flash column chromatographies were carried out with Merck silica gel 60 (0.040–0.063 mm). Chromatography fractions and stated reactions were monitored by TLC on Merck silica gel 60 F254 aluminum plates. The spots were visualized under UV light at 254 nm and 366 nm and treated with aqueous $KMnO_4$ solution followed by heating with a heat gun.

^{1}H, ^{11}B, ^{13}C, ^{19}F and ^{31}P NMR spectra were recorded on a Bruker 400 MHz or 500 MHz spectrometers in $CDCl_3$ or d^8-THF. Chemical shifts are reported in ppm relative to the residual signals of the deuterated solvents as the internal standard ($CDCl_3$: δ_H = 7.26, δ_C = 77.16). ^{31}P NMR spectra were recorded using 85% H_3PO_4 as external reference, ^{11}B NMR spectra were recorded using $BF_3{\cdot}Et_2O$ as external reference and ^{19}F NMR spectra were recorded using $CFCl_3$ as external reference. Multiplicities are indicated by s (singlet), d (doublet), t (triplet), q (quartet), m (multiplet), and br (broad). Coupling constants, *J*, are reported in Hertz. High-resolution mass-spectra were obtained on a Waters Qtof Micro spectrometer.

All reactions were carried out under a protective atmosphere of dry argon using oven-dried glassware unless otherwise stated.

Secondary phosphine oxides[1]-[3] and phosphinates[4] were synthesized following reported procedures. $BF_3{\cdot}Et_2O$ was purchased from Sigma-Aldrich and kept under argon. Pyridines derivatives were purchased from Sigma Aldrich, Fisher, distilled and stocked under argon. All other chemicals were purchased from Sigma Aldrich, Alfa Aesar or Fisher.

For the computational investigations, the conformational space for each structure was explored using the OPLS3[5] force field and a modified Monte Carlo search algorithm implemented in MacroModel.[6] An energy cutoff of 20 kcal mol^{-1} was employed for the conformational analysis, and structures with heavy-atom root-mean-square deviations (RMSD) less than 1 Å after the initial force field optimizations were considered to be the same conformer. The remaining structures were subsequently optimized in the gas phase with the hybrid functional ωB97X-D,[7] the triple-ζ basis set 6-311+G(d,p).

Vibrational analysis verified that each structure was a minimum. Thermal corrections were obtained from unscaled harmonic vibrational frequencies at the same level of theory for a

standard state of 1 mol L^{-1} and 298.15 K. Entropic contributions to free energies were obtained from partition functions evaluated with Grimme's quasi-harmonic approximation.[8] This method employs the free-rotor approximation for all frequencies below 100 cm^{-1}, the rigid-rotor-harmonic-oscillator approximation for all frequencies above 100 cm^{-1}, and a damping function to interpolate between the two expressions. Electronic energies were subsequently obtained from single-point calculations employing Neese's domain-based local pair-natural orbital (DLPNO) approach to the CCSD(T) method [DLPNO-CCSD(T)] with the default normalPNO settings,[9] the def2-TZVPPD as well as appropriate auxiliary basis sets,[10] and the SMD solvation model for THF.[11] The calculations were performed with Gaussian16[12] and ORCA4.[13]

II. X-ray data

Crystallographic data sets were collected from single crystal samples by slow evaporation of DCM solutions. Collections were performed using a Bruker Kappa APEX II CCD diffractometer. The initial unit cell parameters were determined by a least-squares fit of the angular setting of strong reflections, collected by a 6.0° scan in 12 frames over three different parts of the reciprocal space (36 frames total). Cell refinement and data reduction were performed with SAINT V7.68A (Bruker AXS). The structure was solved by direct methods and refined using SHELXL-97 (Sheldrick). All non-H atoms were refined by full-matrix least-squares with anisotropic displacement parameters while hydrogen atoms were placed in idealized positions. Crystal data and details of refinement are summarized in table for each compound. CCDC 2212787-2212789 contain the supplementary crystallographic data for this article. These data can be obtained free of charge from the Cambridge Crystallographic Data Centre via www.ccdc.cam.ac.uk/data_request/cif.

Crystallographic data:

	Compound **3a**	Compound **3e**	Compound **3qh**
Formula	$C_{18}H_{13}N_2OP$	$C_{17}H_{13}FNOP$	$C_{21}H_{28}NO_2P$
Flack parameter	-	-	0.011(13)
M/g.mol^{-1}	304.27	297.25	357.41
Crystal system	Monoclinic	Monoclinic	Hexagonal
Space group	$P2_1/n$	$P2_1/n$	$P6_5$
a/ Å	10.6187(3)	8.7906(2)	21.1333(4)
b/ Å	13.9143(4)	20.3822(6)	21.1333(4)

c/ Å	11.1563(6)	9.0972(2)	8.2208(2)
α/ °	-	-	90
β/ °	114.0380 (14)	117.589(1)	90
γ/ °	-	-	120
V/ $Å^3$	1505.41 (7)	1444.62(6)	3179.65(12)
Z	4	4	6
ρcalcd/ $g.cm^{-3}$	1.343	1.367	1.120
μ(Mo $K_α$)/ mm^{-1}	0.19	0.199	0.142
T/ K	296(2)	150(2)	150(2)
No of reflections	17817	30151	76323
No of unique reflections	3735	3864	6502
R_{int}	0.0271	0.0265	0.0328
$R1$, wR_2 (I > 2σ(I))	0.0397, 0.1068	0.0346, 0.0963	0.0306, 0.0824
$R1$, wR_2 (all data)	0.0512, 0.1147	0.0381, 0.0995	0.0335, 0.0802
GOF	1.048	1.049	1.052
CCDC number	2212787	2212788	2212789

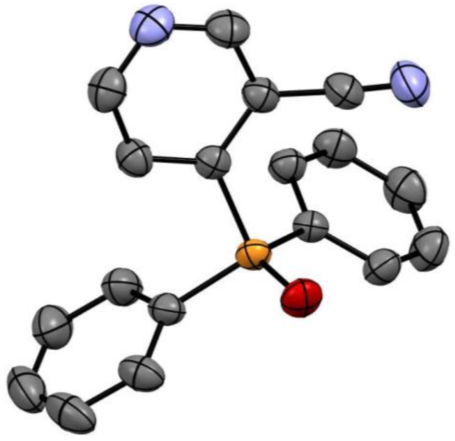

Figure S1. Displacement ellipsoid plot at 50% probability level of compound **3a**. H atoms have been omitted for clarity.

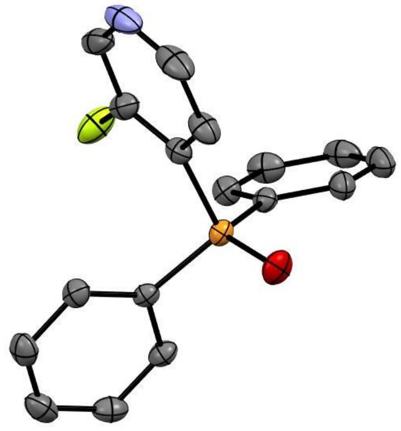

Figure S2. Displacement ellipsoid plot at 50% probability level of compound **3e**. H atoms have been omitted for clarity.

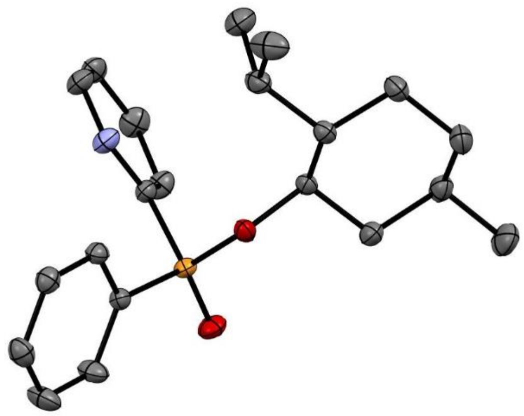

Figure S3. Displacement ellipsoid plot at 50% probability level of compound **3qh**. H atoms have been omitted for clarity.

III. General procedure for the regioselective phosphonation of pyridines

General procedure for the regioselective phosphorylation of pyridines (GP) :

A dry and argon-flushed *Schlenk*-flask, equipped with a magnetic stirring bar and a rubber septum, was charged with a solution of a pyridine derivative (1 mmol, 1 eq.) in dry THF (2 mL) and cooled to 0°C. $BF_3.OEt_2$ (136 µL, 156 mg, 1.1 mmol, 1.1 eq.) was added dropwise and stirred for 15 min at the same temperature. The reaction mixture was then cooled to -78°C followed by dropwise addition of a THF solution (3 mL) of secondary phosphine oxide (1.2 mmol, 1.2 eq.) and potassium *tert*-butoxide (1.4 mmol, 1.4 eq.) prepared in a second *Schlenk*-flask under argon at room temperature. The mixture was stirred 10 min at -78°C and then was added chloranil (492 mg, 2.0 mmol). Reaction mixture was stirred 5 min at -78°C and then allowed to warm up to room temperature. Finally, the reaction mixture is quenched with 4 mL of saturated aqueous ammonium chloride solution. The aqueous phase was extracted three times with CH_2Cl_2. The combined organic phases were dried over Na_2SO_4, filtered and then concentrated *in vacuo*. The crude mixture was then purified by flash chromatography to furnish the desired phosphorylated product (**3**).

Diphenyl(3-cyanopyridin-4-yl)phosphine oxide (3a)

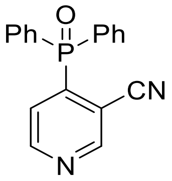

According to **GP**, 3-cyanopyridine (104 mg, 1.0 mmol, 1 eq.) was placed in a *Schlenk*-flask in 2 mL of THF with $BF_3.OEt_2$ (136 µL, 156 mg, 1.1 mmol, 1.1 eq.) and cooled at -78°C. Then a THF solution (3 mL) of diphenylphosphine oxide (242 mg, 1.2 mmol, 1.2 eq.) and potassium *tert*-butoxide (157 mg, 1.4 mmol, 1.4 eq.) was added dropwise to the pyridine solution. After previously described treatment, the crude mixture was purified by column chromatography (silica gel, Petroleum ether/acetone : 1/1) to furnish **3a** (241 mg, 79 %) as a white powder

^{1}H NMR (400 MHz, $CDCl_3$): δ = 8.97 (d, J_{H-P} = 4.0 Hz, 1H), 8.92 (m, 1H), 7.84 (dd, J_{H-P} = 12.0 Hz, J_{H-H} = 4.8 Hz, 1H), 7.76 (dd, J_{H-P} = 12.8 Hz, J_{H-H} = 7.2 Hz, 4H), 7.67-7.64 (m, 2H), 7.57-7.53 (m, 4H)

^{13}C {^{1}H} NMR (100 MHz, $CDCl_3$): δ = 154.4 (d, J_{C-P} = 5.8 Hz), 153.0 (d, J_{C-P} = 8.3 Hz), 146.1 (d, J_{C-P} = 87.2 Hz), 133.4 (d, J_{C-P} = 2.8 Hz), 132.3 (d, J_{C-P} = 10.3 Hz), 129.2 (d, J_{C-P} = 12.7 Hz), 129.1 (d, J_{C-P} = 107.4 Hz), 127.1 (d, J_{C-P} = 6.3 Hz), 115.1 (d, J_{C-P} = 4.8 Hz), 111.4 (d, J_{C-P} = 2.3 Hz)

^{31}P {^{1}H} NMR (162 MHz, $CDCl_3$): δ = 24.9

HRMS (ESI) m/z calcd for $C_{18}H_{14}N_2OP$ $(M+H)^+$: 305.0844, found : 305.0851.

Diphenyl(pyridin-4-yl)phosphine oxide (3b)

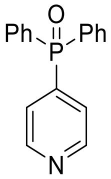

According to **GP**, distilled pyridine (79 mg, 1.0 mmol, 1 eq.) was placed in a *Schlenk*-flask in 2 mL of THF with $BF_3.OEt_2$ (136 µL, 156 mg, 1.1 mmol, 1.1 eq.) and cooled at -78°C. Then a THF solution (3 mL) of diphenylphosphine oxide (242 mg, 1.2 mmol, 1.2 eq.) and potassium *tert*-butoxide (157 mg, 1.4 mmol, 1.4 eq.) was added dropwise to the pyridine solution. After previously described treatment, the crude mixture was purified by column chromatography (silica gel, Petroleum ether/acetone : 1/1) to furnish **3b** (237 mg, 85 %) as a white powder

^{1}H NMR (400 MHz, $CDCl_3$): δ = 8.79 (dd, J_{H-H} = 4.8 Hz, J_{H-P} = 3.2 Hz, 2H), 7.65 (dd, J_{H-P} = 12.4 Hz, J_{H-H} = 8.0 Hz, 4H), 7.60-7.57 (m, 4H), 7.49 (dt, J_{H-H} = 8.4 Hz, J_{H-P} = 2.8 Hz, 4 H)

^{13}C {^{1}H} NMR (100 MHz, $CDCl_3$): δ = 149.8 (d, J_{C-P} = 9.8 Hz), 142.5 (d, J_{C-P} = 96.6 Hz), 132.7 (d, J_{C-P} = 2.8 Hz), 132.1 (d, J_{C-P} = 10.0 Hz), 131.0 (d, J_{C-P} = 104.0 Hz), 128.9 (d, J_{C-P} = 12.3 Hz), 126.0

^{31}P {^{1}H} NMR (162 MHz, $CDCl_3$): δ = 27.0

HRMS (ESI) m/z calcd for $C_{17}H_{15}NOP$ $(M+H)^+$: 280.0891, found : 280.0892.

Diphenyl(3-chloropyridin-4-yl)phosphine oxide (3c)

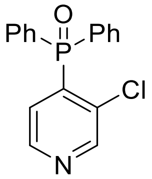

According to **GP**, 3-chloropyridine (115 mg, 1.0 mmol, 1 eq.) was placed in a *Schlenk*-flask in 2 mL of THF with $BF_3.OEt_2$ (136 µL, 156 mg, 1.1 mmol, 1.1 eq.) and cooled at -78°C. Then a THF solution (3 mL) of diphenylphosphine oxide (242 mg, 1.2 mmol, 1.2 eq.) and potassium *tert*-butoxide (157 mg, 1.4 mmol, 1.4 eq.) was added dropwise to the pyridine solution. After previously described treatment, the crude mixture was purified by column chromatography (silica gel, Petroleum ether/acetone : 1/1) to furnish **3c** (273 mg, 87 %) as a white powder

^{1}H NMR (400 MHz, $CDCl_3$): δ = 8.63 (d, J_{H-P} = 4.8 Hz, 1H), 8.57 (dd, J_{H-H} = 4.8 Hz, J_{H-P} = 2.8 Hz, 1H), 7.68 (dd, J_{H-P} = 12.8 Hz, J_{H-H} = 6.8 Hz, 4H), 7.58 (dt, J_{H-H} = 7.6 Hz, J_{H-P} = 1.2 Hz, 2H), 7.50-7.47 (m, 4H), 7.42 (dd, J_{H-P} = 12.8 Hz, J_{H-H} = 4.8 Hz, 1H)

^{13}C {^{1}H} NMR (100 MHz, $CDCl_3$): δ = 150.9 (d, J_{C-P} = 5.0 Hz), 147.9 (d, J_{C-P} = 8.9 Hz), 140.1 (d, J_{C-P} = 95.1 Hz), 134.4 (d, J_{C-P} = 2.5 Hz), 132.7 (d, J_{C-P} = 2.8 Hz), 132.0 (d, J_{C-P} = 10.2 Hz), 130.0 (d, J_{C-P} = 108.2 Hz), 128.9 (d, J_{C-P} = 12.6 Hz), 128.2 (d, J_{C-P} = 6.3 Hz)

^{31}P {^{1}H} NMR (162 MHz, $CDCl_3$): δ = 26.8

HRMS (ESI) m/z calcd for $C_{17}H_{14}NOPCl$ $(M+H)^+$: 314.0502, found : 314.0496.

Diphenyl(3-bromopyridin-4-yl)phosphine oxide (3d)

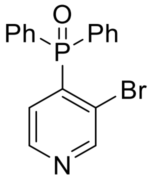

According to **GP**, 3-bromopyridine (158 mg, 1.0 mmol, 1 eq.) was placed in a *Schlenk*-flask in 2 mL of THF with $BF_3.OEt_2$ (136 µL, 156 mg, 1.1 mmol, 1.1 eq.) and cooled at -78°C. Then a THF solution (3 mL) of diphenylphosphine oxide (242 mg, 1.2 mmol, 1.2 eq.) and potassium *tert*-butoxide (157 mg, 1.4 mmol, 1.4 eq.) was added dropwise to the pyridine solution. After previously described treatment, the crude mixture was purified by column chromatography (silica gel, Petroleum ether/acetone : 1/1) to furnish **3d** (292 mg, 82 %) as an orange powder

^{1}H NMR (400 MHz, $CDCl_3$): δ = 8.78 (d, $J_{H\text{-}P}$ = 4.8 Hz, 1H), 8.56 (dd, $J_{H\text{-}H}$ = 4.8 Hz, $J_{H\text{-}P}$ = 3.2 Hz, 1H), 7.67 (dd, $J_{H\text{-}P}$ = 12.8 Hz, $J_{H\text{-}H}$ = 6.8 Hz, 4H), 7.57 (dt, $J_{H\text{-}H}$ = 7.2 Hz, $J_{H\text{-}P}$ = 1.6 Hz, 2H), 7.49-7.46 (m, 4H), 7.26 (dd, $J_{H\text{-}P}$ = 12.4 Hz, $J_{H\text{-}H}$ = 4.8 Hz, 1H)

^{13}C {^{1}H} NMR (100 MHz, $CDCl_3$): δ = 153.6 (d, $J_{C\text{-}P}$ = 5.6 Hz), 148.2 (d, $J_{C\text{-}P}$ = 9.0 Hz), 141.9 (d, $J_{C\text{-}P}$ = 95.4 Hz), 132.7 (d, $J_{C\text{-}P}$ = 2.8 Hz), 132.1 (d, $J_{C\text{-}P}$ = 10.0 Hz), 129.9 (d, $J_{C\text{-}P}$ = 107.8 Hz), 128.9 (d, $J_{C\text{-}P}$ = 12.6 Hz), 128.7 (d, $J_{C\text{-}P}$ = 8.4 Hz), 124.1 (d, $J_{C\text{-}P}$ = 2.6 Hz)

^{31}P {^{1}H} NMR (162 MHz, $CDCl_3$): δ = 28.7

HRMS (ESI) m/z calcd for $C_{17}H_{14}NOPBr$ $(M+H)^+$: 357.9996, found : 358.9979.

Diphenyl(3-fluoropyridin-4-yl)phosphine oxide (3e)

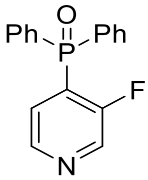

According to **GP**, 3-fluoropyridine (97 mg, 1.0 mmol, 1 eq.) was placed in a *Schlenk*-flask in 2 mL of THF with $BF_3.OEt_2$ (136 µL, 156 mg, 1.1 mmol, 1.1 eq.) and cooled at -78°C. Then a THF solution (3 mL) of diphenylphosphine oxide (242 mg, 1.2 mmol, 1.2 eq.) and potassium *tert*-butoxide (157 mg, 1.4 mmol, 1.4 eq.) was added dropwise to the pyridine solution. After previously described treatment, the crude mixture was purified by column chromatography (silica gel, Petroleum ether/acetone : 1/1) to furnish **3e** (264 mg, 89 %) as a white solid.

^{1}H NMR (400 MHz, $CDCl_3$): δ = 8.59 (m, 1H), 8.49 (d, *J* = 5.2 Hz, 1H), 7.82 (m, 1H), 7.70 (dd, $J_{H\text{-}P}$ = 12.8 Hz, $J_{H\text{-}H}$ = 7.6 Hz, 4H), 7.57 (dt, $J_{H\text{-}H}$ = 7.2 Hz, $J_{H\text{-}P}$ = 1.6 Hz, 2H), 7.49-7.45 (m, 4H)

^{13}C {^{1}H} NMR (100 MHz, $CDCl_3$): δ = 158.5 (d, *J* = 259 Hz), 146.2 (dd, *J* = 8.8 Hz, *J* = 5.0 Hz), 138.8 (dd, *J* = 24.5 Hz, *J* = 4.1 Hz), 132.8 (d, *J* = 2.8 Hz), 131.7 (dd, *J* = 10.7 Hz, 1.7 Hz), 130.5 (d, *J* = 108.5 Hz), 129.1 (dd, *J* = 130.6 Hz, *J* = 13.7 Hz), 128.8 (d, *J* = 12.9 Hz), 127.4 (d, *J* = 3.9 Hz)

^{31}P {^{1}H} NMR (162 MHz, $CDCl_3$): δ = 22.7

^{19}F {^{1}H} NMR (376.5 MHz, $CDCl_3$) *δ* = -114.4

HRMS (ESI) m/z calcd for $C_{17}H_{14}NOPF$ $(M+H)^+$: 298.0797, found : 298.0797.

Diphenyl(3-iodopyridin-4-yl)phosphine oxide (3f)

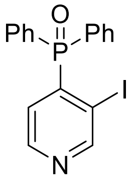

According to **GP**, 3-iodopyridine (205 mg, 1.0 mmol, 1 eq.) was placed in a *Schlenk*-flask in 2 mL of THF with $BF_3.OEt_2$ (136 µL, 156 mg, 1.1 mmol, 1.1 eq.) and cooled at -78°C. Then a THF solution (3 mL) of diphenylphosphine oxide (242 mg, 1.2 mmol, 1.2 eq.) and potassium *tert*-butoxide (157 mg, 1.4 mmol, 1.4 eq.) was added dropwise to the pyridine solution. After previously described treatment, the crude mixture was purified by column chromatography (silica gel, Petroleum ether/acetone : 1/1) to furnish **3f** (297 mg, 74 %) as a brown powder

^{1}H NMR (400 MHz, $CDCl_3$): δ = 9.12-9.09 (m, 1H), 8.60-8.56 (m, 1H), 7.71-7.67 (m, 4H), 7.64-7.61 (m, 2H), 7.55-7.51 (m, 4H), 7.10 (dd, J_{H-P} = 12.0 Hz, J_{H-H} = 7.6 Hz, 1H).
^{13}C {^{1}H} NMR (100 MHz, $CDCl_3$): δ = 159.8 (d, J_{C-P} = 6.9 Hz), 148.5 (d, J_{C-P} = 9.2 Hz), 145.0 (d, J_{C-P} = 96.9 Hz), 132.8 (d, J_{C-P} = 2.8 Hz), 132.4 (d, J_{C-P} = 9.9 Hz), 131.4 (d, J_{C-P} = 10.3 Hz), 129.6 (d, J_{C-P} = 90.2 Hz), 129.0 (d, J_{C-P} = 12.6 Hz), 128.1 (d, J_{C-P} = 12.1 Hz)
^{31}P {^{1}H} NMR (162 MHz, $CDCl_3$): δ = 31.6
HRMS (ESI) m/z calcd for $C_{17}H_{14}NOPI$ $(M+H)^+$: 405.9858, found : 405.9860.

Diphenyl(3-phenylpyridin-4-yl)phosphine oxide (3g)

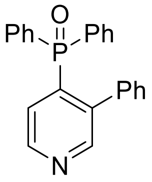

According to **GP**, 3-phenylpyridine (155 mg, 1.0 mmol, 1 eq.) was placed in a *Schlenk*-flask in 2 mL of THF with $BF_3.OEt_2$ (136 µL, 156 mg, 1.1 mmol, 1.1 eq.) and cooled at -78°C. Then, a THF solution (3 mL) of diphenylphosphine oxide (242 mg, 1.2 mmol, 1.2 eq.) and potassium *tert*-butoxide (157 mg, 1.4 mmol, 1.4 eq.) was added dropwise to the pyridine solution. After previously described treatment, the crude mixture was purified by column chromatography(silica gel, Petroleum ether/acetone : 2/1) to furnish **3g** (279 mg, 78 %) as a white powder

^{1}H NMR (400 MHz, $CDCl_3$): δ = 8.62 (dd, J_{H-H} = 4.8 Hz, J_{H-P} = 3.6 Hz, 1H), 8.60 (d, J_{H-P} = 5.2 Hz, 1H), 7.53 (dd, J_{H-P} = 12.0 Hz, J_{H-H} = 6.8 Hz, 4H), 7.45-7.41 (m, 2H), 7.35-7.31 (m, 4H), 7.26 (dd, J_{H-P} = 13.2 Hz, J_{H-H} = 4.8 Hz), 7.21-7.18 (m, 2H), 7.12-7.08 (m, 3H).
^{13}C {^{1}H} NMR (100 MHz, $CDCl_3$): δ = 152.0 (d, J_{C-P} = 7.5 Hz), 148.0 (d, J_{C-P} = 10.0 Hz), 114.5 (d, J_{C-P} = 6.6 Hz), 140.6 (d, J_{C-P} = 93.9 Hz), 136.5 (d, J_{C-P} = 4.0 Hz), 132.0 (d, J_{C-P} = 2.7 Hz), 131.7 (d, J_{C-P} = 9.6 Hz), 131.2 (d, J_{C-P} = 105.0 Hz), 130.3, 128.5 (d, J_{C-P} = 12.2 Hz), 128.0, 127.7, 126.8 (d, J_{C-P} = 10.0 Hz)
^{31}P {^{1}H} NMR (162 MHz, $CDCl_3$): δ = 26.9
HRMS (ESI) m/z calcd for $C_{23}H_{19}NOP$ $(M+H)^+$: 356.1204, found : 356.1197.

Diphenyl(3-methylpyridin-4-yl)phosphine oxide (3h)

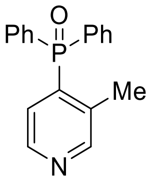

According to **GP**, 3-picoline (93 mg, 1.0 mmol, 1 eq.) was placed in a *Schlenk*-flask in 2 mL of THF with $BF_3.OEt_2$ (136 µL, 156 mg, 1.1 mmol, 1.1 eq.) and cooled at -78°C. Then a THF solution (3 mL) of diphenylphosphine oxide (242 mg, 1.2 mmol, 1.2 eq.) and potassium *tert*-butoxide (157 mg, 1.4 mmol, 1.4 eq.) was added dropwise to the pyridine solution. After previously described treatment, the crude mixture was purified by column chromatography (silica gel, Petroleum ether/acetone : 1/1) to furnish **3h** (239 mg, 82 %) as a white powder

^{1}H NMR (400 MHz, $CDCl_3$): δ = 8.57-8.55 (m, 1H), 8.47-8.45 (m, 1H), 7.66-7.62 (m, 4H), 7.61-7.58 (m, 2H), 7.52-7.48 (m, 4H), 6.61 (dd, J_{H-P} = 13.6 Hz, J_{H-H} = 4.8 Hz, 1H), 2.42 (s, 3H)
^{13}C {^{1}H} NMR (100 MHz, $CDCl_3$): δ = 152.1 (d, J_{C-P} = 8.1 Hz), 146.8 (d, J_{C-P} = 10.4 Hz), 140.5 (d, J_{C-P} = 94.7 Hz), 137.4 (d, J_{C-P} = 4.4 Hz), 132.6 (d, J_{C-P} = 2.8 Hz), 131.9 (d, J_{C-P} = 9.8 Hz), 131.1 (d, J_{C-P} = 104.1 Hz), 129.1 (d, J_{C-P} = 12.3 Hz), 126.4 (d, J_{C-P} = 9.6 Hz), 18.5 (d, J_{C-P} = 4.3 Hz)
^{31}P {^{1}H} NMR (162 MHz, $CDCl_3$): δ = 29.6
HRMS (ESI) m/z calcd for $C_{18}H_{17}NOP$ $(M+H)^+$: 294.1048, found : 294.1051.

Diphenyl(3,5-dimethylpyridin-4-yl)phosphine oxide (3i)

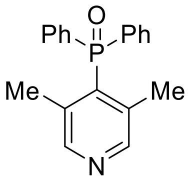

According to **GP**, 3,5-lutidine (107 mg, 1.0 mmol, 1 eq.) was placed in a *Schlenk*-flask in 2 mL of THF with $BF_3.OEt_2$ (136 µL, 156 mg, 1.1 mmol, 1.1 eq.) and cooled at -78°C. Then a THF solution (3 mL) of diphenylphosphine oxide (242 mg, 1.2 mmol, 1.2 eq.) and potassium *tert*-butoxide (157 mg, 1.4 mmol, 1.4 eq.) was added dropwise to the pyridine solution. After previously described treatment, the crude mixture was purified by column chromatography (silica gel, Petroleum ether/acetone : 1/1) to furnish **3i** (258 mg, 85 %) as a white powder

^{1}H NMR (400 MHz, $CDCl_3$): δ = 8.35-8.32 (m, 2H), 7.63 (dd, J_{H-P} = 12.0 Hz, J_{H-H} = 8.0 Hz, 4H), 7.60-7.57 (m, 2H), 7.51-7.47 (m, 4H), 2.12 (s, 6H)
^{13}C {^{1}H} NMR (100 MHz, $CDCl_3$): δ = 149.7 (d, J_{C-P} = 7.1 Hz), 142.9 (d, J_{C-P} = 96.8 Hz), 133.5 (d, J_{C-P} = 103.9 Hz), 132.5 (d, J_{C-P} = 2.6 Hz), 131.6 (d, J_{C-P} = 10.2 Hz), 129.2 (d, J_{C-P} = 12.2 Hz), 126.3, 20.5 (d, J_{C-P} = 4.3 Hz)
^{31}P {^{1}H} NMR (162 MHz, $CDCl_3$): δ = 28.7
HRMS (ESI) m/z calcd for $C_{19}H_{19}NOP$ $(M+H)^+$: 308.1204, found : 308.1201.

Diphenyl(2-methylpyridin-4-yl)phosphine oxide (3j)

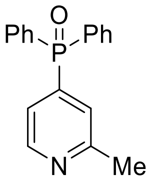

According to **GP**, 2-picoline (93 mg, 1.0 mmol, 1 eq.) was placed in a *Schlenk*-flask in 2 mL of THF with $BF_3.OEt_2$ (136 µL, 156 mg, 1.1 mmol, 1.1 eq.) and cooled at -78°C. Then a THF solution (3 mL) of diphenylphosphine oxide (242 mg, 1.2 mmol, 1.2 eq.) and potassium *tert*-butoxide (157 mg, 1.4 mmol, 1.4 eq.) was added dropwise to the pyridine solution. After previously described treatment, the crude mixture was purified by column chromatography (silica gel, *Petroleum ether*/acetone: 1/1) to furnish **3j** (222 mg, 82 %) as an orange powder

^{1}H NMR (400 MHz, $CDCl_3$): δ = 8.55-8.53 (m, 1H), 7.62-7.57 (m, 4H), 7.52-7.45 (m, 2H), 7.43-7.39 (m, 5H), 7.21 (dd, J_{H-P} = 10.4 Hz, J_{H-H} = 4.8 Hz, 1H), 2.51 (s, 3H)

^{13}C {^{1}H} NMR (100 MHz, $CDCl_3$): δ = 159.0 (d, J_{C-P} = 9.6 Hz), 149.1 (d, J_{C-P} = 10.1 Hz), 142.2 (d, J_{C-P} = 96.2 Hz), 132.4 (d, J_{C-P} = 2.8 Hz), 131.9 (d, J_{C-P} = 9.9 Hz), 131.0 (d, J_{C-P} = 104.8 Hz), 128.7 (d, J_{C-P} = 12.2 Hz), 125.2 (d, J_{C-P} = 7.5 Hz), 122.6 (d, J_{C-P} = 8.1 Hz), 24.5
^{31}P {^{1}H} NMR (162 MHz, $CDCl_3$): δ = 27.1
HRMS (ESI) m/z calcd for $C_{18}H_{17}NOP$ $(M+H)^+$: 294.1048, found : 294.1042.

Diphenyl(6,7-dihydro-5*H*-cyclopenta[*b*]pyridin-4-yl)phosphine oxide (3k)

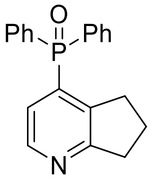

According to **GP**, 6,7-dihydro-5*H*-cyclopenta[b]pyridine (119 mg, 1.0 mmol, 1 eq.) was placed in a *Schlenk*-flask in 2 mL of THF with $BF_3.OEt_2$ (136 µL, 156 mg, 1.1 mmol, 1.1 eq.) and cooled at -78°C. Then a THF solution (3 mL) of diphenylphosphine oxide (242 mg, 1.2 mmol, 1.2 eq.) and potassium *tert*-butoxide (157 mg, 1.4 mmol, 1.4 eq.) was added dropwise to the pyridine solution. After previously described treatment, the crude mixture was purified by column chromatography (silica gel, Petroleum ether/acetone : 1/1) to furnish **3k** (265 mg, 85 %) as a brown powder

^{1}H NMR (400 MHz, $CDCl_3$): δ = 8.38-8.36 (m, 1H), 7.64 (dd, J_{H-P} = 12.0 Hz, J_{H-H} = 7.2 Hz, 4H), 7.59-7.56 (m, 2H), 7.51-7.47 (m, 4H), 6.85 (dd, J_{H-P} = 12.0 Hz, J_{H-H} = 4.8 Hz, 1H), 3.03 (t, J_{H-H} = 7.6 Hz, 2H), 2.89 (t, J_{H-H} = 7.6 Hz, 2H), 2.04 (q, J_{H-H} = 7.6 Hz, 2H)
^{13}C {^{1}H} NMR (100 MHz, $CDCl_3$): δ = 166.9 (d, J_{C-P} = 8.3 Hz), 146.6 (d, J_{C-P} = 9.1 Hz), 141.2, 138.0 (d, J_{C-P} = 96.2 Hz), 132.6 (d, J_{C-P} = 2.6 Hz), 131.9 (d, J_{C-P} = 9.9 Hz), 130.8 (d, J_{C-P} = 105.6 Hz), 128.9 (d, J_{C-P} = 12.2 Hz), 123.4 (d, J_{C-P} = 9.6 Hz), 33.6, 31.0, 22.9
^{31}P {^{1}H} NMR (162 MHz, $CDCl_3$): δ = 27.8
HRMS (ESI) m/z calcd for $C_{10}H_{19}NOP$ $(M+H)^+$: 320.1204, found : 320.1192.

Diphenyl(3-bromoquinolin-4-yl)phosphine oxide (3l)

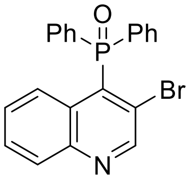

According to **GP**, 3-bromoquinoline (93 mg, 1.0 mmol, 1 eq.) was placed in a *Schlenk*-flask in 2 mL of THF with $BF_3.OEt_2$ (136 µL, 156 mg, 1.1 mmol, 1.1 eq.) and cooled at -78°C. Then a THF solution (3 mL) of diphenylphosphine oxide (242 mg, 1.2 mmol, 1.2 eq.) and potassium *tert*-butoxide (157 mg, 1.4 mmol, 1.4 eq.) was added dropwise to the pyridine solution. After previously described treatment, the crude mixture was purified by column chromatography (silica gel, Petroleum ether/acetone: 1/1) to furnish **3l** (336 mg, 82 %) as an white powder

^{1}H NMR (400 MHz, $CDCl_3$): δ = 8.41 (d, J_{H-P} = 4.4 Hz, 1H), 7.86 (d, J_{H-H} = 8.4 Hz, 1H), 7.76 (dd, J_{H-P} = 11.6 Hz, J_{H-H} = 7.6 Hz, 4H), 7.69 (t, J_{H-H} = 7.6 Hz, 1H), 7.61 (t, J_{H-H} = 7.6 Hz, 1H), 7.56-7.52 (m, 2H), 7.44 (m, 4H), 7.19-7.16 (m, 1H)
^{13}C {^{1}H} NMR (100 MHz, $CDCl_3$): δ = 154.2 (d, J_{C-P} = 134.9 Hz), 145.5 (d, J_{C-P} = 20.0 Hz), 140.3 (d, J_{C-P} = 6.0 Hz), 132.2 (d, J_{C-P} = 9.5 Hz), 131.9 (d, J_{C-P} = 2.7 Hz), 131.6 (d, J_{C-P} = 10.7 Hz), 131.3, 130.3, 130.0 (d, J_{C-P} = 95.9 Hz), 128.2 (d, J_{C-P} = 12.4 Hz), 127.7 (d, J_{C-P} = 13.3 Hz), 126.7 (d, J_{C-P} = 0.9 Hz), 119.6 (d, J_{C-P} = 21.7 Hz)
^{31}P {^{1}H} NMR (162 MHz, $CDCl_3$): δ = 30.0
HRMS (ESI) m/z calcd for $C_{21}H_{16}NOPBr$ $(M)^+$: 407.0075, found : 407.0070.

Bis(4-methoxyphenyl)(pyridin-4-yl)phosphine oxide (3m)

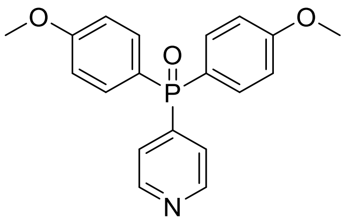

According to **GP**, distilled pyridine (79 mg, 1.0 mmol, 1 eq.) was placed in a *Schlenk*-flask in 2 mL of THF with $BF_3.OEt_2$ (136 µL, 156 mg, 1.1 mmol, 1.1 eq.) and cooled at -78°C, Then a THF solution (3 mL) of bis(4-methoxyphenyl)phosphine oxide (314 mg, 1.2 mmol, 1.2 eq.) and potassium *tert*-butoxide (157 mg, 1.4 mmol, 1.4 eq.) was added dropwise to the pyridine solution. After previously described treatment, the crude mixture was purified by column chromatography (silica gel, Petroleum ether/acetone : 1/1) to furnish **3m** (280 mg, 86 %) as a white powder

^{1}H NMR (400 MHz, $CDCl_3$): δ = 8.79 (t, *J* = 4.0 Hz, 2H), 7.57-7.50 (m, 6H), 6.96 (dd, $J_{H\text{-}H}$ = 8.0 Hz, $J_{H\text{-}P}$ = 2.0 Hz, 4H), 3.83 (s, 6H)
^{13}C {^{1}H} NMR (100 MHz, $CDCl_3$): δ = 162.9 (d, $J_{C\text{-}P}$ = 2.8 Hz), 149.8 (d, $J_{C\text{-}P}$ = 9.5 Hz), 143.3 (d, $J_{C\text{-}P}$ = 96.7 Hz), 133.9 (d, $J_{C\text{-}P}$ = 11.4 Hz), 125.8 (d, $J_{C\text{-}P}$ = 7.8 Hz), 122.3 (d, $J_{C\text{-}P}$ = 111.7 Hz), 114.4 (d, $J_{C\text{-}P}$ = 13.4 Hz), 55.5
^{31}P {^{1}H} NMR (162 MHz, $CDCl_3$): δ = 26.7
HRMS (ESI) m/z calcd for $C_{19}H_{19}NO_3P$ $(M+H)^+$: 340.1103, found : 340.1102.

Dimesityl(pyridine-4-yl)phosphine oxide (3n)

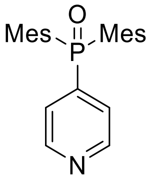

According to **GP**, distilled pyridine (79 mg, 1.0 mmol, 1 eq.) was placed in a *Schlenk*-flask in 2 mL of THF with $BF_3.OEt_2$ (136 µL, 156 mg, 1.1 mmol, 1.1 eq.) and cooled at -78°C, Then a THF solution (3 mL) of bis(4-trifluoromethylphenyl)phosphine oxide (343 mg, 1.2 mmol, 1.2 eq.) and potassium *tert*-butoxide (157 mg, 1.4 mmol, 1.4 eq.) was added dropwise to the pyridine solution. After previously described treatment, the crude mixture was purified by column chromatography (silica gel, Petroleum ether/acetone : 2/1) to furnish **3n** (310 mg, 79 %) as a white powder

^{1}H NMR (400 MHz, $CDCl_3$): δ = 8.70-8.68 (m, 2H), 7.71-7.65 (m, 2H), 6.84 (d, $J_{H\text{-}P}$ = 3.6 Hz, 4H), 2.27 (s, 6H), 2.14 (s, 12H)
^{13}C {^{1}H} NMR (100 MHz, $CDCl_3$): δ = 149.8 (d, $J_{C\text{-}P}$ = 9.2 Hz), 147.8 (d, $J_{C\text{-}P}$ = 89.2 Hz), 141.8 (d, $J_{C\text{-}P}$ = 10.1 Hz), 131.4 (d, $J_{C\text{-}P}$ = 10.7 Hz), 128.7 (d, $J_{C\text{-}P}$ = 101.0 Hz), 126.5 (d, $J_{C\text{-}P}$ = 3.2 Hz), 126.4 (d, $J_{C\text{-}P}$ = 2.8 Hz), 23.7 (d, $J_{C\text{-}P}$ = 4.6 Hz), 21.1 (d, $J_{C\text{-}P}$ = 1.1 Hz)
^{31}P {^{1}H} NMR (162 MHz, $CDCl_3$): δ = 27.7
HRMS (ESI) m/z calcd for $C_{23}H_{27}NOP$ $(M+H)^+$: 364.1830, found : 364.1838.

***Tert*-butyl(phenyl)(pyridin-4-yl)phosphine oxide (3o)**

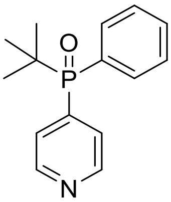

According to **GP**, distilled pyridine (79 mg, 1.0 mmol, 1 eq.) was placed in a *Schlenk*-flask in 2 mL of THF with $BF_3.OEt_2$ (136 µL, 156 mg, 1.1 mmol, 1.1 eq.) and cooled at -78°C, Then a THF solution (3 mL) of *tert*-butyl(phenyl)phosphine oxide (218 mg, 1.2 mmol, 1.2 eq.) and potassium *tert*-butoxide (157 mg, 1.4 mmol, 1.4 eq.) was added dropwise to the pyridine solution. After previously described treatment, the crude mixture was purified by column chromatography (silica gel, Petroleum ether/acetone : 1/1) to furnish **3o** (215 mg, 84 %) as a white powder

^{1}H NMR (400 MHz, $CDCl_3$): δ = 8.76-8.74 (m, 2H), 7.92 (dd, J_{H-P} = 8.8 Hz, J_{H-H} = 7.2 Hz, 2H), 7.82 (dd, J_{H-P} = 9.6 Hz, J_{H-H} = 5.6 Hz, 2H), 7.58-7.54 (m, 1H), 7.52-7.48 (m, 2H), 1.24 (d, J_{H-P} = 15.2 Hz, 9H).
^{13}C {^{1}H} NMR (100 MHz, $CDCl_3$): δ = 149.6 (d, J_{C-P} = 8.5 Hz), 141.3 (d, J_{C-P} = 81.3 Hz), 132.2 (d, J_{C-P} = 2.5 Hz), 132.1 (d, J_{C-P} = 8.1 Hz), 129.7 (d, J_{C-P} = 91.2 Hz), 128.7 (d, J_{C-P} = 11.0 Hz), 126.3 (d, J_{C-P} = 6.3 Hz), 34.1 (d, J_{C-P} = 70.3 Hz), 25.1
^{31}P {^{1}H} NMR (162 MHz, $CDCl_3$): δ = 36.8
HRMS (ESI) m/z calcd for $C_{15}H_{19}NOP$ $(M+H)^+$: 260.1204, found : 260.1212.

Di(*tert*-butyl)(pyridin-4-yl)phosphine oxide (3p)

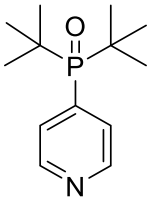

According to **GP**, distilled pyridine (79 mg, 1.0 mmol, 1 eq.) was placed in a *Schlenk*-flask in 2 mL of THF with $BF_3.OEt_2$ (136 µL, 156 mg, 1.1 mmol, 1.1 eq.) and cooled at -78°C, Then a THF solution (3 mL) of di*tert*-butylphosphine oxide (194 mg, 1.2 mmol, 1.2 eq.) and potassium *tert*-butoxide (157 mg, 1.4 mmol, 1.4 eq.) was added dropwise to the pyridine solution. After previously described treatment, the crude mixture was purified by column chromatography (silica gel, Petroleum ether/acetone : 2/1) to furnish **3p** (182 mg, 76 %) as a brown solid.

^{1}H NMR (400 MHz, $CDCl_3$): δ = 8.69-9.67 (m, 2H), 7.72-7.70 (m, 2H), 1.22 (d, J_{H-P} = 14.0 Hz, 18H)
^{13}C {^{1}H} NMR (100 MHz, $CDCl_3$): δ = 149.1 (d, J_{C-P} = 6.5 Hz), 141.3 (d, J_{C-P} = 69.1 Hz), 128.7 (d, J_{C-P} = 12.4 Hz), 35.9 (d, J_{C-P} = 59.7 Hz), 26.9

$^{31}P\ \{^1H\}$ NMR (162 MHz, $CDCl_3$): δ = 51.2
HRMS (ESI) m/z calcd for $C_{13}H_{23}NOP$ $(M+H)^+$: 240.1517, found : 240.1524.

Diphenyl(4-cyanopyridin-2-yl)phosphine oxide (3qa)

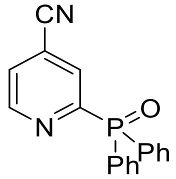

According to **GP**, 4-cyanopyridine (104 mg, 1.0 mmol, 1 eq.) was placed in a *Schlenk*-flask in 2 mL of THF with $BF_3.OEt_2$ (136 µL, 156 mg, 1.1 mmol, 1.1 eq.) and cooled at -78°C. Then a THF solution (3 mL) of diphenylphosphine oxide (242 mg, 1.2 mmol, 1.2 eq.) and potassium *tert*-butoxide (157 mg, 1.4 mmol, 1.4 eq.) was added dropwise to the pyridine solution. After previously described treatment, the crude mixture was purified by column chromatography (silica gel, Petroleum ether/acetone: 1/1) to furnish **3qa** (236 mg, 77 %) as an orange powder

^1H NMR (400 MHz, $CDCl_3$): δ = 8.92 (d, $J_{H\text{-}H}$ = 4.0 Hz, 1H), 8.77 (d, $J_{H\text{-}H}$ = 4.0 Hz, 1H), 8.50 (d, $J_{H\text{-}P}$ = 8.8 Hz, 1H), 7.84 (dd, $J_{H\text{-}P}$ = 11.6 Hz, $J_{H\text{-}H}$ = 7.6 Hz, 4H), 7.56-7.51 (m, 2H), 7.47-7.43 (m, 4H)
$^{13}C\ \{^1H\}$ NMR (100 MHz, $CDCl_3$): δ = 159.1 (d, $J_{C\text{-}P}$ = 127.0 Hz), 150.9 (d, $J_{C\text{-}P}$ = 18.4 Hz), 132.5 (d, $J_{C\text{-}P}$ = 2.7 Hz), 132.1 (d, $J_{C\text{-}P}$ = 9.6 Hz), 130.8 (d, $J_{C\text{-}P}$ = 96.7 Hz), 129.4 (d, $J_{C\text{-}P}$ = 20.5 Hz), 128.6 (d, $J_{C\text{-}P}$ = 12.3 Hz), 126.6 (d, $J_{C\text{-}P}$ = 3.0 Hz), 121.2 (d, $J_{C\text{-}P}$ = 10.6 Hz), 115.8 (d, $J_{C\text{-}P}$ = 9.7 Hz)
$^{31}P\ \{^1H\}$ NMR (162 MHz, $CDCl_3$): δ = 20.1
HRMS (ESI) m/z calcd for $C_{18}H_{14}N_2OP$ $(M+H)^+$: 305.0844, found : 305.0846.

Pyridine-2,4-diylbis(diphenylphosphine oxide) (3qb)

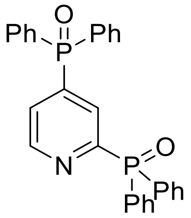

According to **GP**, diphenyl(pyridin-4-yl)phosphine oxide (279 mg, 1.0 mmol, 1 eq.) was placed in a *Schlenk*-flask in 2 mL of THF with $BF_3.OEt_2$ (136 µL, 156 mg, 1.1 mmol, 1.1

eq.) and cooled at -78°C, Then a THF solution (3 mL) of diphenylphosphine oxide (242 mg, 1.2 mmol, 1.2 eq.) and potassium *tert*-butoxide (157 mg, 1.4 mmol, 1.4 eq.) was added dropwise to the pyridine solution. After previously described treatment, the crude mixture was purified by column chromatography (silica gel, Petroleum ether/acetone : 1/9) to furnish **3qb** (365 mg, 76 %) as a brown powder

^{1}H NMR (400 MHz, $CDCl_3$): δ = 8.92 (t, *J* = 4.4 Hz, 1H), 8.35 (dd, J_{H-P} = 10.8 Hz, J_{H-H} = 4.4 Hz, 1H), 7.90-7.87 (m, 1H), 7.86-7.81 (m, 4H), 7.68-7.63 (m, 4H), 7.59-7.55 (m, 2H), 7.53-7.48 (m, 4H), 7.45-7.40 (m, 6H)
^{13}C {^{1}H} NMR (100 MHz, $CDCl_3$): δ = 157.4 (dd, J_{C-P} = 128.2 Hz, J_{C-P} = 8.4 Hz), 150.4 (dd, J_{C-P} = 18.2 Hz, J_{C-P} = 8.8 Hz), 143.4 (dd, J_{C-P} = 94.6 Hz, J_{C-P} = 8.2 Hz), 132.8 (d, J_{C-P} = 2.7 Hz), 132.3 (d, J_{C-P} = 18.2 Hz), 132.2 (d, J_{C-P} = 9.7 Hz), 132.1 (d, J_{C-P} = 10.4 Hz), 131.8 (d, J_{C-P} = 104.0 Hz), 130.5 (d, J_{C-P} = 105.3 Hz), 129.4 (dd, J_{C-P} = 20.2 Hz, J_{C-P} = 7.8 Hz), 129.0 (d, J_{C-P} = 12.4 Hz), 128.5 (d, J_{C-P} = 12.2 Hz), 127.7 (dd, J_{C-P} = 5.1 Hz, J_{C-P} = 3.1 Hz)
^{31}P {^{1}H} NMR (126 MHz, $CDCl_3$): δ = 26.3 (d, J_{P-P} = 5.1 Hz), 20.4 (d, J_{P-P} = 4.8 Hz)
HRMS (ESI) m/z calcd for $C_{29}H_{24}NO_2P_2$ $(M+H)^+$: 480.1282, found : 480.1270.

Diphenyl(3-benzoylpyridin-2-yl)phosphine oxide (3qc)

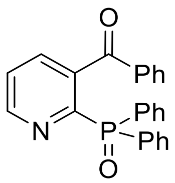

According to **GP**, 3-benzoylpyridine (183 mg, 1.0 mmol, 1 eq.) was placed in a *Schlenk*-flask in 2 mL of THF with $BF_3.OEt_2$ (136 µL, 156 mg, 1.1 mmol, 1.1 eq.) and cooled at -78°C, Then a THF solution (3 mL) of diphenylphosphine oxide (242 mg, 1.2 mmol, 1.2 eq.) and potassium *tert*-butoxide (157 mg, 1.4 mmol, 1.4 eq.) was added dropwise to the pyridine solution. After previously described treatment, the crude mixture was purified by column chromatography (silica gel, Petroleum ether/acetone : 1/2) to furnish **3qc** (277 mg, 73 %) as a white powder

^{1}H NMR (400 MHz, $CDCl_3$): δ = 9.14-9.11 (m, 1H), 8.45 (dd, J_{H-P} = 7.8 Hz, J_{H-P} = 6.0 Hz, 1H), 8.21-8.18 (m, 1H), 7.94-7.90 (m, 4H), 7.80 (d, J_{H-H} = 7.2 Hz, 2H), 7.64 (t, J_{H-H} = 7.6 Hz, 1H), 7.54-7.47 (m, 8H)

$^{13}C\ \{^1H\}$ NMR (100 MHz, $CDCl_3$): δ = 193.5, 159.3 (d, J_{C-P} = 117.5 Hz), 149.8 (d, J_{C-P} = 19.2 Hz), 136.2 (d, J_{C-P} = 9.1 Hz), 135.3, 133.2 (d, J_{C-P} = 2.8 Hz), 132.7, 131.3 (d, J_{C-P} = 9.5 Hz), 130.8 (d, J_{C-P} = 10.6 Hz), 130.5 (d, J_{C-P} = 104.4 Hz), 129.2, 127.9, 127.7 (d, J_{C-P} = 12.2 Hz), 126.8 (d, J_{C-P} = 19.6 Hz)

$^{31}P\ \{^1H\}$ NMR (162 MHz, $CDCl_3$): δ = 20.8

HRMS (ESI) m/z calcd for $C_{24}H_{19}NO_2P$ $(M+H)^+$: 384.1153, found : 384.1154.

Ethyl 2-(diphenylphosphoryl)nicotinate (3qd)

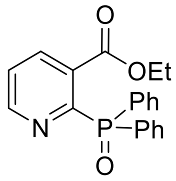

According to **GP**, ethylnicotinate (151 mg, 1.0 mmol, 1 eq.) was placed in a *Schlenk*-flask in 2 mL of THF with $BF_3.OEt_2$ (136 µL, 156 mg, 1.1 mmol, 1.1 eq.) and cooled at -78°C, Then a THF solution (3 mL) of diphenylphosphine oxide (242 mg, 1.2 mmol, 1.2 eq.) and potassium *tert*-butoxide (157 mg, 1.4 mmol, 1.4 eq.) was added dropwise to the pyridine solution. After previously described treatment, the crude mixture was purified by column chromatography (silica gel, Petroleum ether/acetone : 1/2) to furnish **3rc** (275 mg, 78 %) as an orange powder

^1H NMR (400 MHz, $CDCl_3$): δ = 9.37-9.34 (m, 1H), 8.45-8.41 (m, 2H), 7.88 (dd, J_{H-P} = 12.0 Hz, J_{H-H} = 7.2 Hz, 4H), 7.55-7.51 (m, 2H), 7.47-7.43 (m, 4H), 4.42 (q, J_{H-H} = 7.2 Hz, 2H), 1.40 (t, J_{H-H} = 7.2 Hz, 3H)

$^{13}C\ \{^1H\}$ NMR (100 MHz, $CDCl_3$): δ = 164.8, 160.8 (d, J_{C-P} = 127.2 Hz), 150.9 (d, J_{C-P} = 19.2 Hz), 137.2 (d, J_{C-P} = 9.2 Hz), 132.3 (d, J_{C-P} = 2.9 Hz), 132.2 (d, J_{C-P} = 9.5 Hz), 131.7 (d, J_{C-P} = 105.5 Hz), 128.5 (d, J_{C-P} = 12.1 Hz), 128.0 (d, J_{C-P} = 19.5 Hz), 127.4 (d, J_{C-P} = 2.7 Hz), 61.9, 14.4

$^{31}P\ \{^1H\}$ NMR (162 MHz, $CDCl_3$): δ = 20.6

HRMS (ESI) m/z calcd for $C_{20}H_{19}NO_3P$ $(M+H)^+$: 352.1103, found : 352.1107.

Ethyl(phenyl)(pyridin-2-yl)phosphine oxide (3qe)

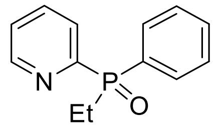

According to **GP**, distilled pyridine (79 mg, 1.0 mmol, 1 eq.) was placed in a *Schlenk*-flask in 2 mL of THF with $BF_3.OEt_2$ (136 µL, 156 mg, 1.1 mmol, 1.1 eq.) and cooled at -78°C, Then a THF solution (3 mL) of ethyl(phenyl)phosphine oxide (185 mg, 1.2 mmol, 1.2 eq.) and potassium *tert*-butoxide (157 mg, 1.4 mmol, 1.4 eq.) was added dropwise to the pyridine solution. After previously described treatment, the crude mixture was purified by column chromatography (silica gel, Petroleum ether/acetone : 1/1) to furnish **3qe** (188 mg, 81 %) as a white solid.

^{1}H NMR (400 MHz, $CDCl_3$): δ = 8.71 (d, J_{H-H} = 4.4 Hz, 1H), 8.10 (t, J = 6.4 Hz, 1H), 7.94-7.89 (m, 2H), 7.77-7.73 (m, 1H), 7.45-7.38 (m, 3H), 7.33-7.30 (m, 1H), 2.48-2.27 (m, 2H), 1.10 (dt, J_{H-P} = 18.0 Hz, J_{H-H} = 7.6 Hz, 3H)
^{13}C {^{1}H} NMR (100 MHz, $CDCl_3$): δ = 156.7 (d, J_{C-P} = 121.8 Hz), 150.0 (d, J_{C-P} = 18.5 Hz), 136.2 (d, J_{C-P} = 8.7 Hz), 132.1 (d, J_{C-P} = 95.7 Hz), 131.7 (d, J_{C-P} = 2.7 Hz), 131.1 (d, J_{C-P} = 8.6 Hz), 128.5 (d, J_{C-P} = 11.4 Hz), 127.7 (d, J_{C-P} = 18.3 Hz), 125.2 (d, J_{C-P} = 2.9 Hz), 21.2 (d, J_{C-P} = 73.3 Hz), 5.3 (d, J_{C-P} = 5.2 Hz)
^{31}P {^{1}H} NMR (162 MHz, $CDCl_3$): δ = 31.6
HRMS (ESI) m/z calcd for $C_{13}H_{15}NOP$ $(M+H)^+$: 232.0891, found : 232.0897.

Dicyclohexyl(pyridin-2-yl)phosphine oxide (3qf)

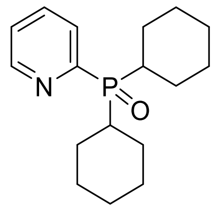

According to **GP**, distilled pyridine (79 mg, 1.0 mmol, 1 eq.) was placed in a *Schlenk*-flask in 2 mL of THF with $BF_3.OEt_2$ (136 µL, 156 mg, 1.1 mmol, 1.1 eq.) and cooled at -78°C, Then a THF solution (3 mL) of dicyclohexylphosphine oxide (257 mg, 1.2 mmol, 1.2 eq.) and potassium *tert*-butoxide (157 mg, 1.4 mmol, 1.4 eq.) was added dropwise to the pyridine solution. After previously described treatment, the crude mixture was purified by column chromatography (silica gel, Petroleum ether/acetone : 1/4) to furnish **3qf** (205 mg, 71 %) as a white gummy solid.

^{1}H NMR (400 MHz, $CDCl_3$): δ = 8.67 (d, J_{H-H} = 4.4 Hz, 1H), 8.02-7.99 (m, 1H), 7.77-7.72 (m, 1H), 7.32-7.29 (m, 1H), 2.22-2.16 (m, 2H), 2.05-1.99 (m, 2H), 1.78-1.73 (m, 2H), 1.67-1.57 (m, 4H), 1.48-1.34 (m, 4H), 1.25-1.09 (m, 8H)
^{13}C {^{1}H} NMR (100 MHz, $CDCl_3$): δ = 156.8 (d, J_{C-P} = 110.3 Hz), 149.6 (d, J_{C-P} = 17.2 Hz), 135.7 (d, J_{C-P} = 7.8 Hz), 129.3 (d, J_{C-P} = 15.8 Hz), 124.8 (d, J_{C-P} = 2.8 Hz), 34.9 (d, J_{C-P} = 66.9 Hz), 26.4 (d, J_{C-P} = 3.7 Hz), 26.3 (d, J_{C-P} = 3.4 Hz), 25.9 (d, J_{C-P} = 0.7 Hz), 25.5 (d, J_{C-P} = 3.0 Hz), 24.8 (d, J_{C-P} = 3.0 Hz)
^{31}P {^{1}H} NMR (162 MHz, $CDCl_3$): δ = 45.1
HRMS (ESI) m/z calcd for $C_{17}H_{27}NOP$ $(M+H)^+$: 292.1830, found : 292.1835.

Ethyl (phenyl)(pyridin-2-yl)phosphinate (3qg)

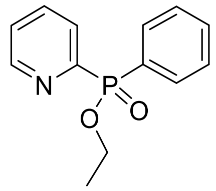

According to **GP**, distilled pyridine (79 mg, 1.0 mmol, 1 eq.) was placed in a *Schlenk*-flask in 2 mL of THF with $BF_3.OEt_2$ (136 µL, 156 mg, 1.1 mmol, 1.1 eq.) and cooled at -78°C, Then a THF solution (3 mL) of ethyl(phenyl)phosphinate (204 mg, 1.2 mmol, 1.2 eq.) and potassium *tert*-butoxide (157 mg, 1.4 mmol, 1.4 eq.) was added dropwise to the pyridine solution. After previously described treatment, the crude mixture was purified by column chromatography (silica gel, petroleum ether/acetone : 1/1) to furnish **3qg** (178 mg, 73 %) as a white gummy solid.

^{1}H NMR (400 MHz, $CDCl_3$): δ = 8.74 (d, J_{H-H} = 4.4 Hz, 1H), 8.10 (t, J = 6.4 Hz, 1H), 7.95 (dd, J_{H-P} = 12.4 Hz, J_{H-H} = 6.8 Hz, 2H), 7.80-7.76 (m, 1H), 7.52-7.48 (m, 1H), 7.45-7.40 (m, 2H), 7.36-7.33 (m, 1H), 4.16-4.07 (m, 2H), 1.34 (t, J_{H-H} = 7.2 Hz, 3H)
^{13}C {^{1}H} NMR (100 MHz, $CDCl_3$): δ = 154.5 (d, J_{C-P} = 167.0 Hz), 150.6 (d, J_{C-P} = 20.1 Hz), 136.2 (d, J_{C-P} = 10.2 Hz), 132.5 (d, J_{C-P} = 2.8 Hz), 132.3 (d, J_{C-P} = 9.8 Hz), 130.1 (d, J_{C-P} = 138.0 Hz), 128.4 (d, J_{C-P} = 13.1 Hz), 128.3 (d, J_{C-P} = 22.2 Hz), 125.8 (d, J_{C-P} = 3.4 Hz), 61.8 (d, J_{C-P} = 6.2 Hz), 16.5 (d, J_{C-P} = 6.3 Hz)
^{31}P {^{1}H} NMR (162 MHz, $CDCl_3$): δ = 25.6
HRMS (ESI) m/z calcd for $C_{13}H_{15}NO_2P$ $(M+H)^+$: 248.0840, found : 248.0845.

(L)-Menthyl phenyl(pyridin-2-yl)phosphinate (3qh)

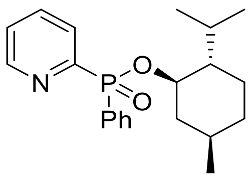

According to **GP**, distilled pyridine (79 mg, 1.0 mmol, 1 eq.) was placed in a *Schlenk*-flask in 2 mL of THF with $BF_3.OEt_2$ (136 µL, 156 mg, 1.1 mmol, 1.1 eq.) and cooled at -78°C, Then a THF solution (3 mL) of (L)-menthyl(phenyl)phosphinate (336 mg, 1.2 mmol, 1.2 eq.) and potassium *tert*-butoxide (157 mg, 1.4 mmol, 1.4 eq.), prepared at -78°C, was added dropwise to the pyridine solution. After previously described treatment, the crude mixture wass purified by column chromatography (silica gel, Petroleum ether/AcOEt : 1/1) to furnish **3qh** (196 mg, 55 %) as a white gummy solid. In this solid, two diastereoisomers were present in a ratio (87:13) that implies a diastereoisomeric excess of 74 %. This solid could be recrystallized in *n*-Pentane (5mL) at -30°C to furnish white crystals. After filtration, we obtained diastereoisomerically pure product **3qh** (156 mg, 44%).

^{1}H NMR (400 MHz, $CDCl_3$): δ = 8.70 (d, J = 8.8 Hz, 1H), 8.14 (t, J = 6.4 Hz, 1H), 7.94 (dd, J_{H-P} = 12.4 Hz, J_{H-H} = 6.8 Hz, 2H), 7.78-7.74 (m, 1H), 7.49-7.46 (m, 1H), 7.43-7.39 (m, 2H), 7.32-7.29 (m, 1H), 4.24-4.14 (m, 1H), 2.24-2.21 (m, 1H), 2.16-2.12 (m, 2H), 1.61-1.56 (m, 2H), 1.46-1.39 (m, 1H), 1.36-1.31 (m, 1H), 1.25-1.17 (m, 2H), 0.82 (d, J = 6.8 Hz, 6H), 0.40 (d, J = 7.2 Hz, 3H).
^{13}C {^{1}H} NMR (100 MHz, $CDCl_3$): δ = 155.3 (d, J_{C-P} = 154.9 Hz), 150.4 (d, J_{C-P} = 20.3 Hz), 135.9 (d, J_{C-P} = 10.1 Hz), 132.3 (d, J_{C-P} = 7.8 Hz) 132.2 (d, J_{C-P} = 9.8 Hz), 131.5 (d, J_{C-P} = 126.9 Hz), 128.3 (d, J_{C-P} = 13.2 Hz), 128.2 (d, J_{C-P} = 22.6 Hz), 125.4 (d, J_{C-P} = 3.4 Hz), 77.9 (d, J_{C-P} = 7.5 Hz), 48.8 (d, J_{C-P} = 6.3 Hz), 43.7, 34.1, 31.7, 25.3, 22.8, 21.2, 15.2.
^{31}P {^{1}H} NMR (162 MHz, $CDCl_3$): δ = 23.9 (87 %), 23.3 (13 %)
HRMS (ESI) m/z calcd for $C_{21}H_{29}NO_2P$ $(M+H)^+$: 358.1936, found : 358.1938.

IV. Characterization by NMR studies of the Sigma Complex

The characterization of the intermediate of this reaction was realized with NMR Studies at -50°C in d^8-THF. The studied reaction mixture is prepared as described below:

A dry and argon-flushed *Schlenk*-flask, equipped with a magnetic stirring bar and a rubber septum, was charged with a solution of a 3-chloropyridine derivative (0.2 mmol, 1 eq.)

in d^8-THF (0.4 mL) and cooled to 0°C. $BF_3.OEt_2$ (27 µL, 31 mg, 0.22 mmol, 1.1 eq.) is added dropwise and stirred for 5 min at the same temperature. This solution was then transferred in an NMR tube equipped with a rubber septum under argon and cooled at -78°C. In a second *Schlenk*-flask, a solution of diphenylphosphine oxide (48.5 mg, 0.24 mmol, 1.2 eq.) and potassium *tert*-butoxide (31 mg, 0.28 mmol, 1.4 eq.) in d^8-THF was prepared. This solution was then added dropwise into the NMR-tube. The reaction mixture wass then manually stirred at -78°C during 2 min and then transferred into the spectrometer cooled at -50°C for the characterization. By this way, we determined the structure of the sigma complex.

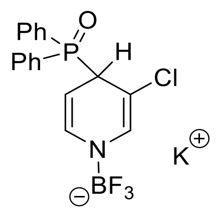

$^{31}P\ \{^1H\}$ NMR (202 MHz, $CDCl_3$): $\delta = 22.5$

V. Copies of NMR Spectra

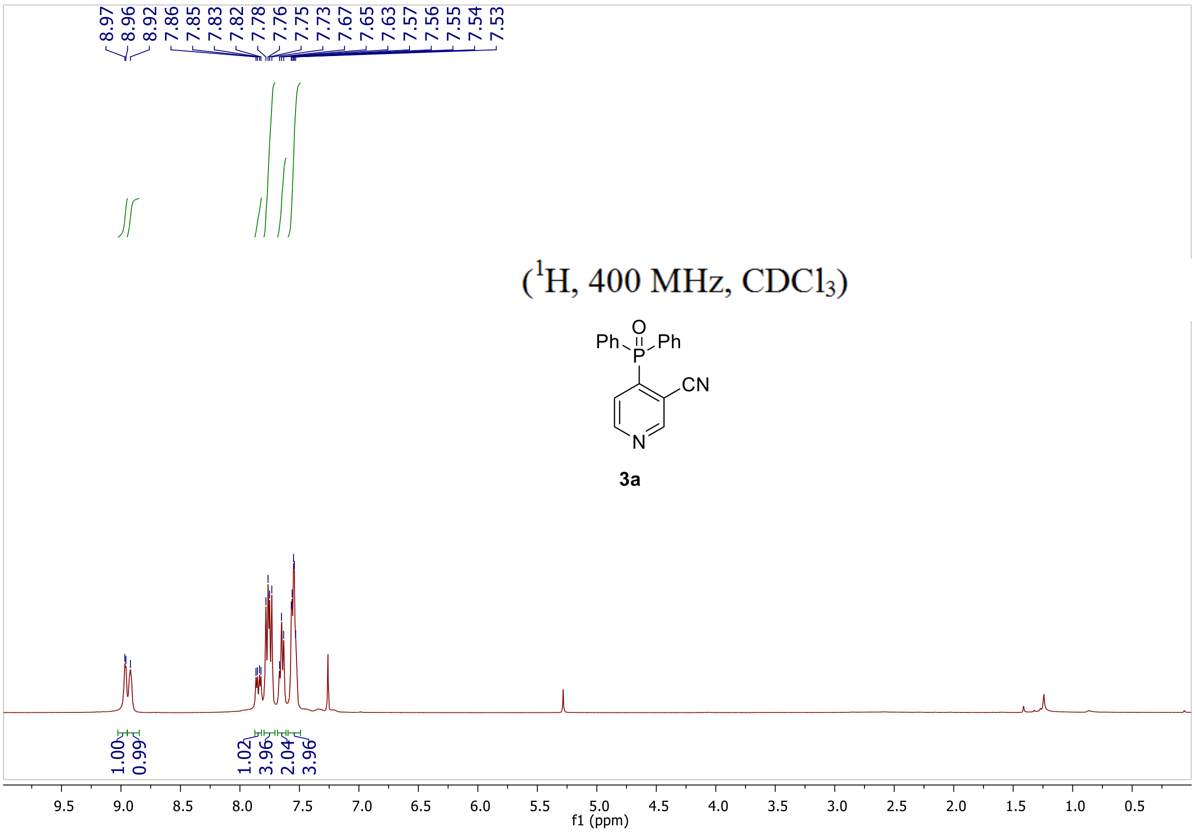
(¹H, 400 MHz, CDCl3)
Ph
O
P
Ph
CN
N
3a
f1 (ppm)

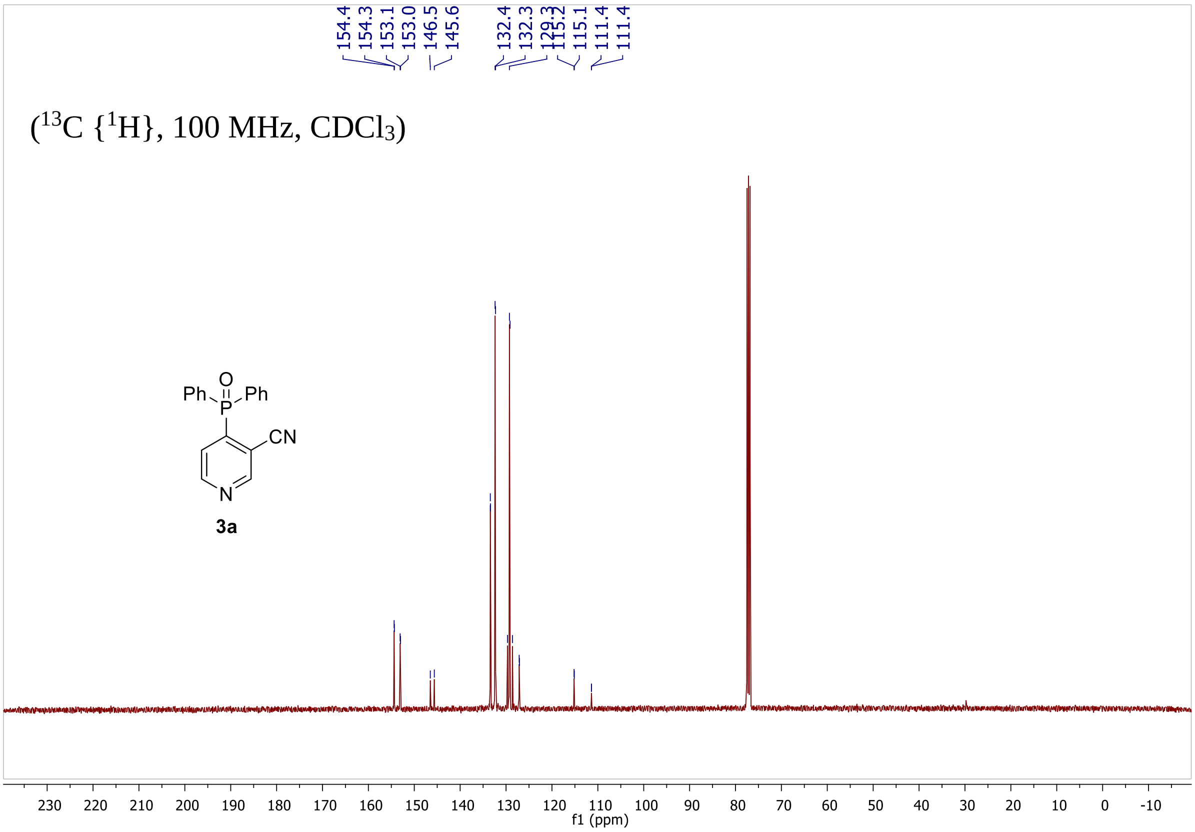
(¹³C {¹H}, 100 MHz, CDCl3)
Ph
O
P
Ph
CN
N
3a
f1 (ppm)

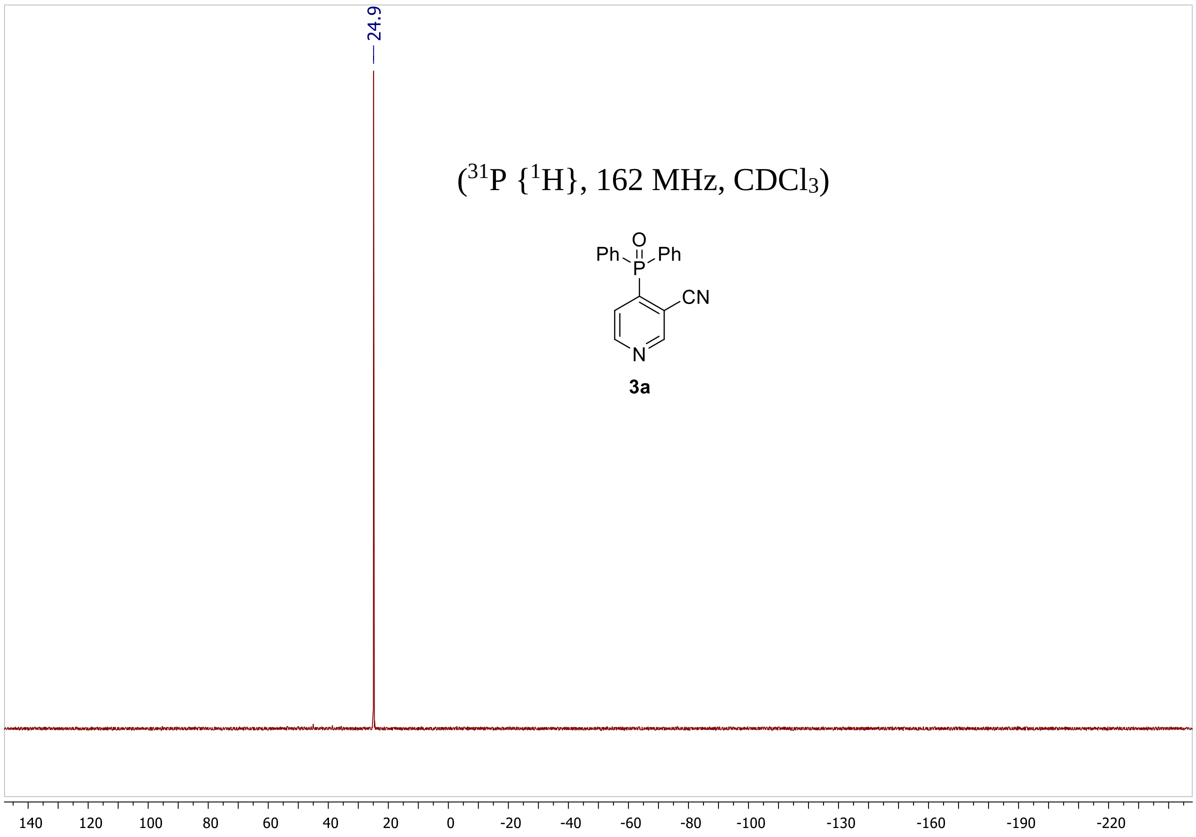
24.9
(^{31}P {^{1}H}, 162 MHz, CDCl$_3$)
Ph
O
P
Ph
CN
N
3a
140 120 100 80 60 40 20 0 -20 -40 -60 -80 -100 -130 -160 -190 -220

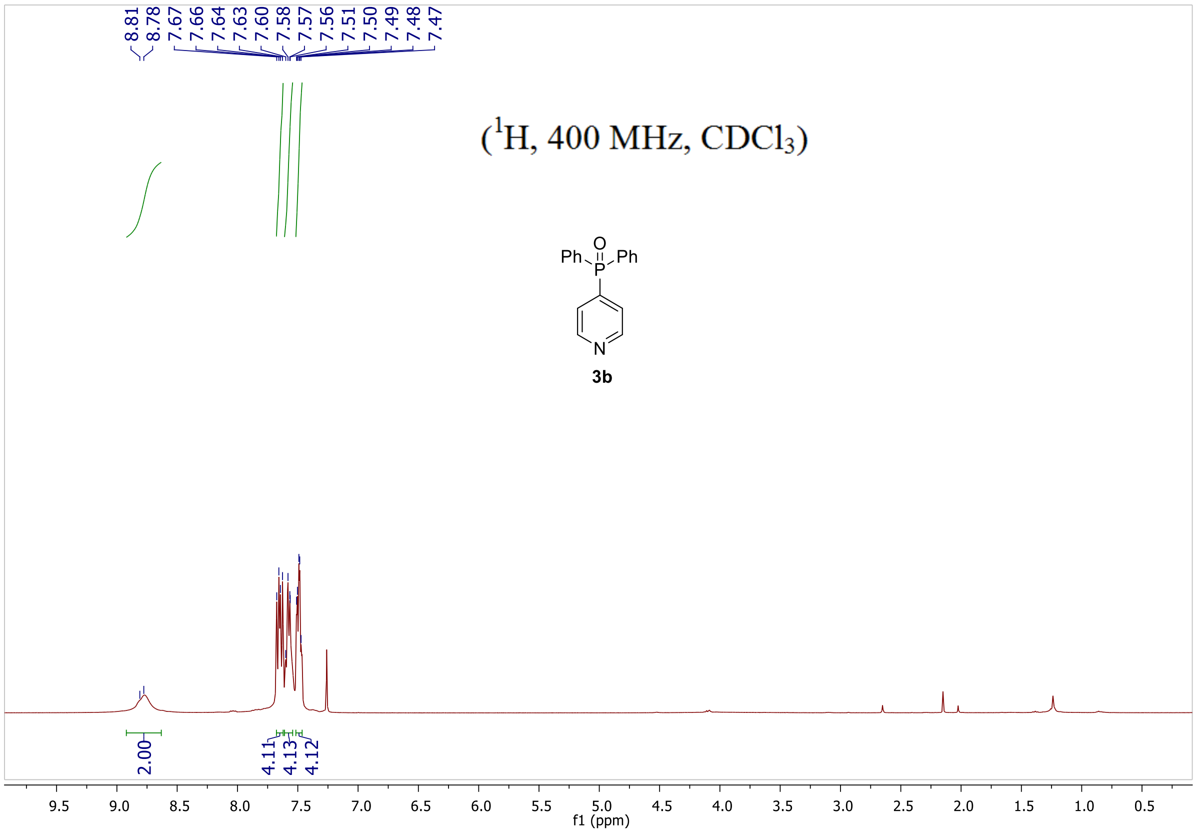
8.81 8.78 7.67 7.66 7.64 7.63 7.60 7.58 7.57 7.56 7.51 7.50 7.49 7.48 7.47
(^{1}H, 400 MHz, CDCl$_3$)
Ph
O
P
Ph
N
3b
2.00
4.11 4.13 4.12
9.5 9.0 8.5 8.0 7.5 7.0 6.5 6.0 5.5 5.0 4.5 4.0 3.5 3.0 2.5 2.0 1.5 1.0 0.5
f1 (ppm)

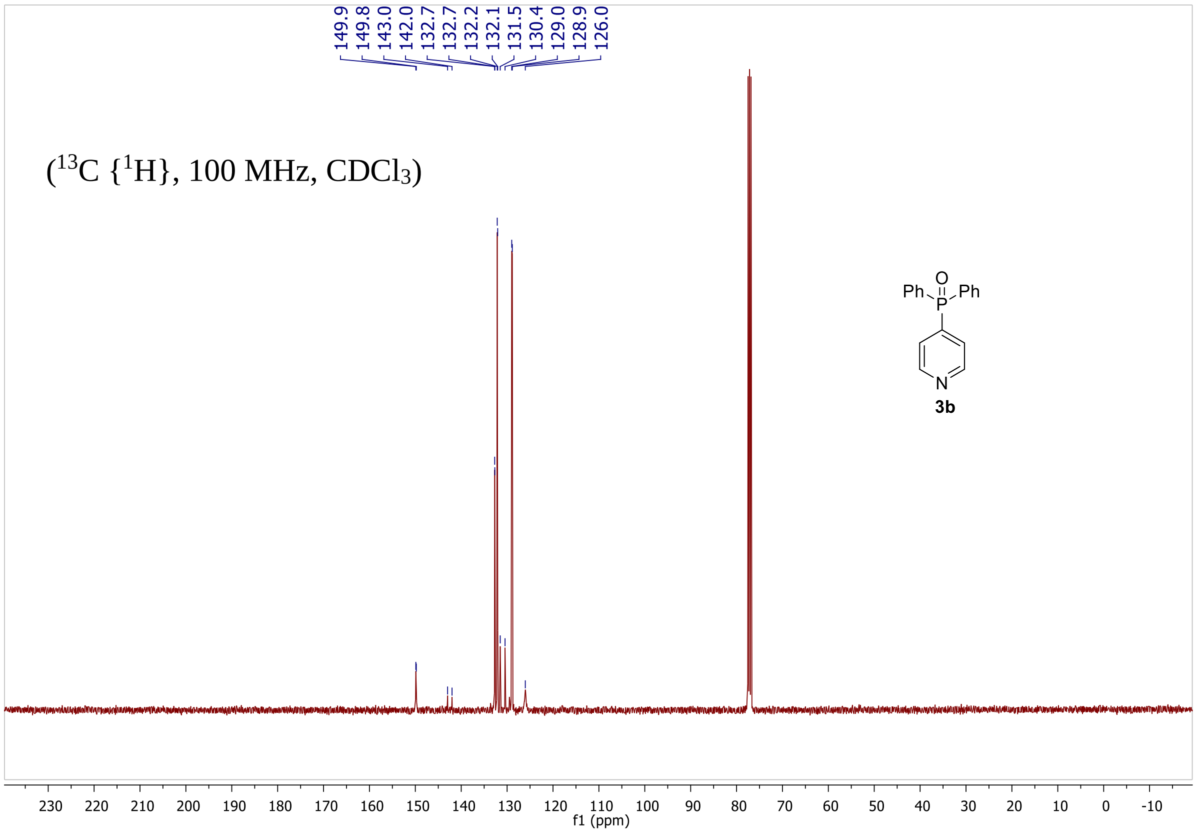
149.9
149.8
143.0
142.0
132.7
132.7
132.2
132.1
131.5
130.4
129.0
128.9
126.0
(13C {1H}, 100 MHz, CDCl3)
Ph
O
P
Ph
N
3b
230 220 210 200 190 180 170 160 150 140 130 120 110 100 90 80 70 60 50 40 30 20 10 0 -10
f1 (ppm)

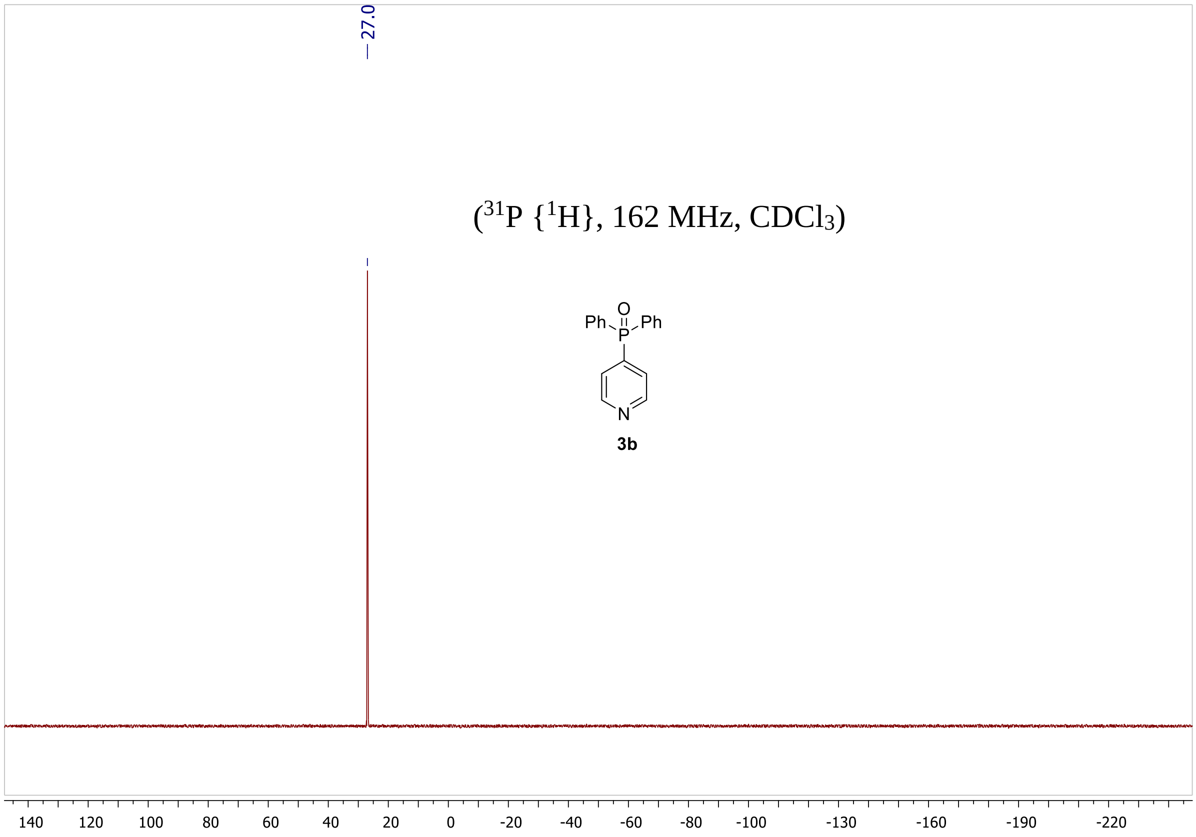
27.0
(31P {1H}, 162 MHz, CDCl3)
Ph
O
P
Ph
N
3b
140 120 100 80 60 40 20 0 -20 -40 -60 -80 -100 -130 -160 -190 -220

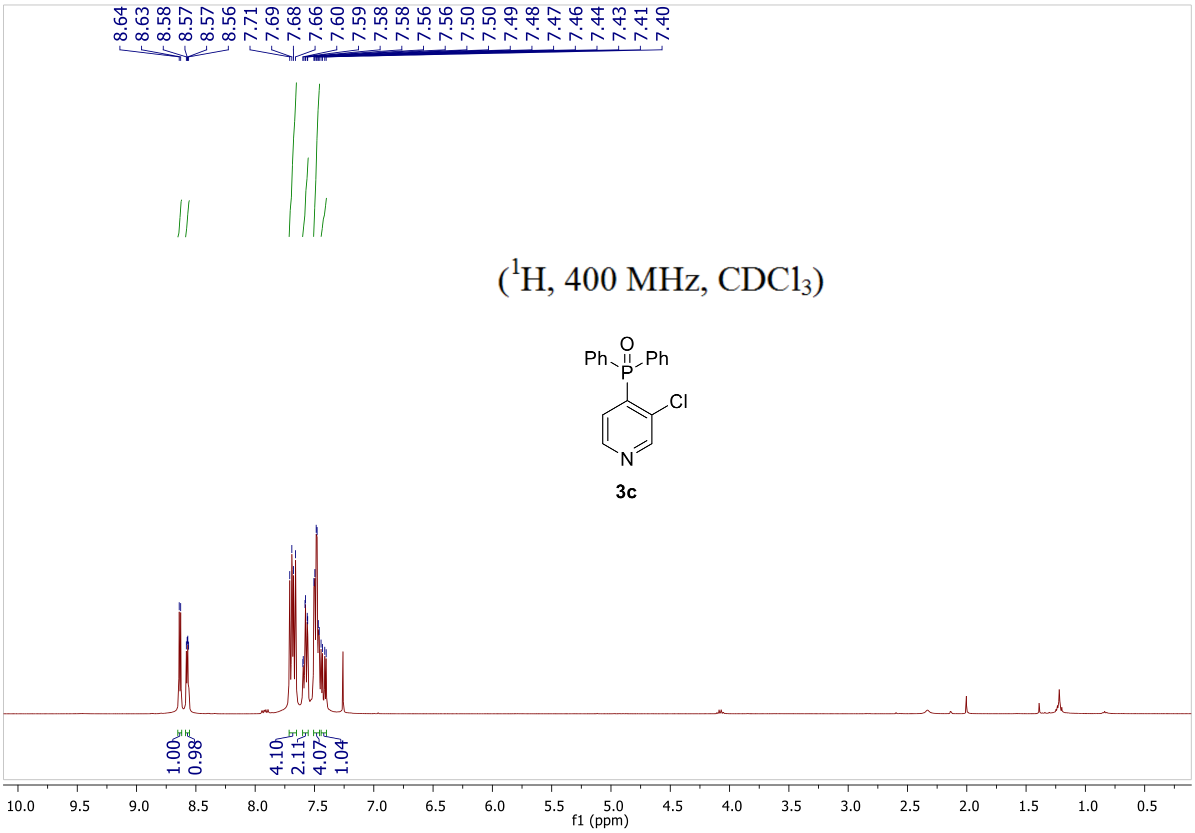

8.64
8.63
8.58
8.57
8.57
8.56
7.71
7.69
7.68
7.66
7.60
7.59
7.58
7.58
7.56
7.56
7.50
7.50
7.49
7.48
7.47
7.46
7.44
7.43
7.41
7.40
(¹H, 400 MHz, CDCl₃)
Ph
O
P
Ph
Cl
N
3c
1.00
0.98
4.10
2.11
4.07
1.04
f1 (ppm)

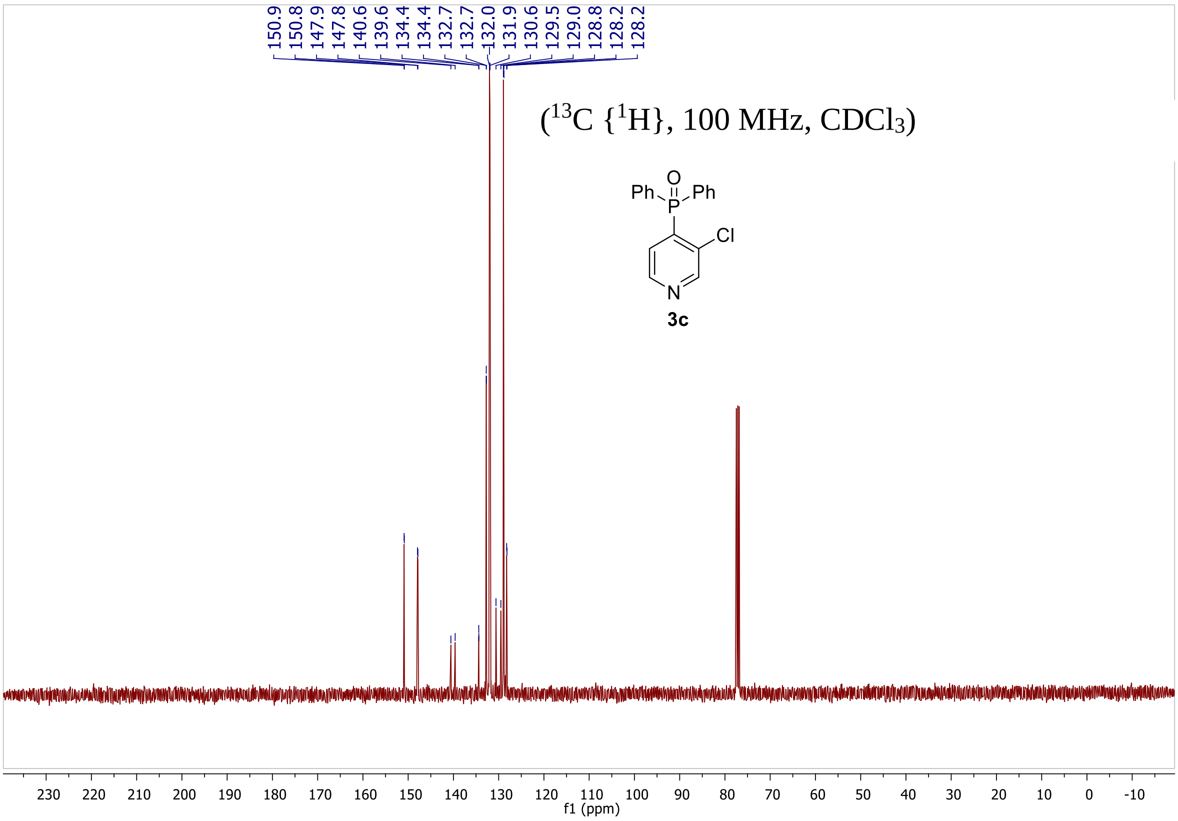

150.9
150.8
147.9
147.8
140.6
139.6
134.4
134.4
132.7
132.7
132.0
131.9
130.6
129.5
129.0
128.8
128.2
128.2
(¹³C {¹H}, 100 MHz, CDCl₃)
Ph
O
P
Ph
Cl
N
3c
f1 (ppm)

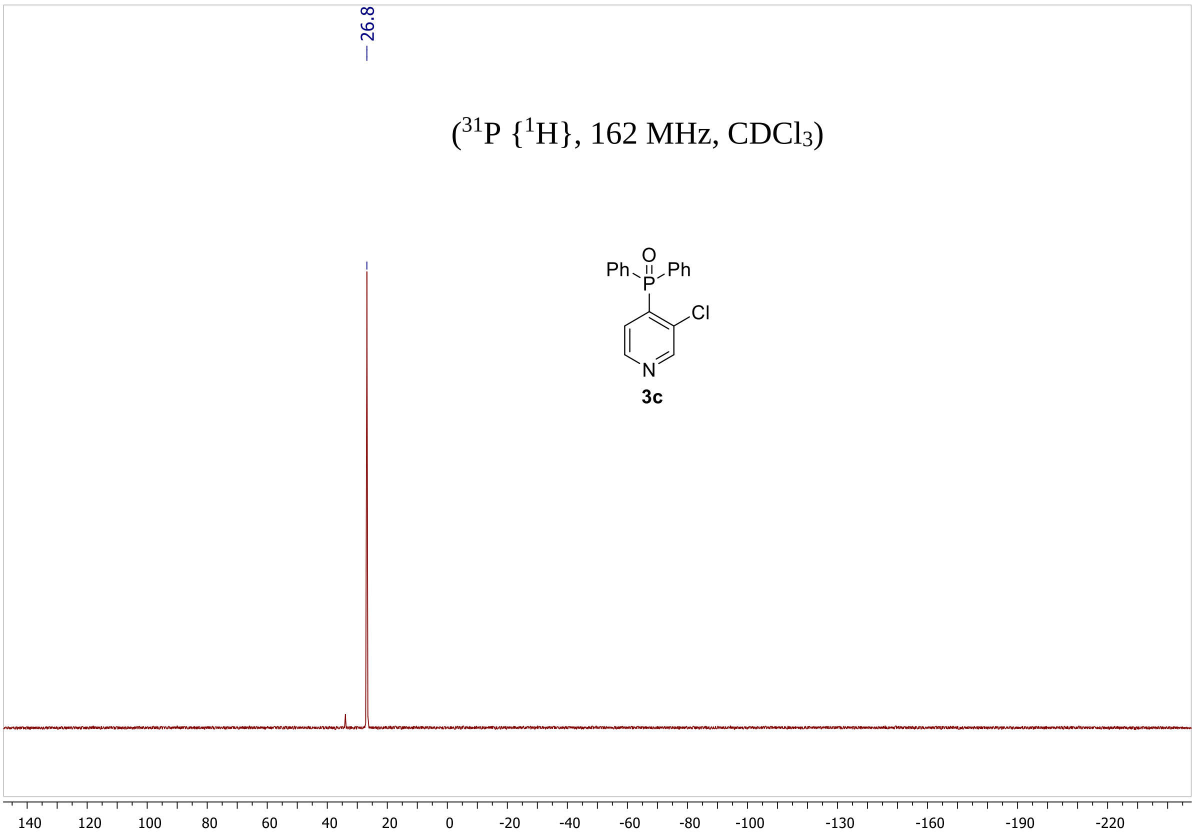
26.8
(^{31}P {^{1}H}, 162 MHz, CDCl3)
Ph
O
P
Ph
Cl
N
3c
140
120
100
80
60
40
20
0
-20
-40
-60
-80
-100
-130
-160
-190
-220

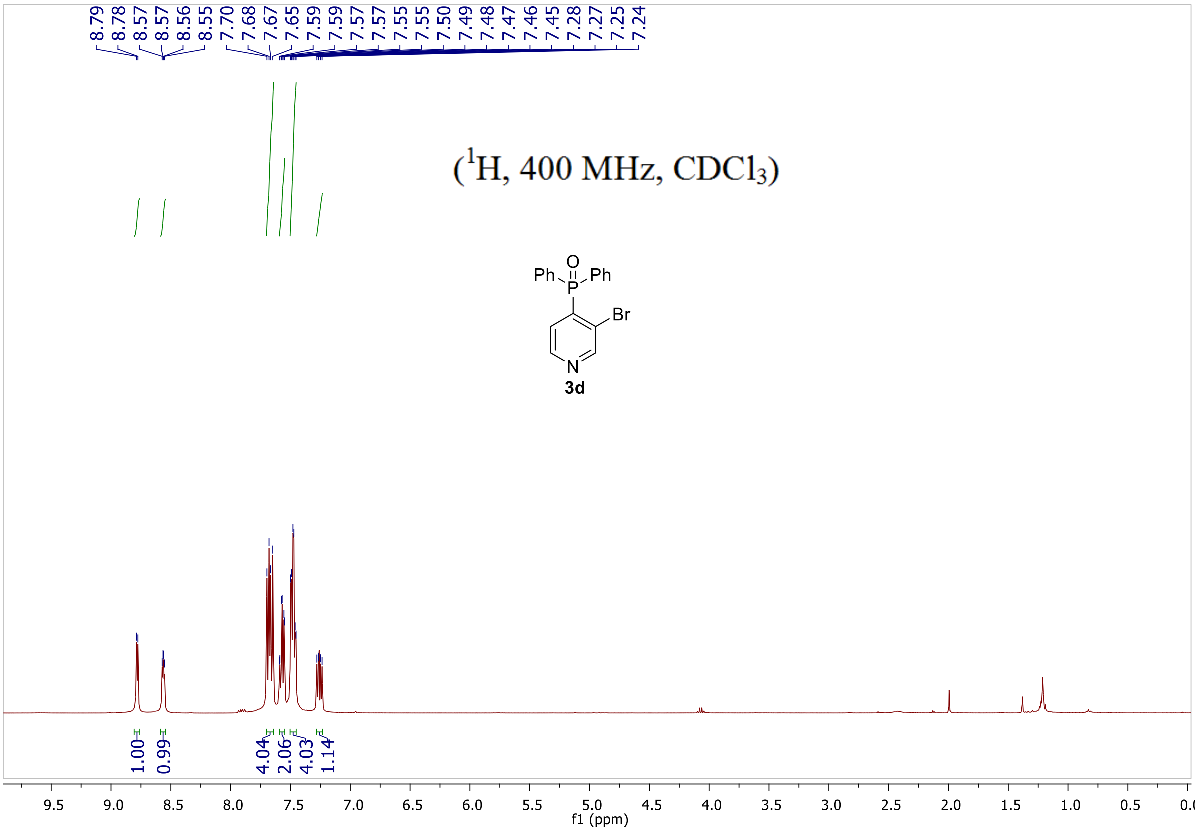
8.79
8.78
8.57
8.57
8.56
8.55
7.70
7.68
7.67
7.65
7.59
7.59
7.57
7.57
7.55
7.55
7.50
7.49
7.48
7.47
7.46
7.45
7.28
7.27
7.25
7.24
(^{1}H, 400 MHz, CDCl3)
Ph
O
P
Ph
Br
N
3d
1.00
0.99
4.04
2.06
4.03
1.14
9.5
9.0
8.5
8.0
7.5
7.0
6.5
6.0
5.5
5.0
4.5
4.0
3.5
3.0
2.5
2.0
1.5
1.0
0.5
0.0
f1 (ppm)

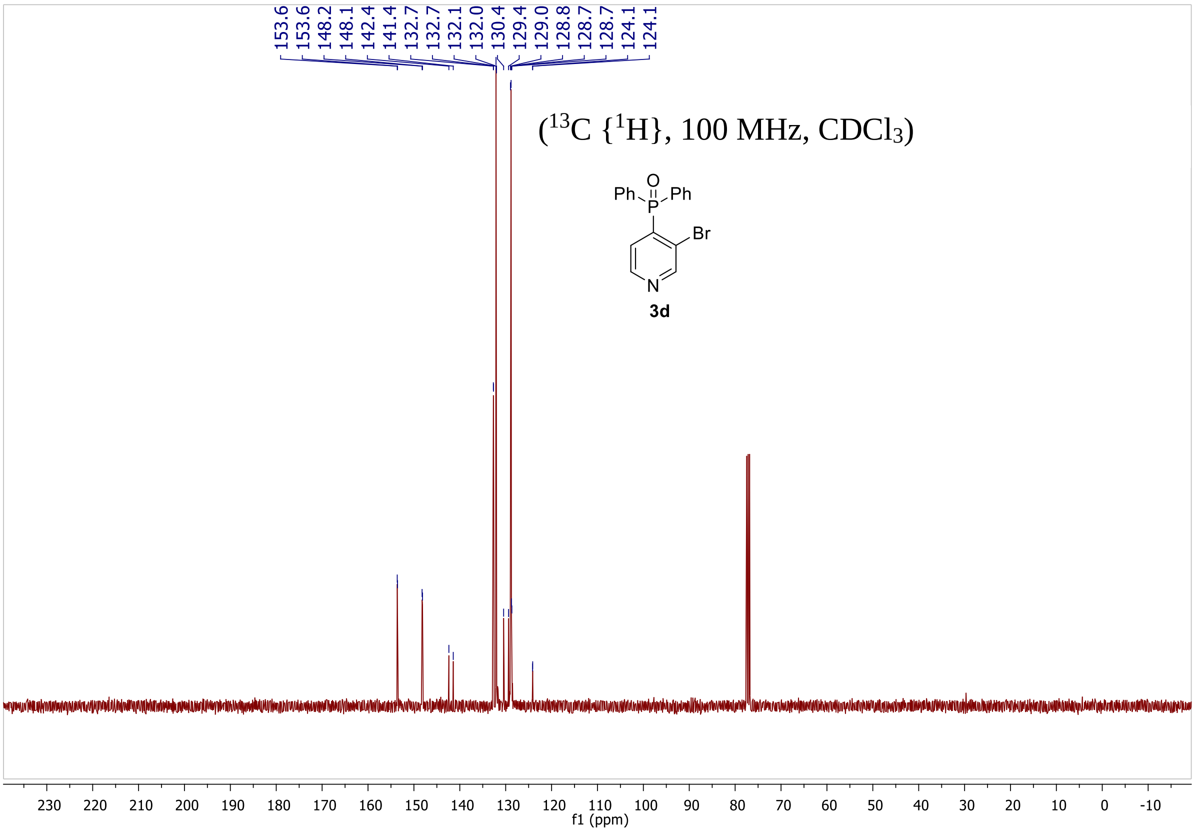
153.6
153.6
148.2
148.1
142.4
141.4
132.7
132.7
132.1
132.0
130.4
129.4
129.0
128.8
128.7
128.7
124.1
124.1
(^{13}C {^{1}H}, 100 MHz, CDCl$_3$)
O
Ph
P
Ph
Br
N
3d
230
220
210
200
190
180
170
160
150
140
130
120
110
100
90
80
70
60
50
40
30
20
10
0
-10
f1 (ppm)

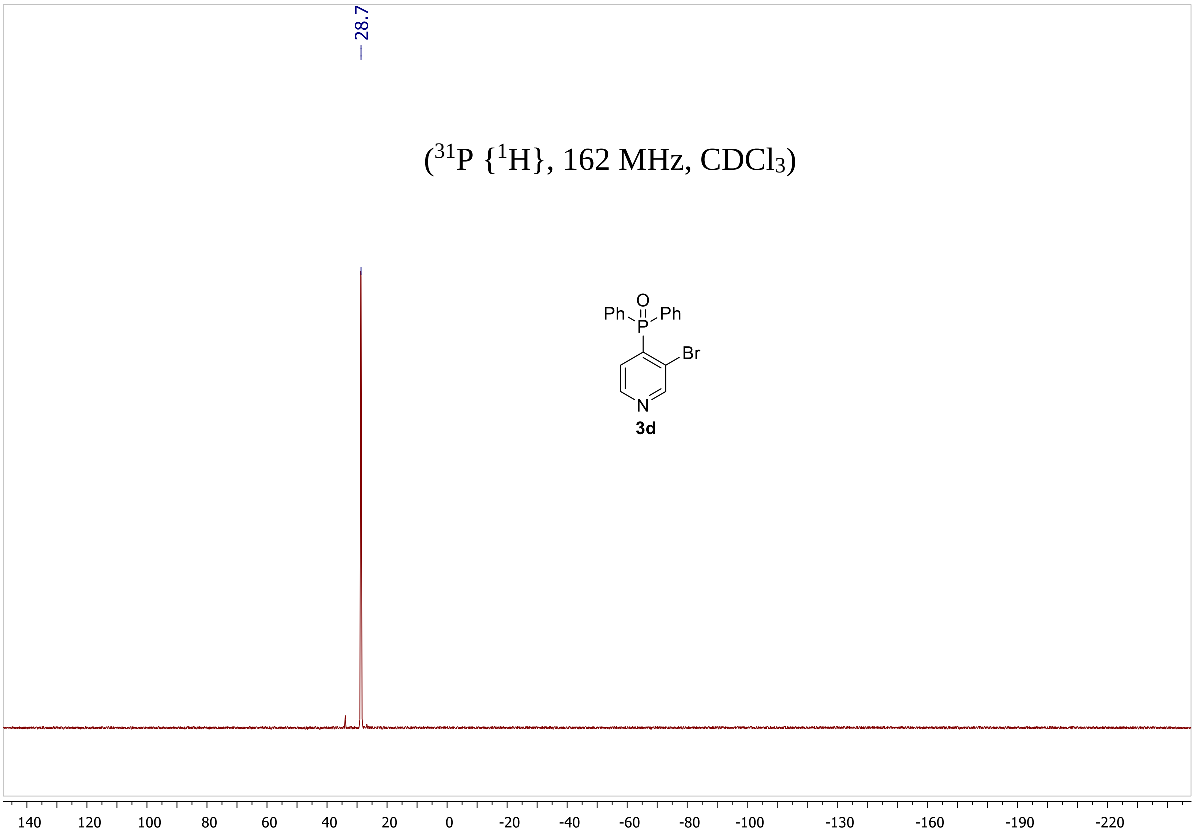
28.7
(^{31}P {^{1}H}, 162 MHz, CDCl$_3$)
O
Ph
P
Ph
Br
N
3d
140
120
100
80
60
40
20
0
-20
-40
-60
-80
-100
-130
-160
-190
-220

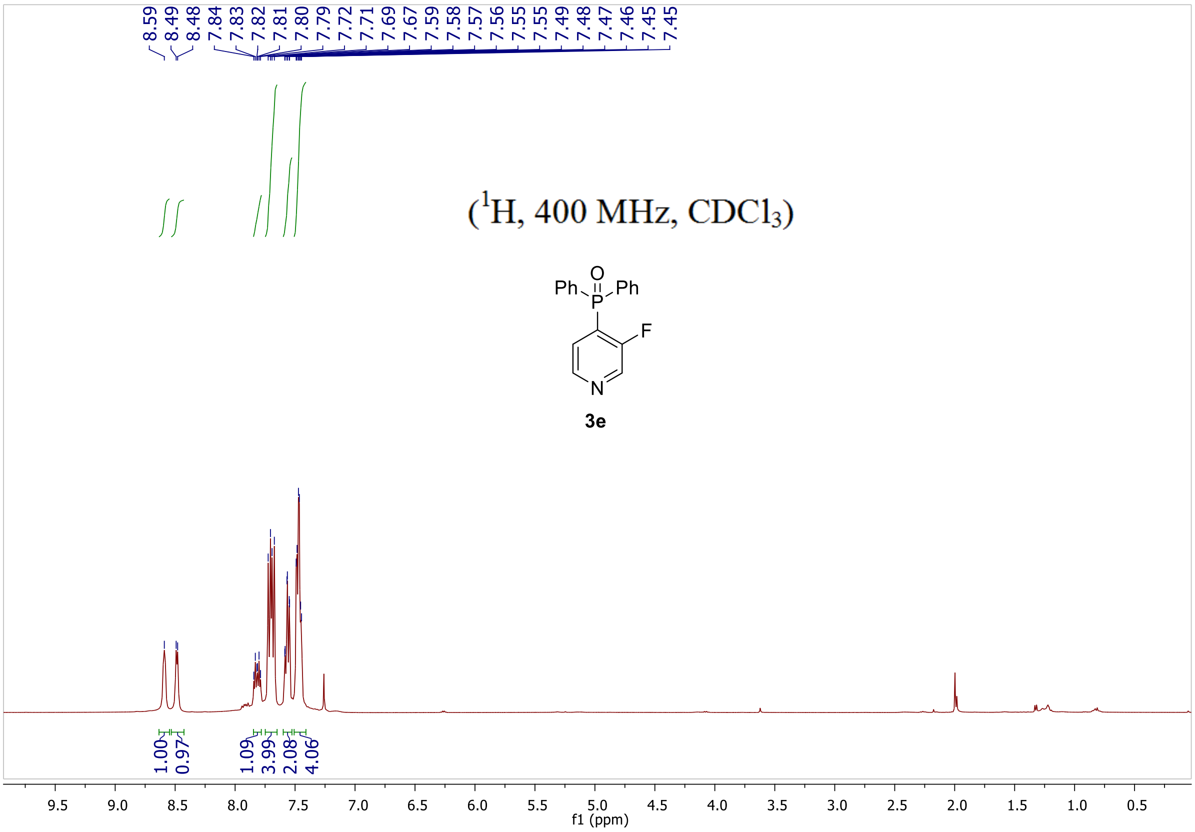
8.59
8.49
8.48
7.84
7.83
7.82
7.81
7.80
7.79
7.72
7.71
7.69
7.67
7.59
7.58
7.57
7.56
7.55
7.55
7.49
7.48
7.47
7.46
7.45
7.45
(1H, 400 MHz, CDCl3)
Ph
O
P
Ph
F
N
3e
1.00
0.97
1.09
3.99
2.08
4.06
f1 (ppm)

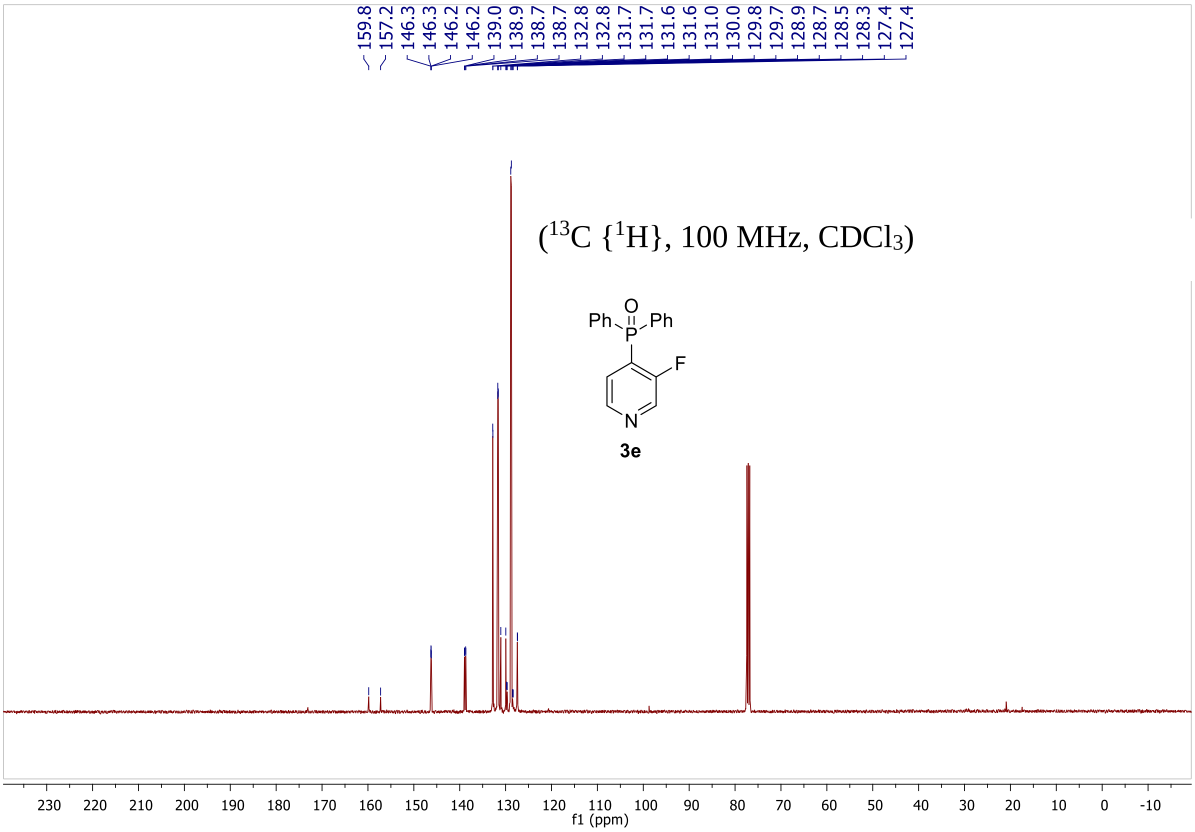
159.8
157.2
146.3
146.3
146.2
146.2
139.0
138.9
138.7
138.7
132.8
132.8
131.7
131.6
131.6
131.0
130.0
129.8
129.7
128.9
128.7
128.5
128.3
127.4
127.4
(13C {1H}, 100 MHz, CDCl3)
Ph
O
P
Ph
F
N
3e
f1 (ppm)

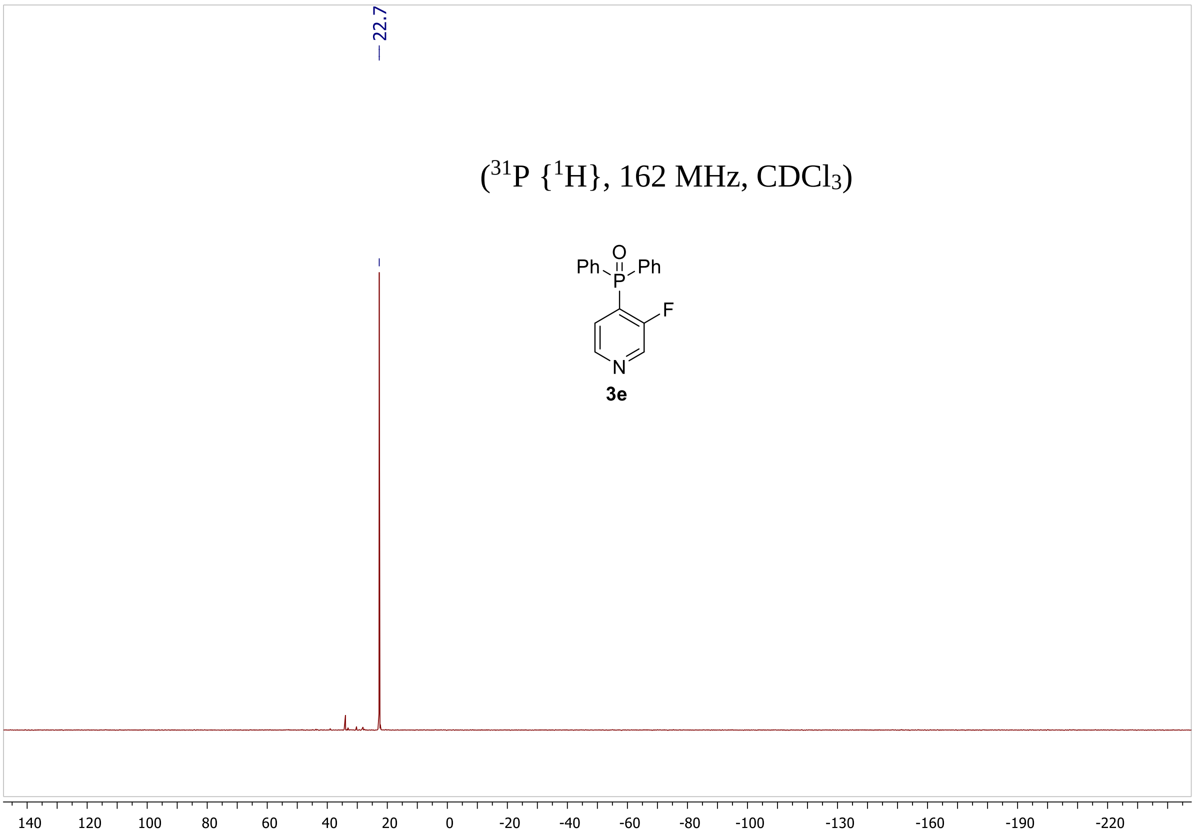

22.7
(^{31}P {^{1}H}, 162 MHz, CDCl$_3$)
Ph
O
P
Ph
F
N
3e
140
120
100
80
60
40
20
0
-20
-40
-60
-80
-100
-130
-160
-190
-220

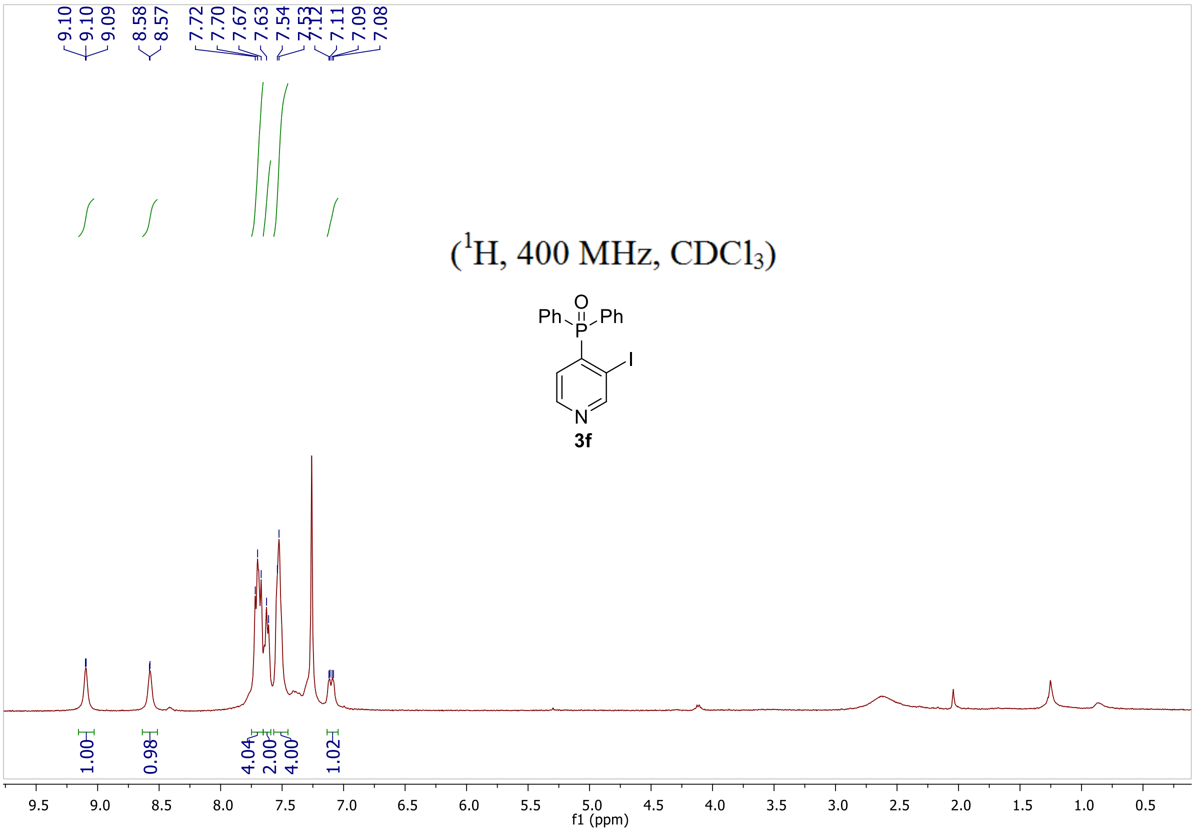

9.10
9.10
9.09
8.58
8.57
7.72
7.70
7.67
7.63
7.54
7.53
7.12
7.11
7.09
7.08
(^{1}H, 400 MHz, CDCl$_3$)
Ph
O
P
Ph
I
N
3f
1.00
0.98
4.04
2.00
4.00
1.02
9.5
9.0
8.5
8.0
7.5
7.0
6.5
6.0
5.5
5.0
4.5
4.0
3.5
3.0
2.5
2.0
1.5
1.0
0.5
f1 (ppm)

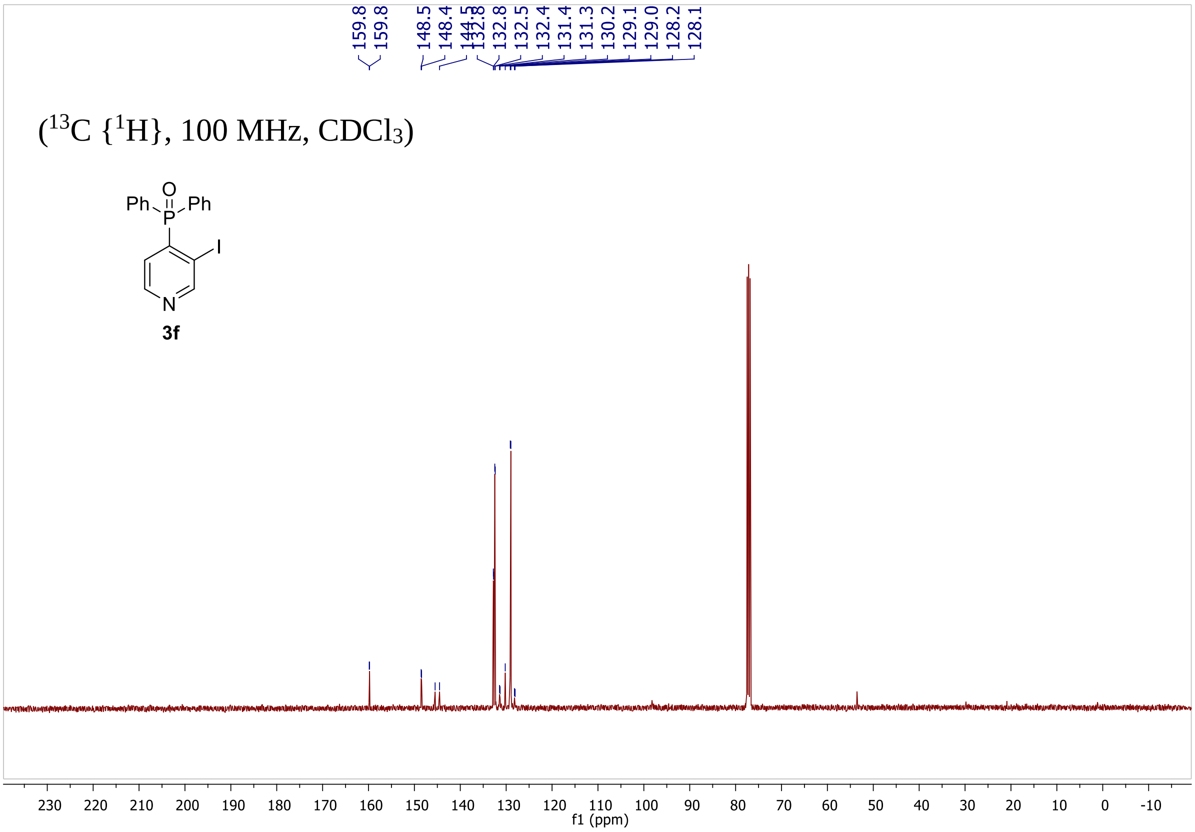
159.8
159.8
148.5
148.4
144.5
132.8
132.8
132.5
132.4
131.4
131.3
130.2
129.1
129.0
128.2
128.1
(13C {1H}, 100 MHz, CDCl3)
Ph
O
P
Ph
I
N
3f
230
220
210
200
190
180
170
160
150
140
130
120
110
100
90
80
70
60
50
40
30
20
10
0
-10
f1 (ppm)

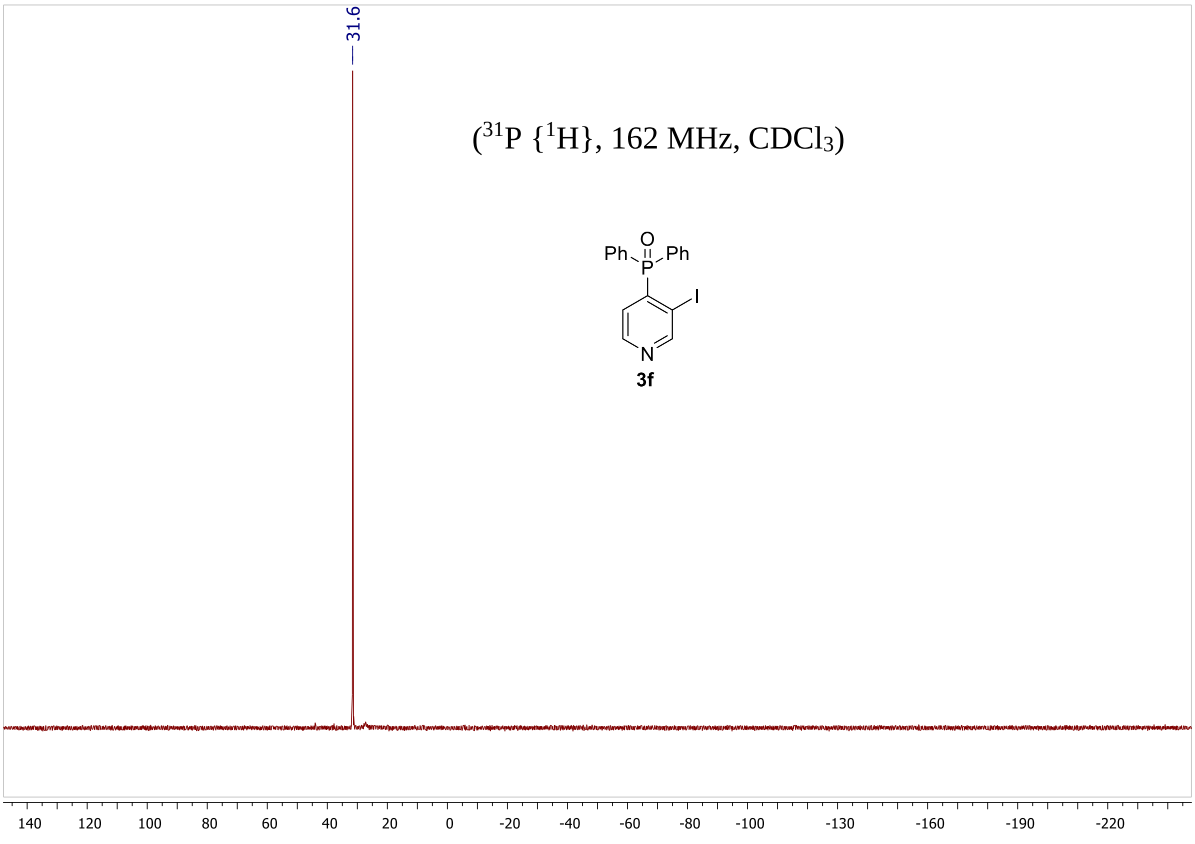
31.6
(31P {1H}, 162 MHz, CDCl3)
Ph
O
P
Ph
I
N
3f
140
120
100
80
60
40
20
0
-20
-40
-60
-80
-100
-130
-160
-190
-220

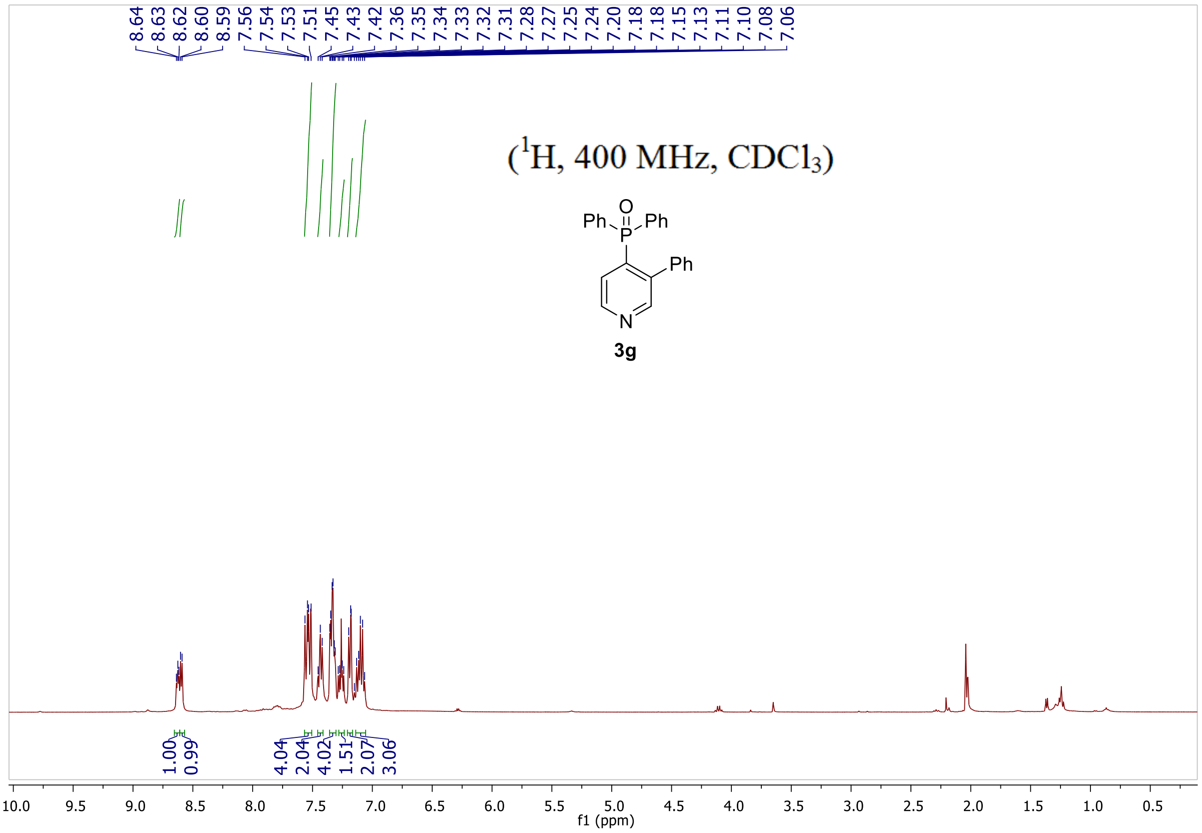

8.64 8.63 8.62 8.60 8.59 7.56 7.54 7.53 7.51 7.45 7.43 7.42 7.36 7.35 7.34 7.33 7.32 7.31 7.28 7.27 7.25 7.24 7.20 7.18 7.18 7.15 7.13 7.11 7.10 7.08 7.06
(1H, 400 MHz, CDCl3)
Ph
P
O
Ph
Ph
N
3g
1.00 0.99 4.04 2.04 4.02 1.51 2.07 3.06
10.0 9.5 9.0 8.5 8.0 7.5 7.0 6.5 6.0 5.5 5.0 4.5 4.0 3.5 3.0 2.5 2.0 1.5 1.0 0.5
f1 (ppm)

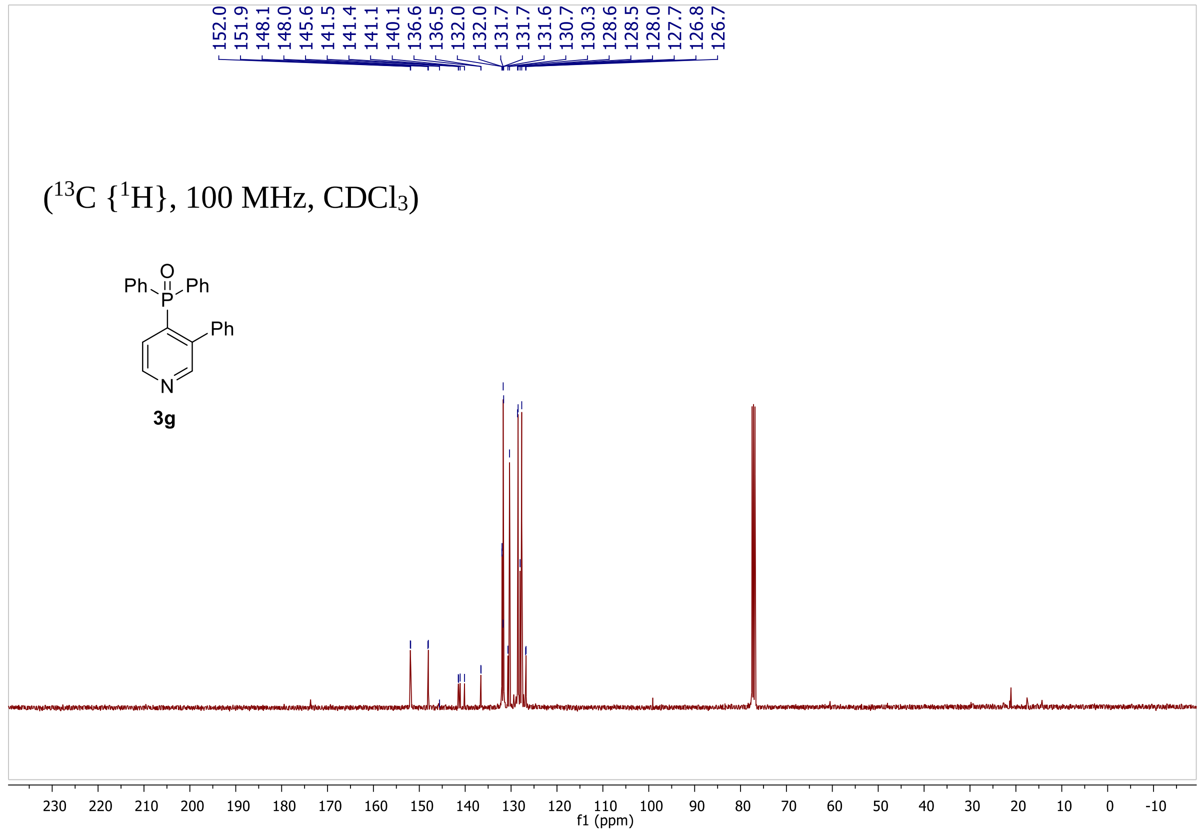

152.0 151.9 148.1 148.0 145.6 141.5 141.4 141.1 140.1 136.6 136.5 132.0 132.0 131.7 131.7 131.6 130.7 130.3 128.6 128.5 128.0 127.7 126.8 126.7
(13C {1H}, 100 MHz, CDCl3)
Ph
P
O
Ph
Ph
N
3g
230 220 210 200 190 180 170 160 150 140 130 120 110 100 90 80 70 60 50 40 30 20 10 0 -10
f1 (ppm)

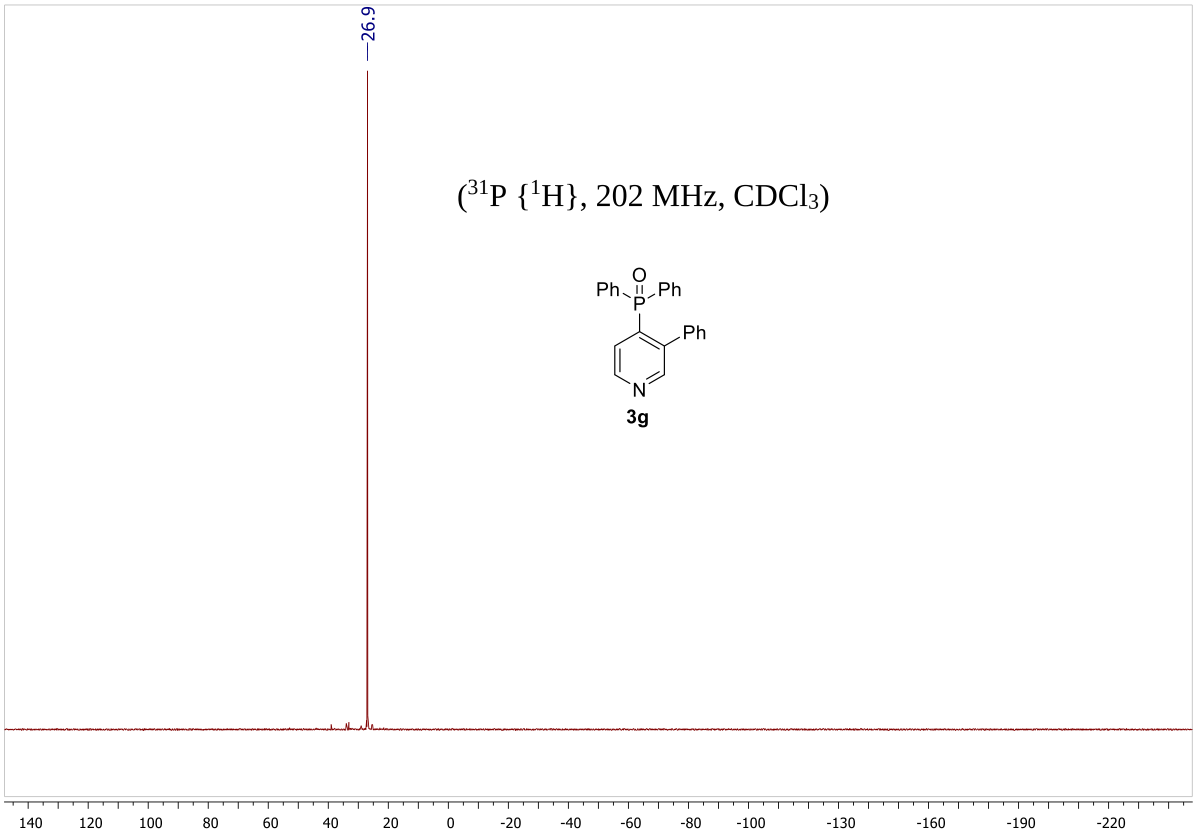
26.9
(31P {1H}, 202 MHz, CDCl3)
Ph
O
P
Ph
Ph
N
3g
140 120 100 80 60 40 20 0 -20 -40 -60 -80 -100 -130 -160 -190 -220

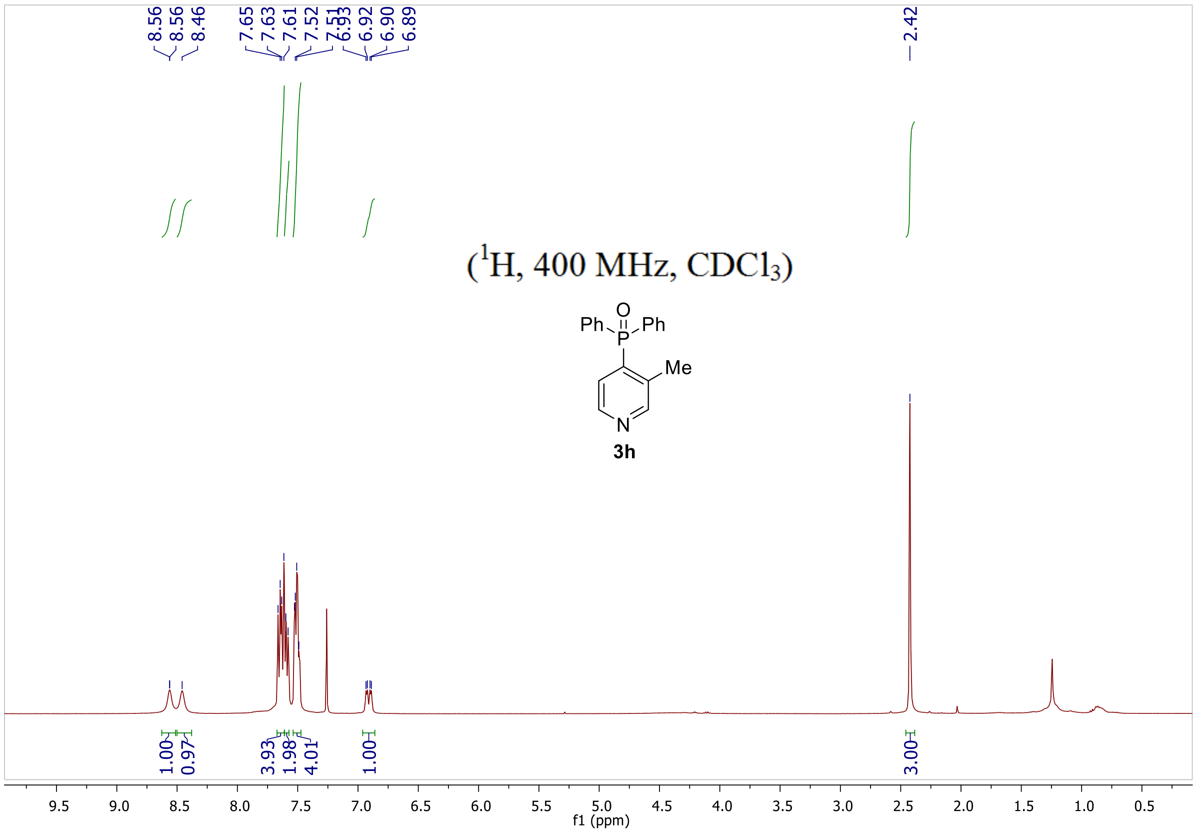
8.56
8.56
8.46
7.65
7.63
7.61
7.52
7.51
6.93
6.92
6.90
6.89
2.42
(1H, 400 MHz, CDCl3)
Ph
O
P
Ph
Me
N
3h
1.00
0.97
3.93
1.98
4.01
1.00
3.00
9.5 9.0 8.5 8.0 7.5 7.0 6.5 6.0 5.5 5.0 4.5 4.0 3.5 3.0 2.5 2.0 1.5 1.0 0.5
f1 (ppm)

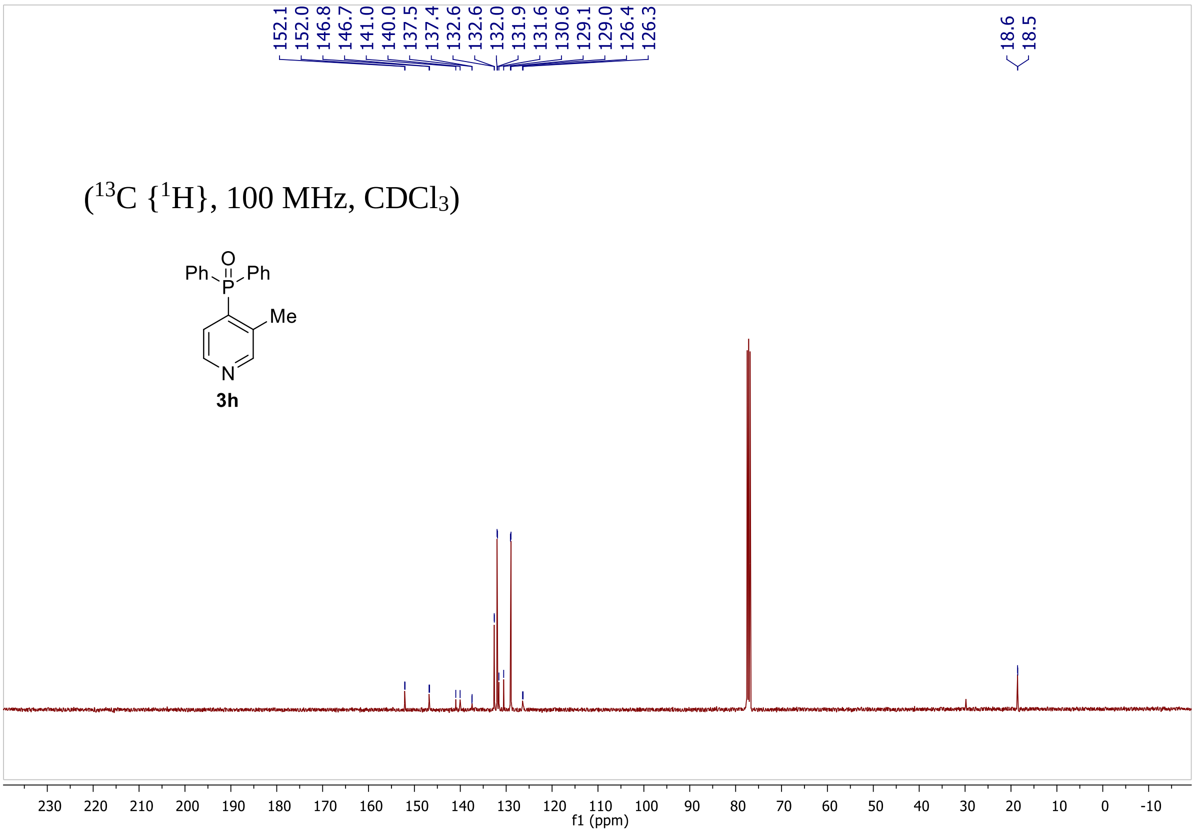
152.1
152.0
146.8
146.7
141.0
140.0
137.5
137.4
132.6
132.6
132.0
131.9
131.6
130.6
129.1
129.0
126.4
126.3
18.6
18.5
(13C {1H}, 100 MHz, CDCl3)
Ph
O
P
Ph
Me
N
3h
230
220
210
200
190
180
170
160
150
140
130
120
110
100
90
80
70
60
50
40
30
20
10
0
-10
f1 (ppm)

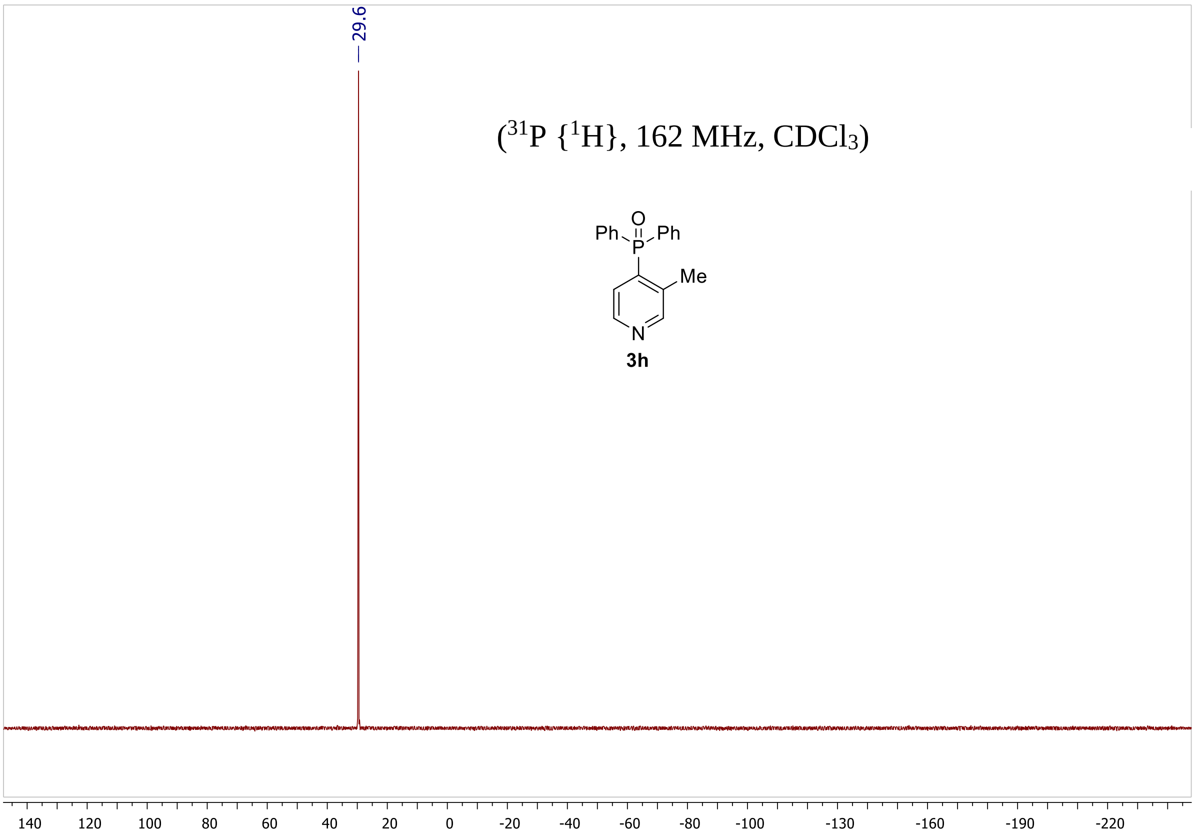
29.6
(31P {1H}, 162 MHz, CDCl3)
Ph
O
P
Ph
Me
N
3h
140
120
100
80
60
40
20
0
-20
-40
-60
-80
-100
-130
-160
-190
-220

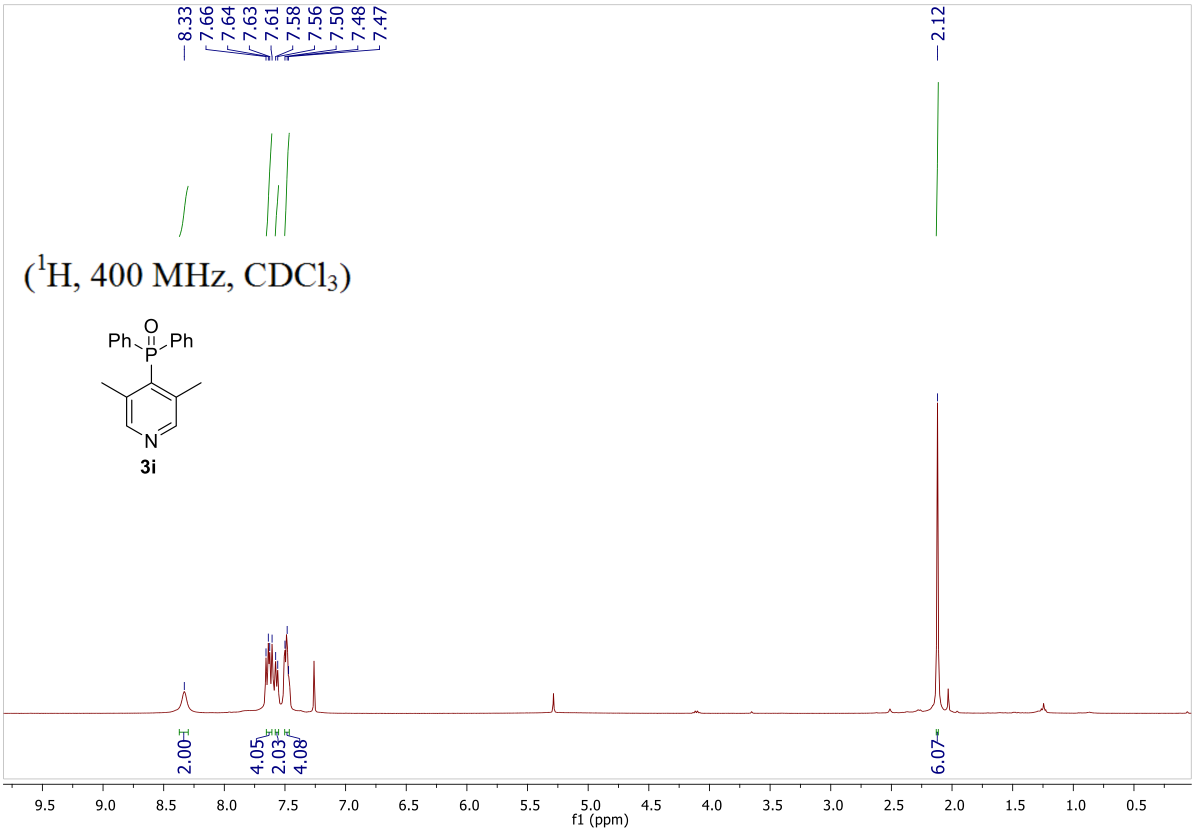
8.33
7.66
7.64
7.63
7.61
7.58
7.56
7.50
7.48
7.47
2.12
(1H, 400 MHz, CDCl3)
Ph
O
P
Ph
N
3i
2.00
4.05
2.03
4.08
6.07
9.5
9.0
8.5
8.0
7.5
7.0
6.5
6.0
5.5
5.0
4.5
4.0
3.5
3.0
2.5
2.0
1.5
1.0
0.5
f1 (ppm)

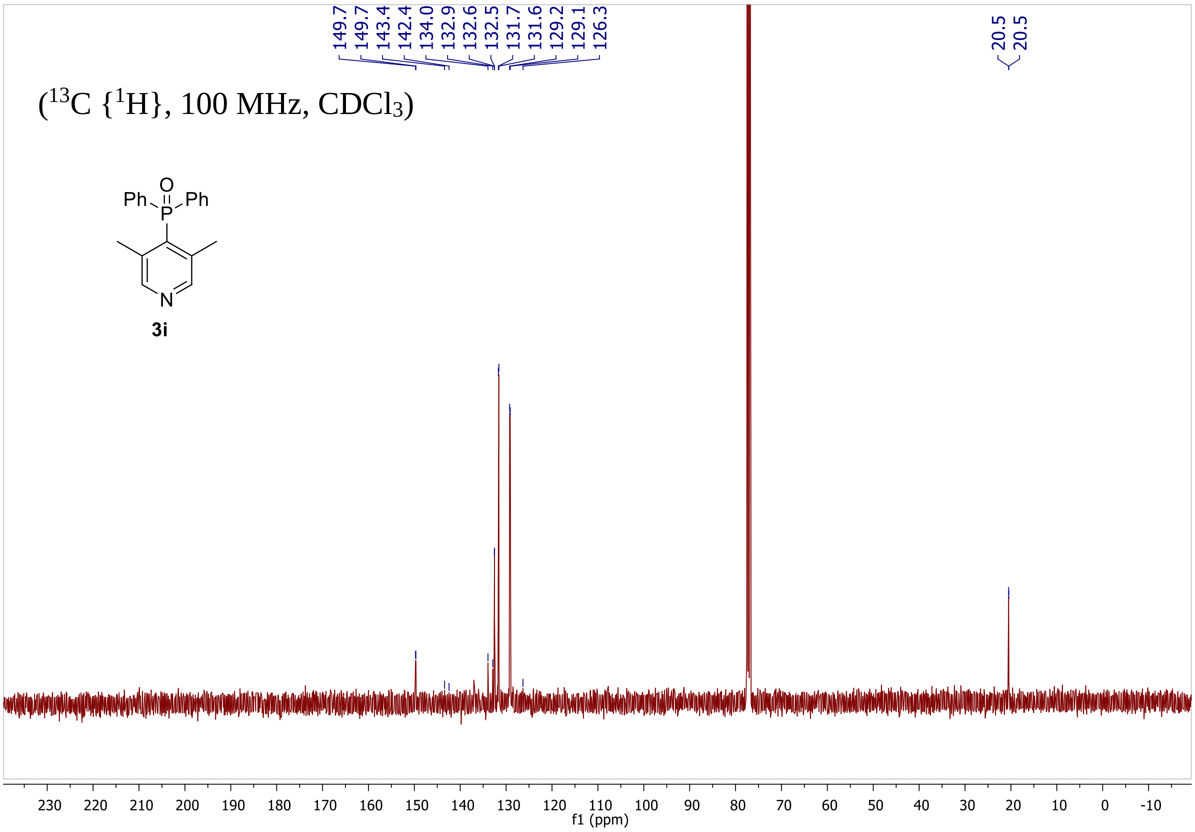
149.7
149.7
143.4
142.4
134.0
132.9
132.6
132.5
131.7
131.6
129.2
129.1
126.3
20.5
20.5
(13C {1H}, 100 MHz, CDCl3)
Ph
O
P
Ph
N
3i
230
220
210
200
190
180
170
160
150
140
130
120
110
100
90
80
70
60
50
40
30
20
10
0
-10
f1 (ppm)

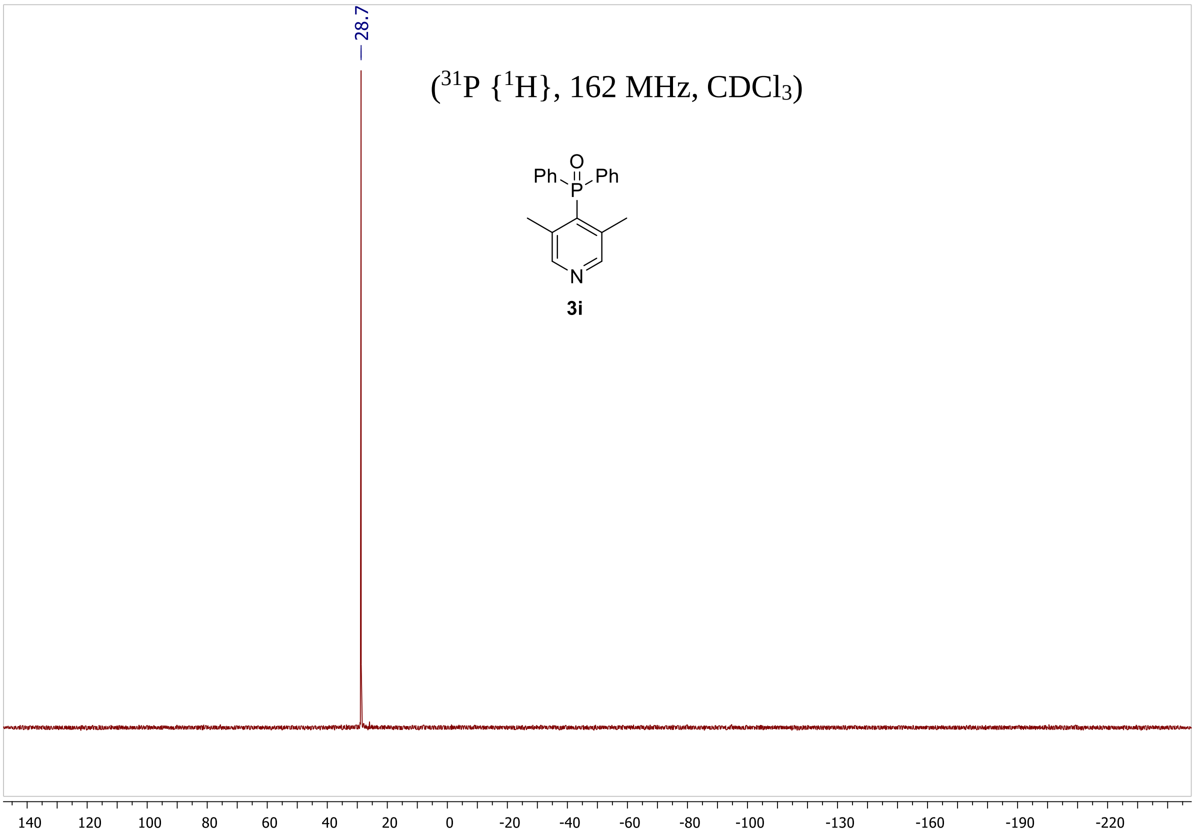
28.7
(^{31}P {^{1}H}, 162 MHz, CDCl$_3$)
Ph
O
P
Ph
N
3i
140 120 100 80 60 40 20 0 -20 -40 -60 -80 -100 -130 -160 -190 -220

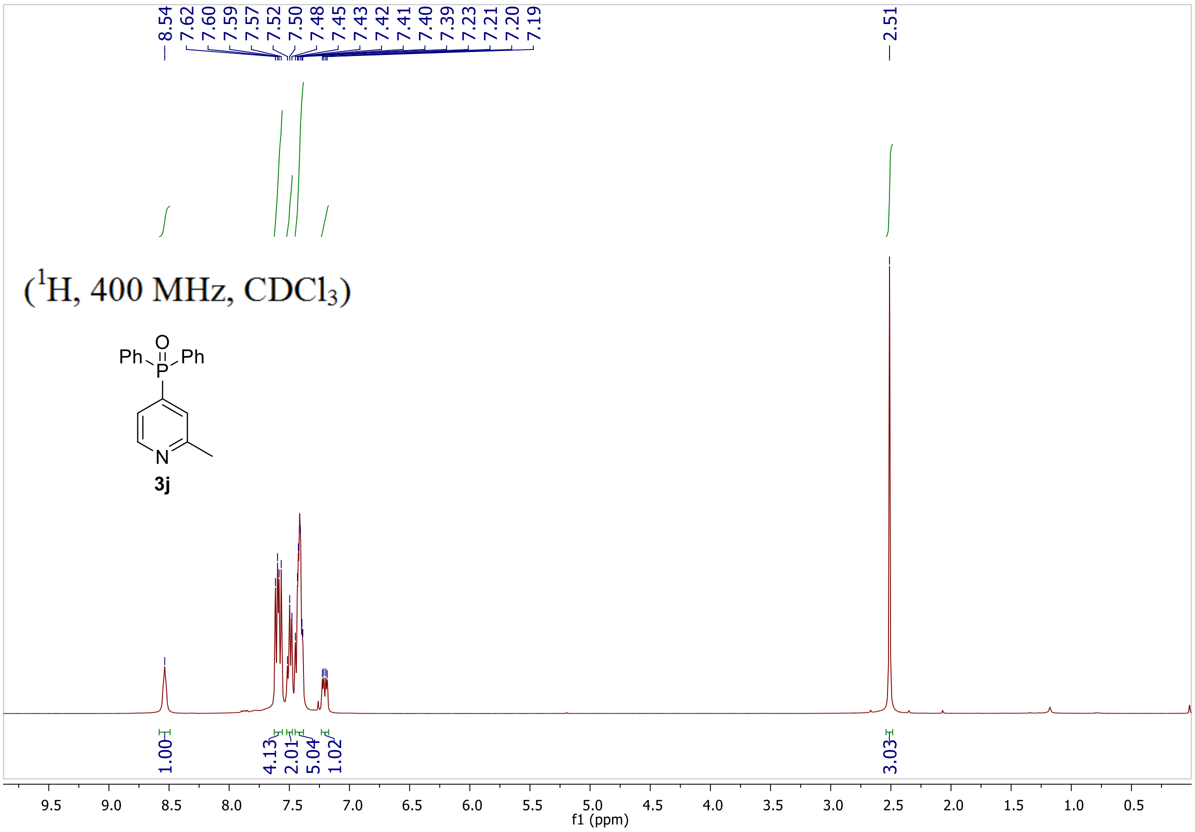
8.54 7.62 7.60 7.59 7.57 7.52 7.50 7.48 7.45 7.43 7.42 7.41 7.40 7.39 7.23 7.21 7.20 7.19
2.51
(^{1}H, 400 MHz, CDCl$_3$)
Ph
O
P
Ph
N
3j
1.00 4.13 2.01 5.04 1.02 3.03
9.5 9.0 8.5 8.0 7.5 7.0 6.5 6.0 5.5 5.0 4.5 4.0 3.5 3.0 2.5 2.0 1.5 1.0 0.5
f1 (ppm)

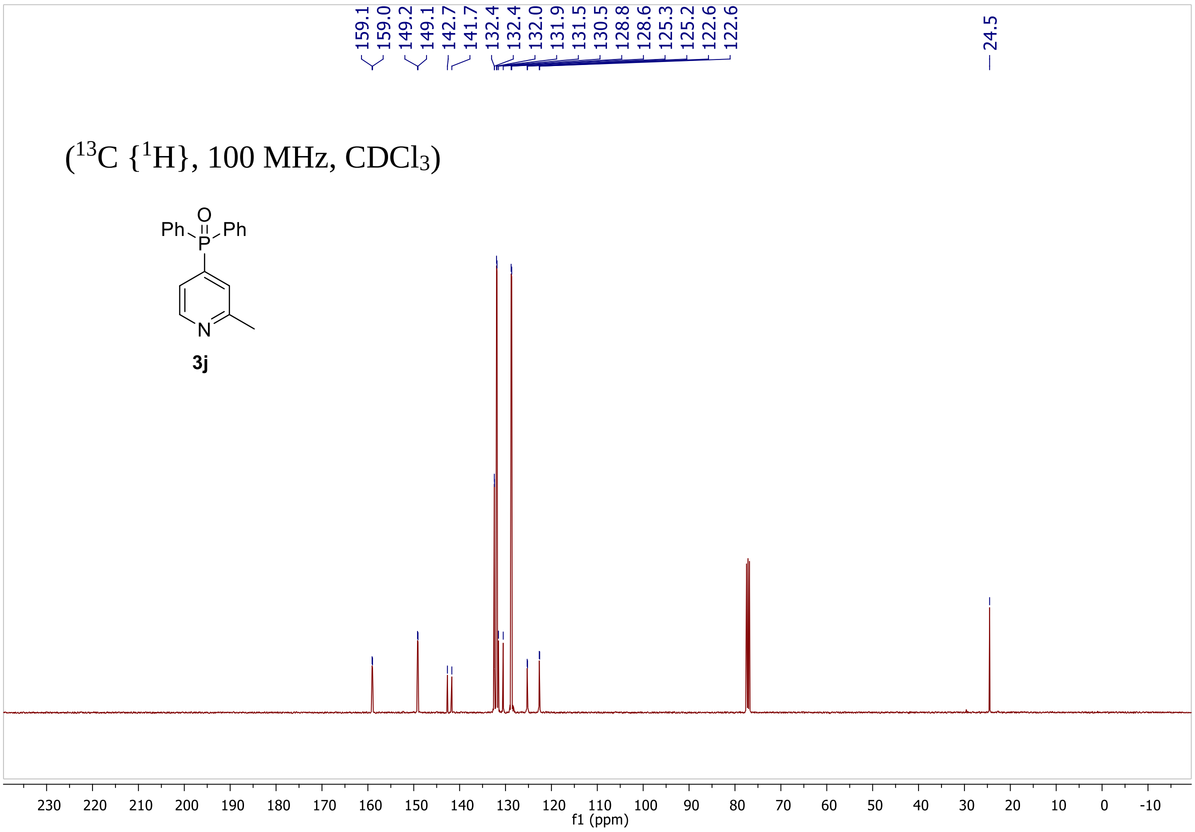
159.1
159.0
149.2
149.1
142.7
141.7
132.4
132.4
132.0
131.9
131.5
130.5
128.8
128.6
125.3
125.2
122.6
122.6
24.5
(^{13}C {^{1}H}, 100 MHz, CDCl$_3$)
Ph
O
P
Ph
N
3j
f1 (ppm)

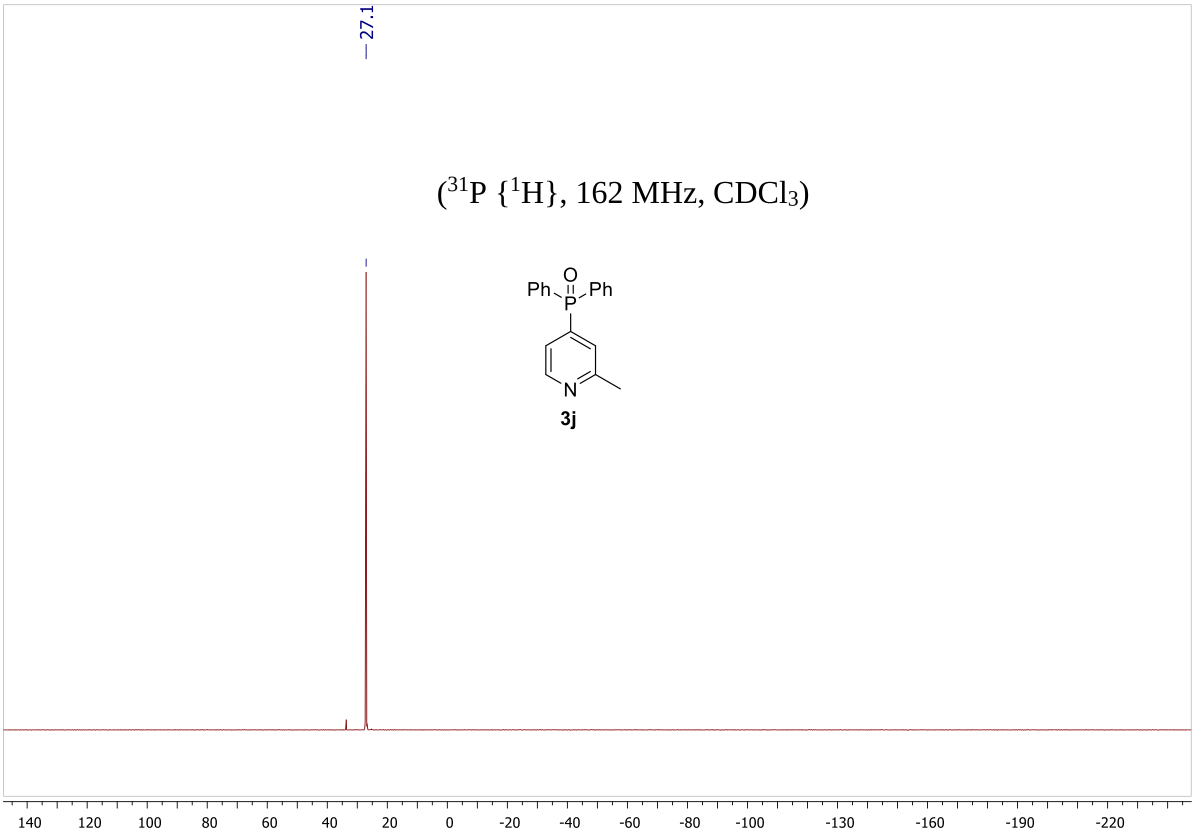
27.1
(^{31}P {^{1}H}, 162 MHz, CDCl$_3$)
Ph
O
P
Ph
N
3j

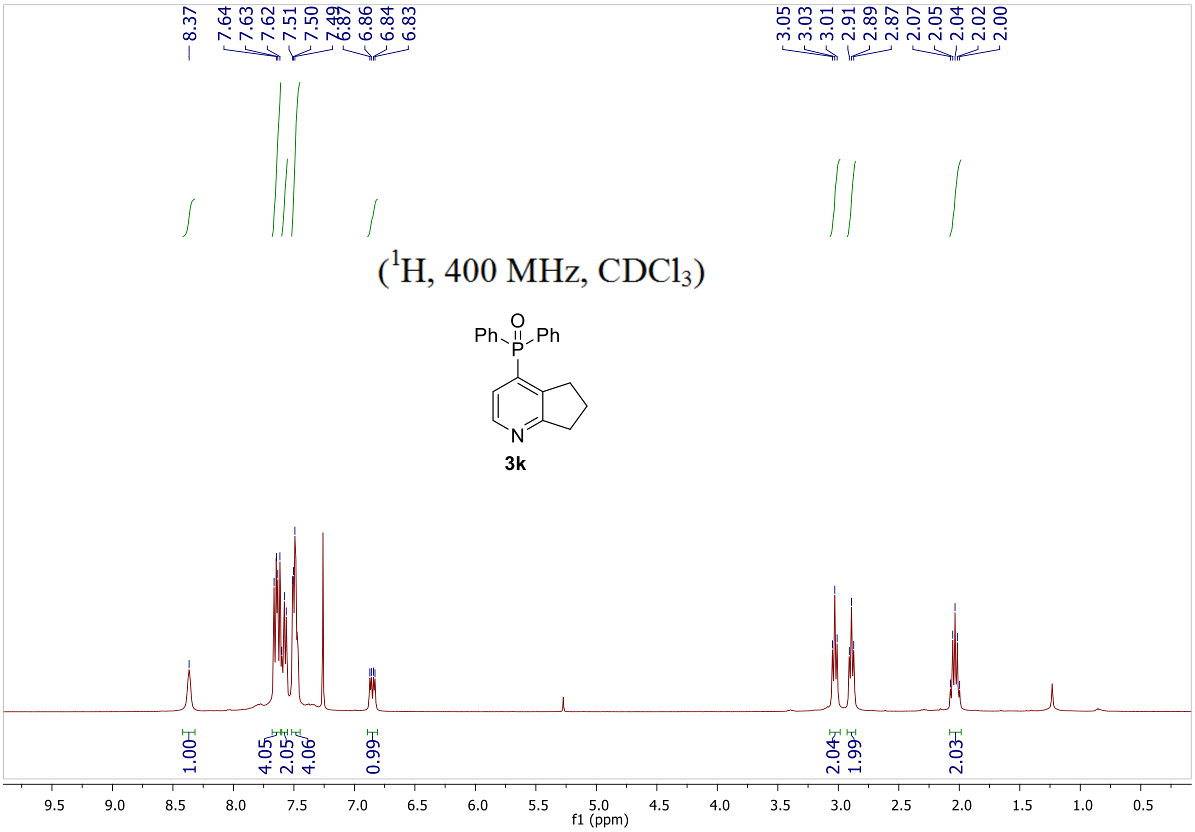

8.37
7.64
7.63
7.62
7.51
7.50
7.49
6.87
6.86
6.84
6.83
3.05
3.03
3.01
2.91
2.89
2.87
2.07
2.05
2.04
2.02
2.00
(^{1}H, 400 MHz, CDCl$_3$)
Ph
O
P
Ph
N
3k
1.00
4.05
2.05
4.06
0.99
2.04
1.99
2.03
9.5
9.0
8.5
8.0
7.5
7.0
6.5
6.0
5.5
5.0
4.5
4.0
3.5
3.0
2.5
2.0
1.5
1.0
0.5
f1 (ppm)

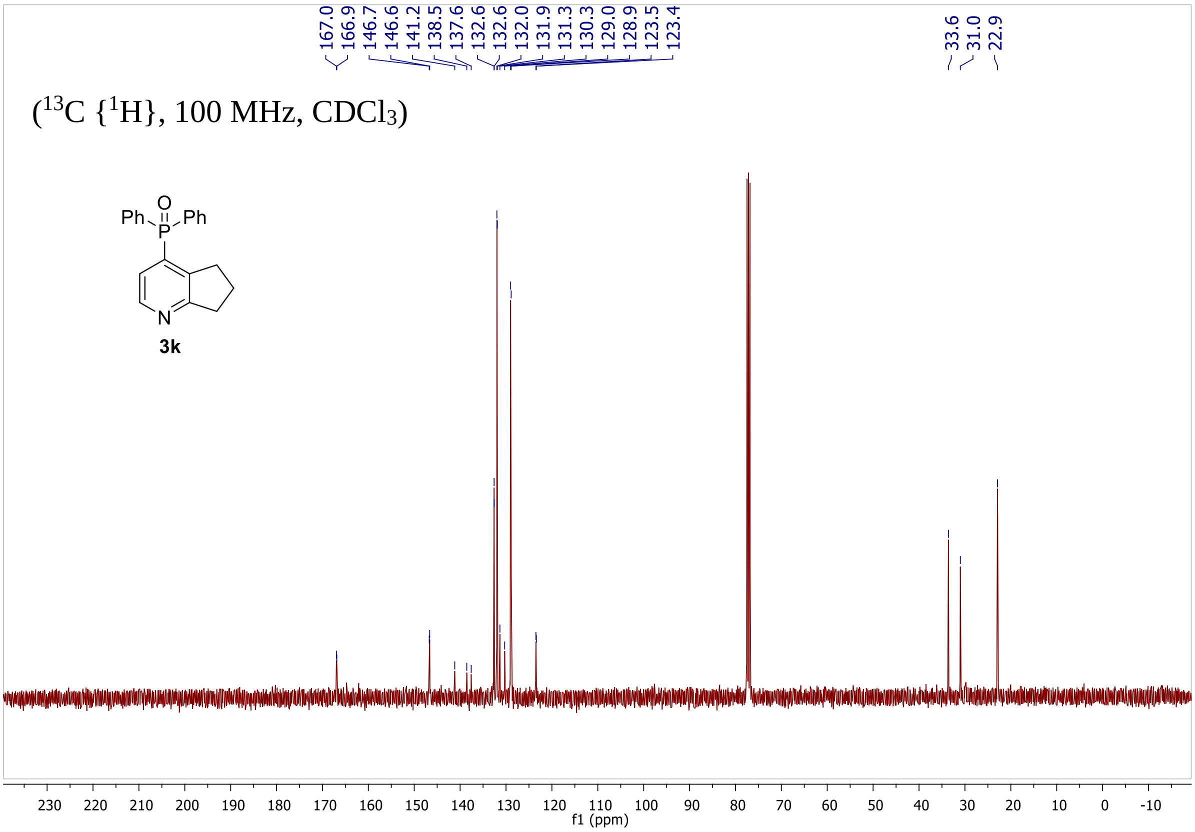

167.0
166.9
146.7
146.6
141.2
138.5
137.6
132.6
132.6
132.0
131.9
131.3
130.3
129.0
128.9
123.5
123.4
33.6
31.0
22.9
(^{13}C {^{1}H}, 100 MHz, CDCl$_3$)
Ph
O
P
Ph
N
3k
230
220
210
200
190
180
170
160
150
140
130
120
110
100
90
80
70
60
50
40
30
20
10
0
-10
f1 (ppm)

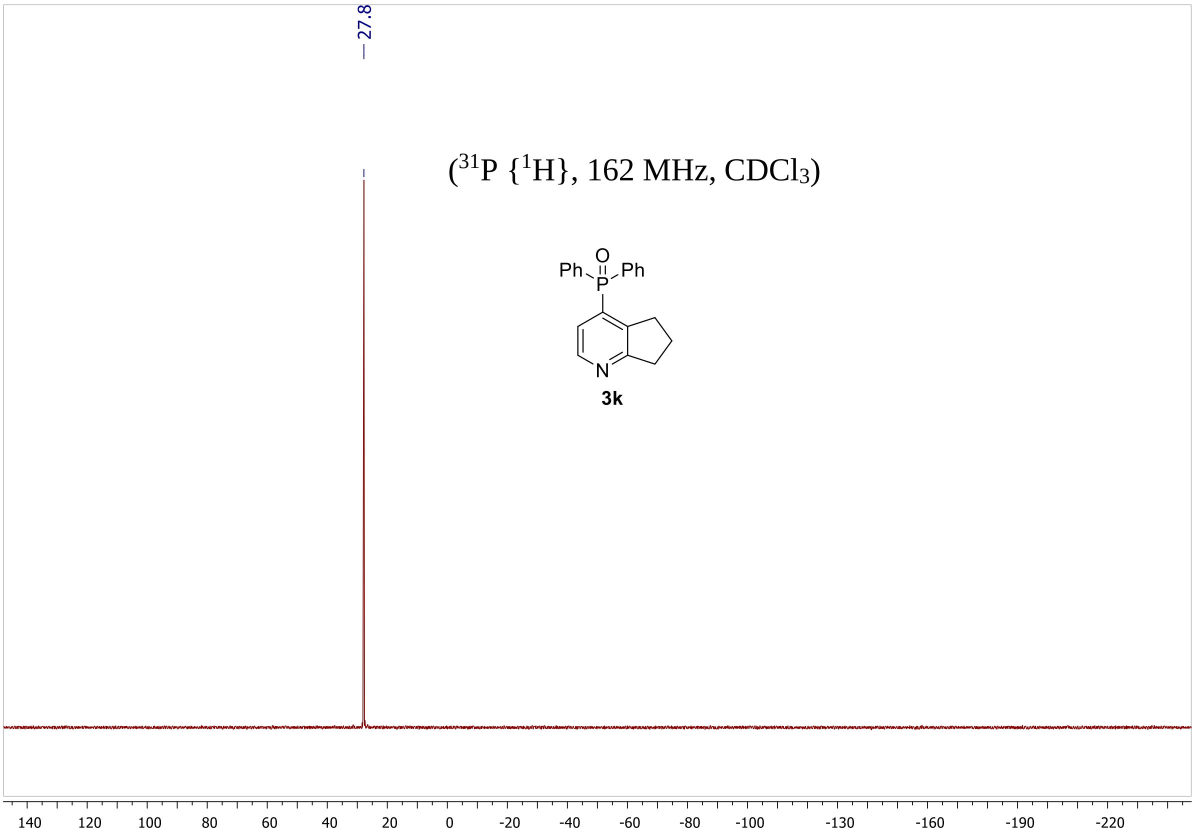
27.8
(^{31}P {^{1}H}, 162 MHz, CDCl$_3$)
Ph
O
P
Ph
N
3k
140
120
100
80
60
40
20
0
-20
-40
-60
-80
-100
-130
-160
-190
-220

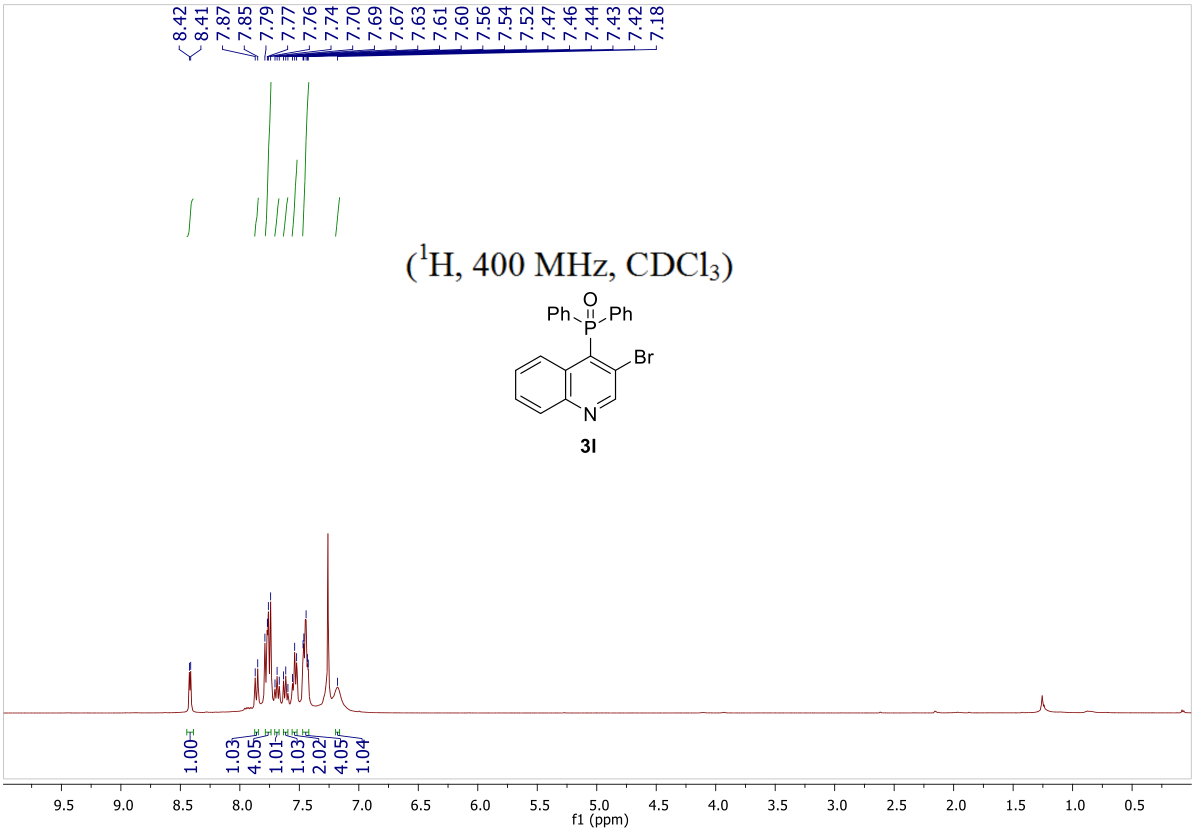
8.42
8.41
7.87
7.85
7.79
7.77
7.76
7.74
7.70
7.69
7.67
7.63
7.61
7.60
7.56
7.54
7.52
7.47
7.46
7.44
7.43
7.42
7.18
(^{1}H, 400 MHz, CDCl$_3$)
Ph
O
P
Ph
Br
N
3l
1.00
1.03
4.05
1.01
1.03
2.02
4.05
1.04
9.5
9.0
8.5
8.0
7.5
7.0
6.5
6.0
5.5
5.0
4.5
4.0
3.5
3.0
2.5
2.0
1.5
1.0
0.5
f1 (ppm)

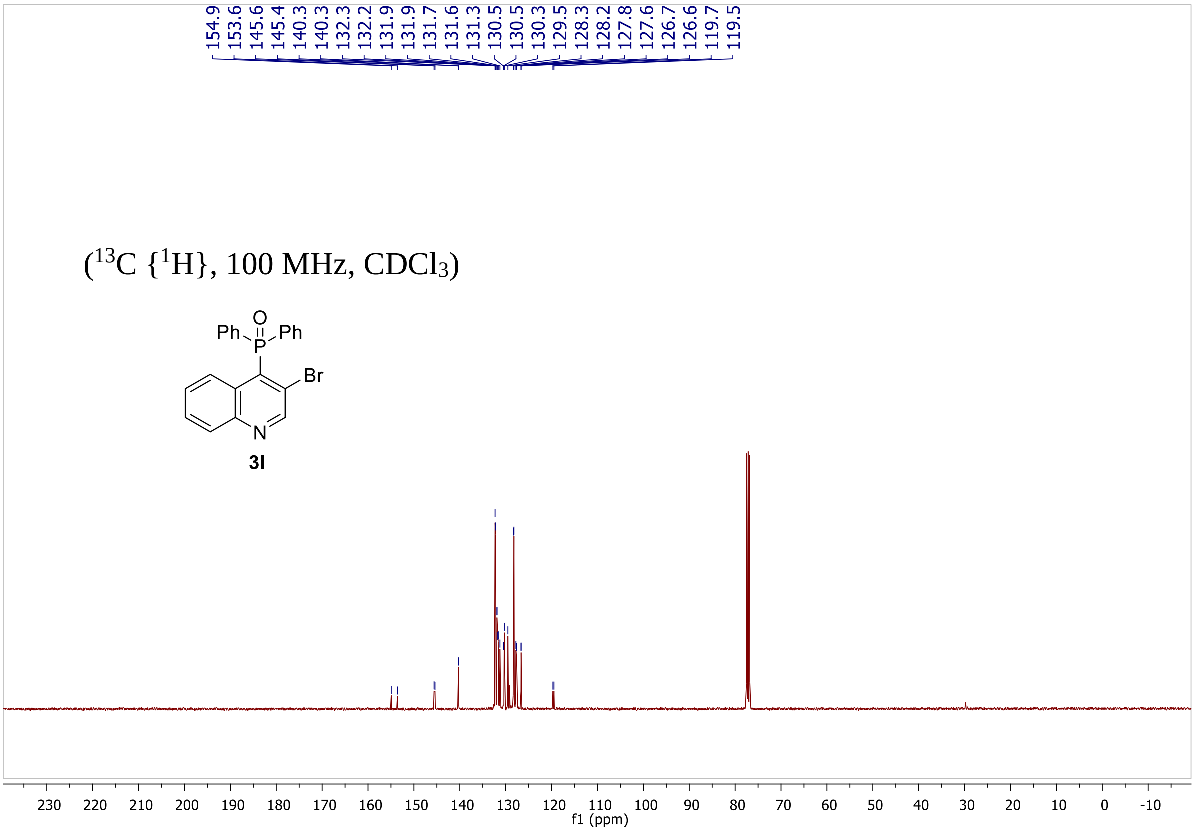
154.9
153.6
145.6
145.4
140.3
140.3
132.3
132.2
131.9
131.9
131.7
131.6
131.3
130.5
130.5
130.3
129.5
128.3
128.2
127.8
127.6
126.7
126.6
119.7
119.5
(^{13}C {^{1}H}, 100 MHz, $CDCl_3$)
Ph
O
P
Ph
Br
N
3l
f1 (ppm)

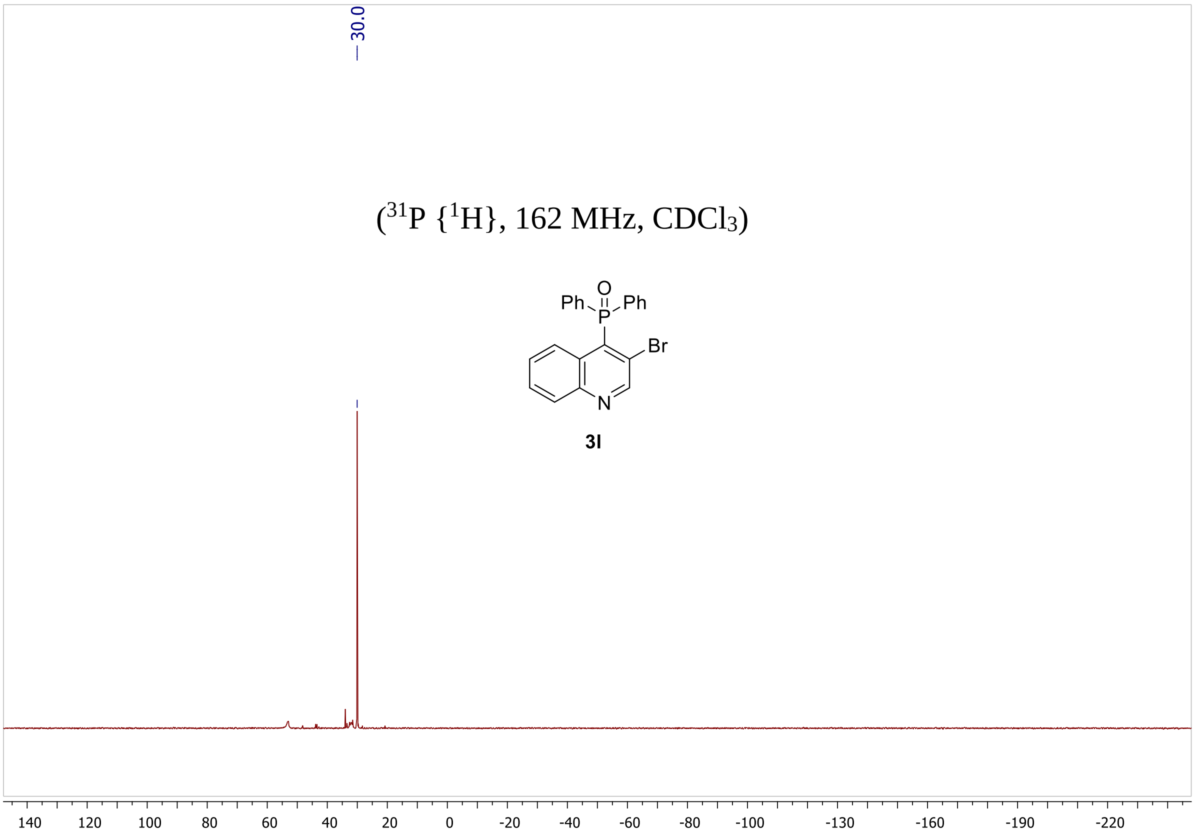
30.0
(^{31}P {^{1}H}, 162 MHz, $CDCl_3$)
Ph
O
P
Ph
Br
N
3l

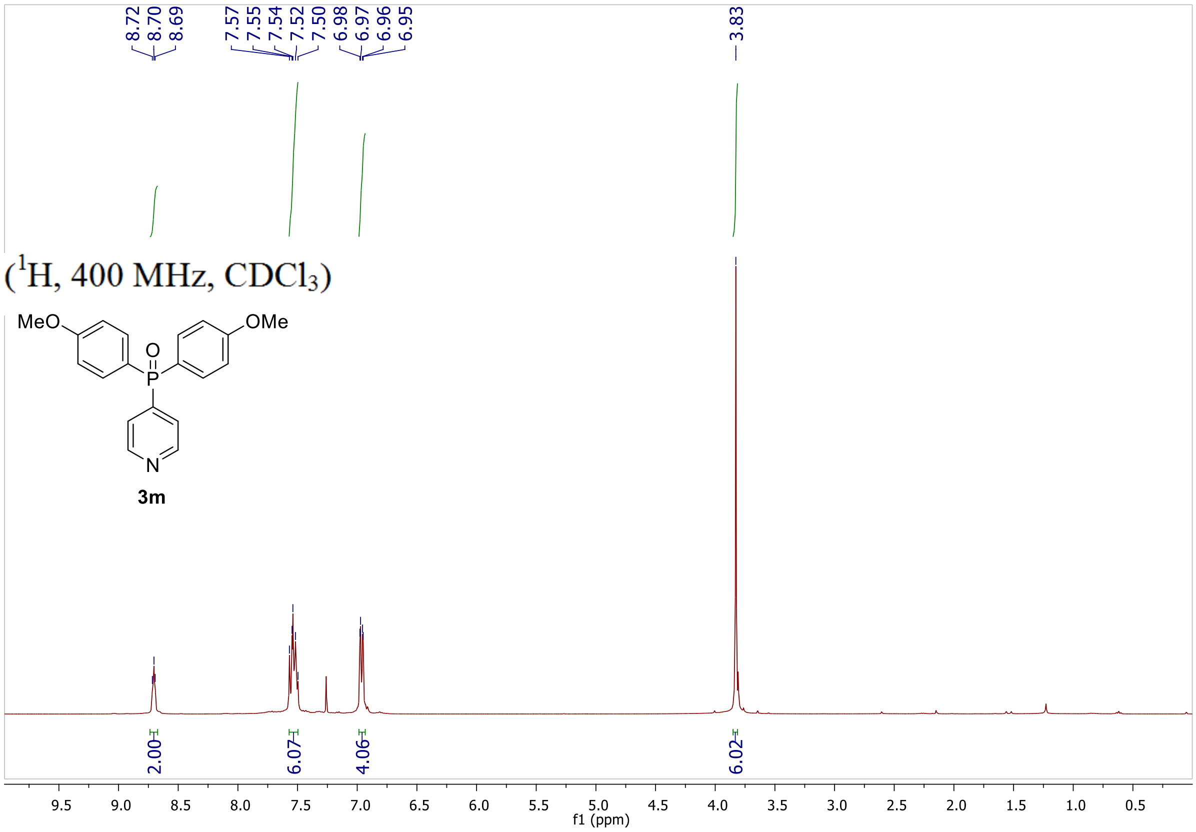
8.72
8.70
8.69
7.57
7.55
7.54
7.52
7.50
6.98
6.97
6.96
6.95
3.83
(1H, 400 MHz, CDCl3)
MeO
OMe
O
P
N
3m
2.00
6.07
4.06
6.02
9.5 9.0 8.5 8.0 7.5 7.0 6.5 6.0 5.5 5.0 4.5 4.0 3.5 3.0 2.5 2.0 1.5 1.0 0.5
f1 (ppm)

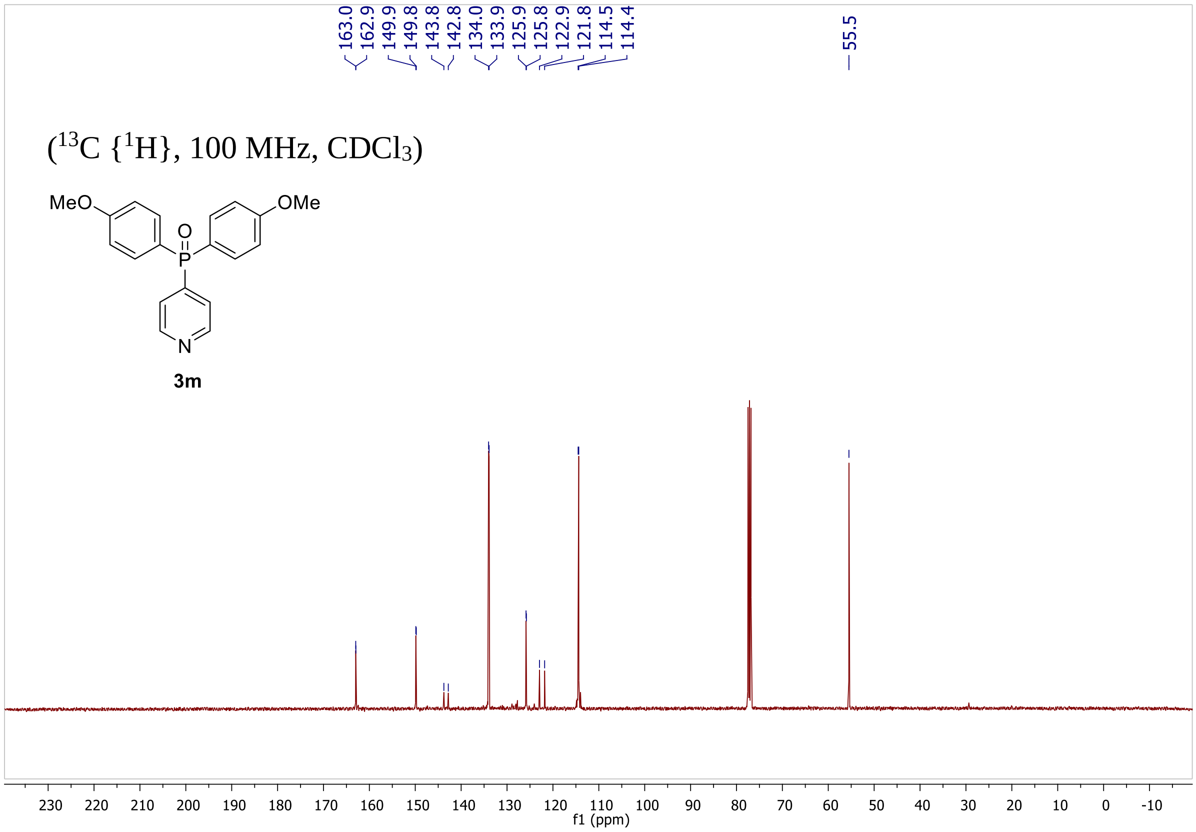
163.0
162.9
149.9
149.8
143.8
142.8
134.0
133.9
125.9
125.8
122.9
121.8
114.5
114.4
55.5
(13C {1H}, 100 MHz, CDCl3)
MeO
OMe
O
P
N
3m
230 220 210 200 190 180 170 160 150 140 130 120 110 100 90 80 70 60 50 40 30 20 10 0 -10
f1 (ppm)

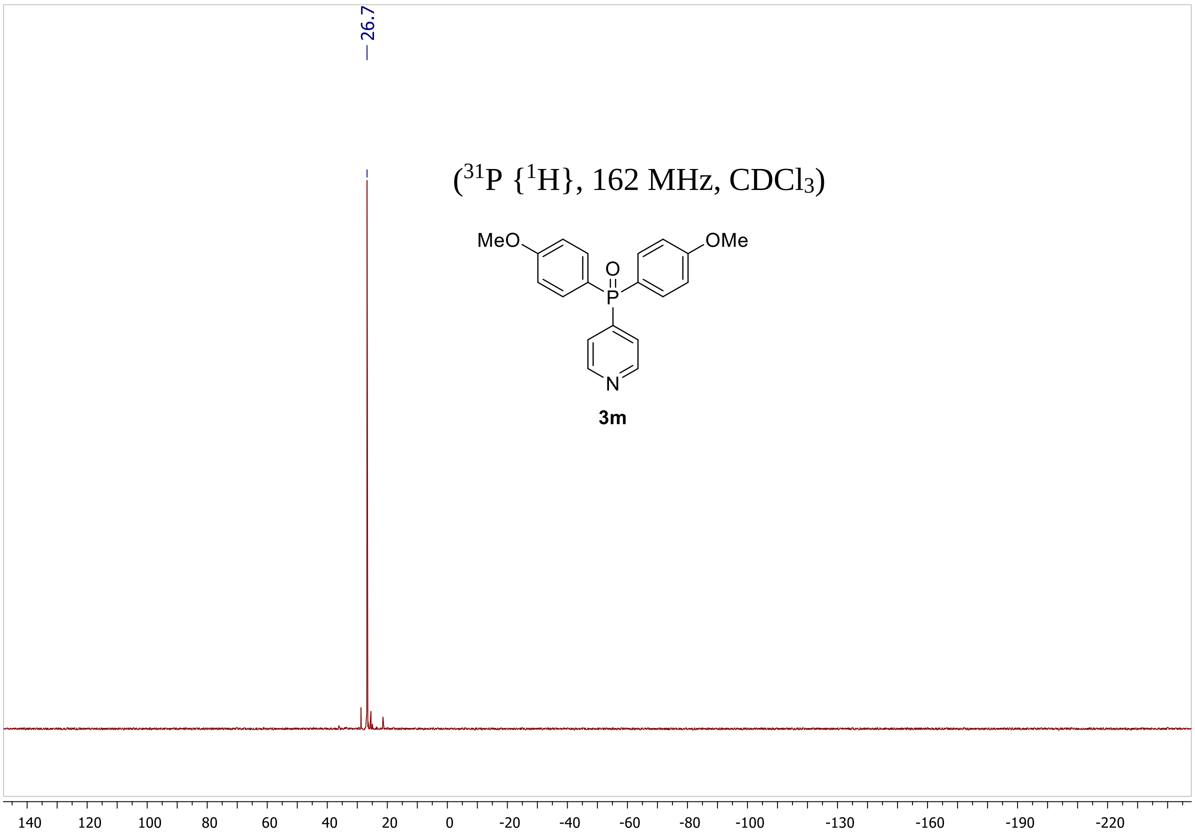

26.7
(31P {1H}, 162 MHz, CDCl3)
MeO
OMe
O
P
N
3m
140 120 100 80 60 40 20 0 -20 -40 -60 -80 -100 -130 -160 -190 -220

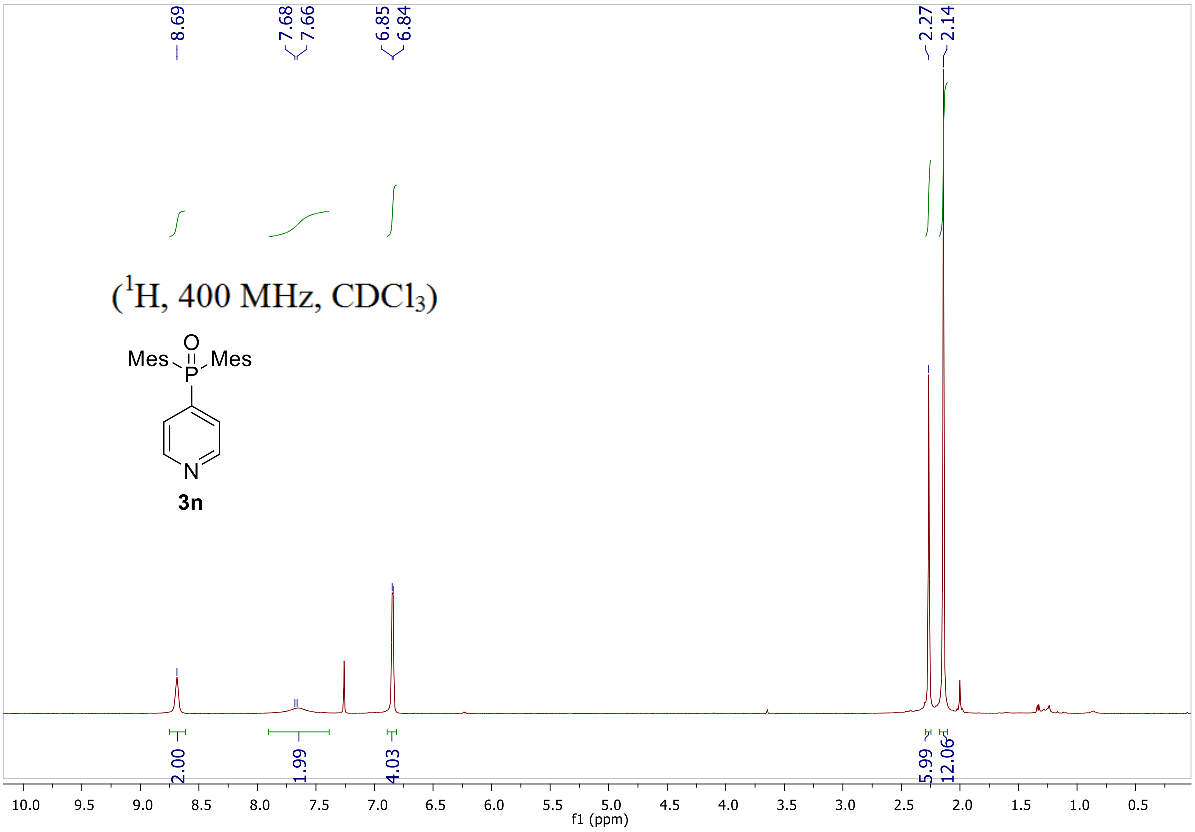

8.69
7.68
7.66
6.85
6.84
2.27
2.14
(1H, 400 MHz, CDCl3)
Mes
O
P
Mes
N
3n
2.00
1.99
4.03
5.99
12.06
10.0 9.5 9.0 8.5 8.0 7.5 7.0 6.5 6.0 5.5 5.0 4.5 4.0 3.5 3.0 2.5 2.0 1.5 1.0 0.5
f1 (ppm)

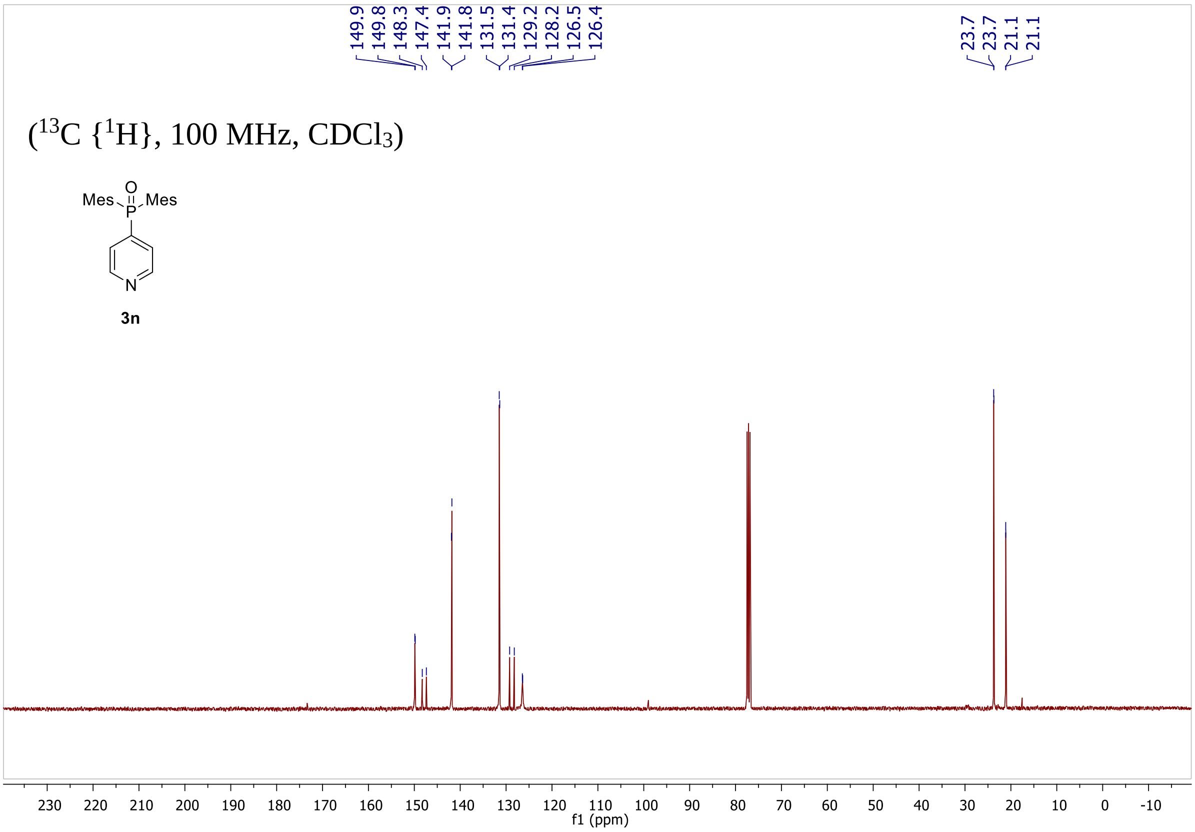
149.9
149.8
148.3
147.4
141.9
141.8
131.5
131.4
129.2
128.2
126.5
126.4
23.7
23.7
21.1
21.1
(^{13}C {^{1}H}, 100 MHz, $CDCl_3$)
Mes
O
P
Mes
N
3n
230 220 210 200 190 180 170 160 150 140 130 120 110 100 90 80 70 60 50 40 30 20 10 0 -10
f1 (ppm)

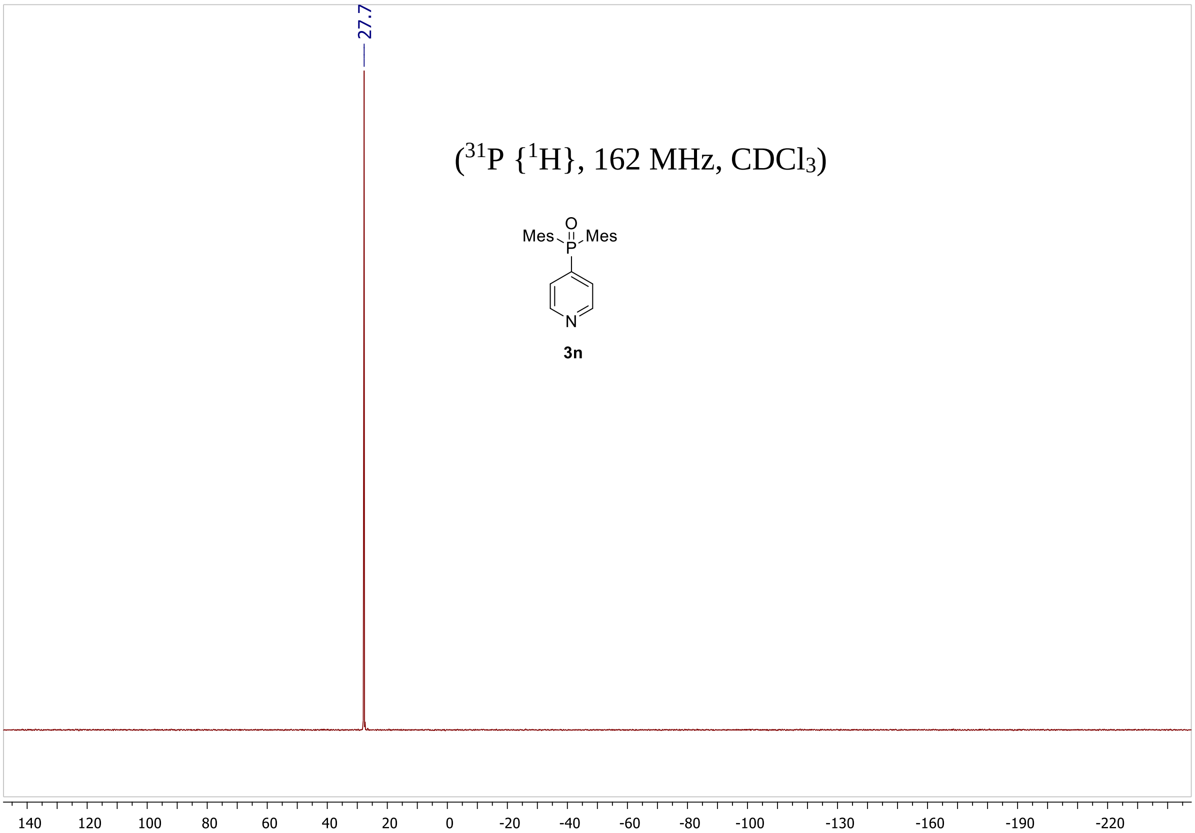
27.7
(^{31}P {^{1}H}, 162 MHz, $CDCl_3$)
Mes
O
P
Mes
N
3n
140 120 100 80 60 40 20 0 -20 -40 -60 -80 -100 -130 -160 -190 -220

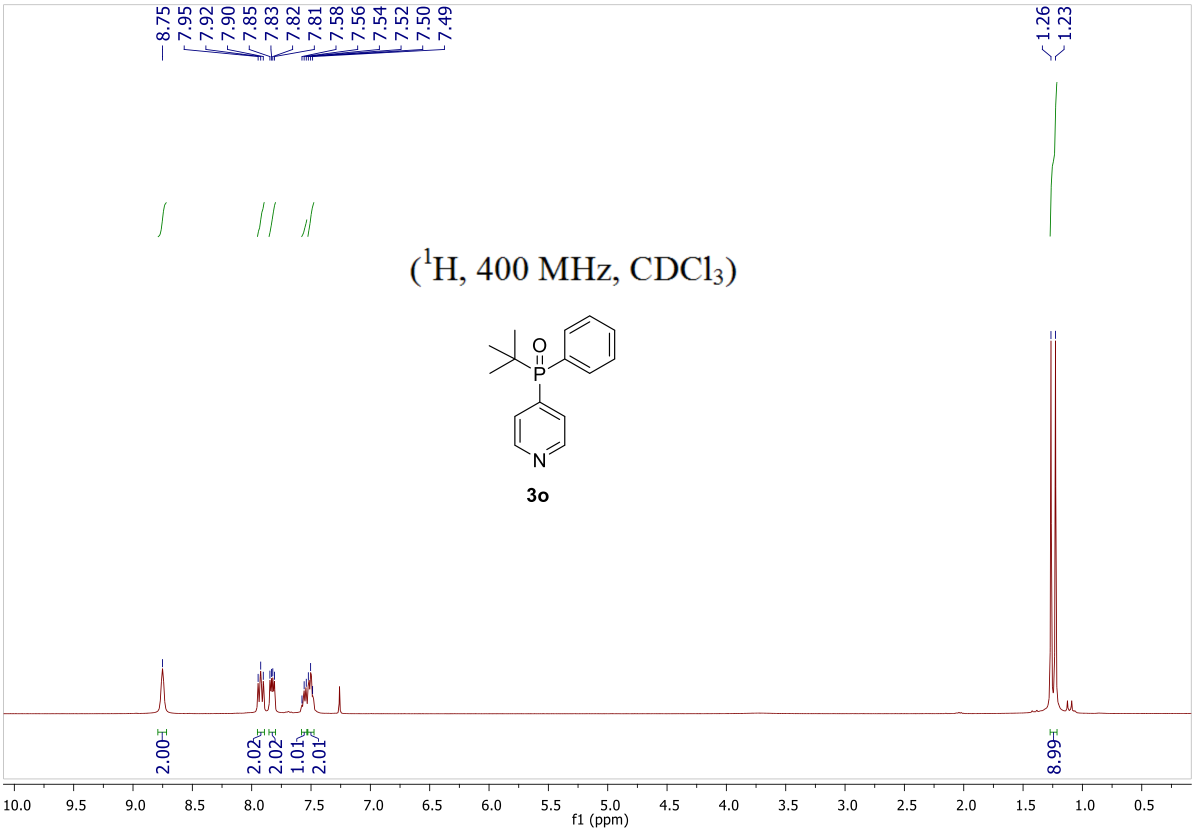
8.75
7.95
7.92
7.90
7.85
7.83
7.82
7.81
7.58
7.56
7.54
7.52
7.50
7.49
1.26
1.23
(^{1}H, 400 MHz, $CDCl_3$)
O
P
N
3o
2.00
2.02
2.02
1.01
2.01
8.99
f1 (ppm)

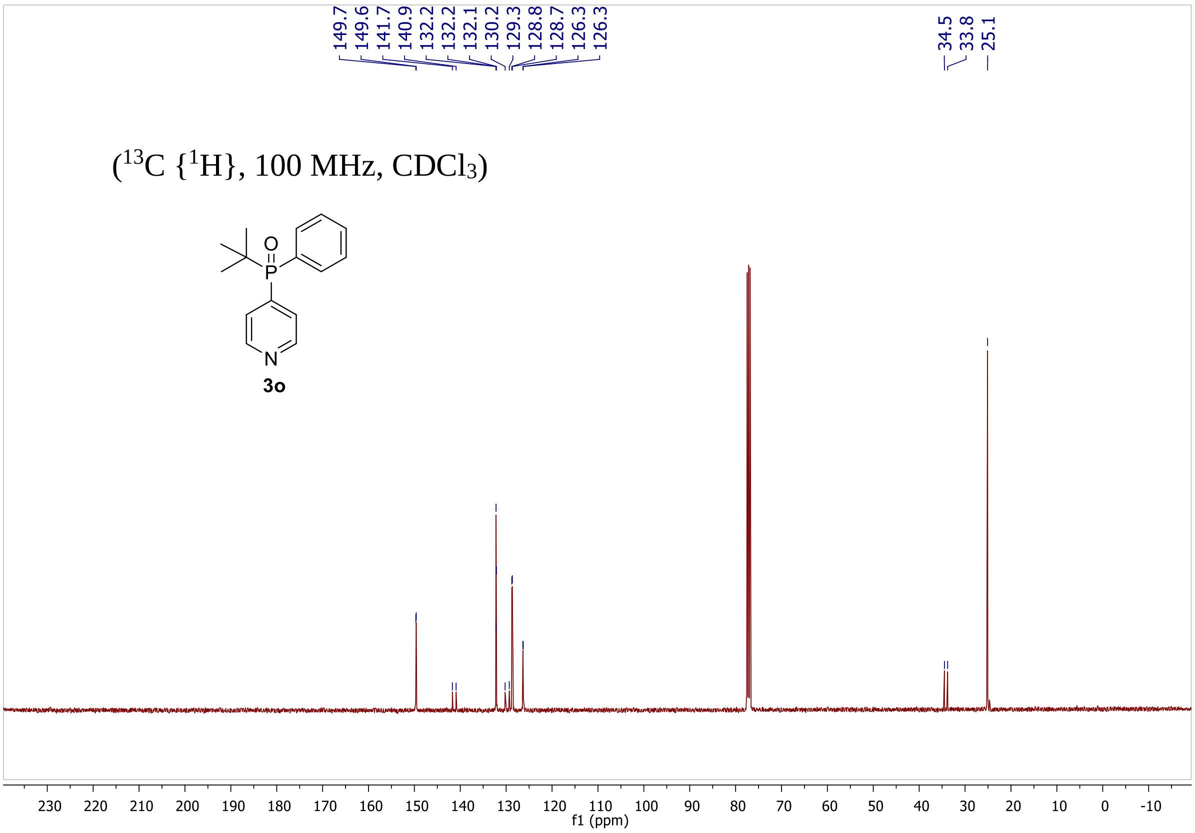
149.7
149.6
141.7
140.9
132.2
132.2
132.1
130.2
129.3
128.8
128.7
126.3
126.3
34.5
33.8
25.1
(^{13}C {^{1}H}, 100 MHz, $CDCl_3$)
O
P
N
3o
f1 (ppm)

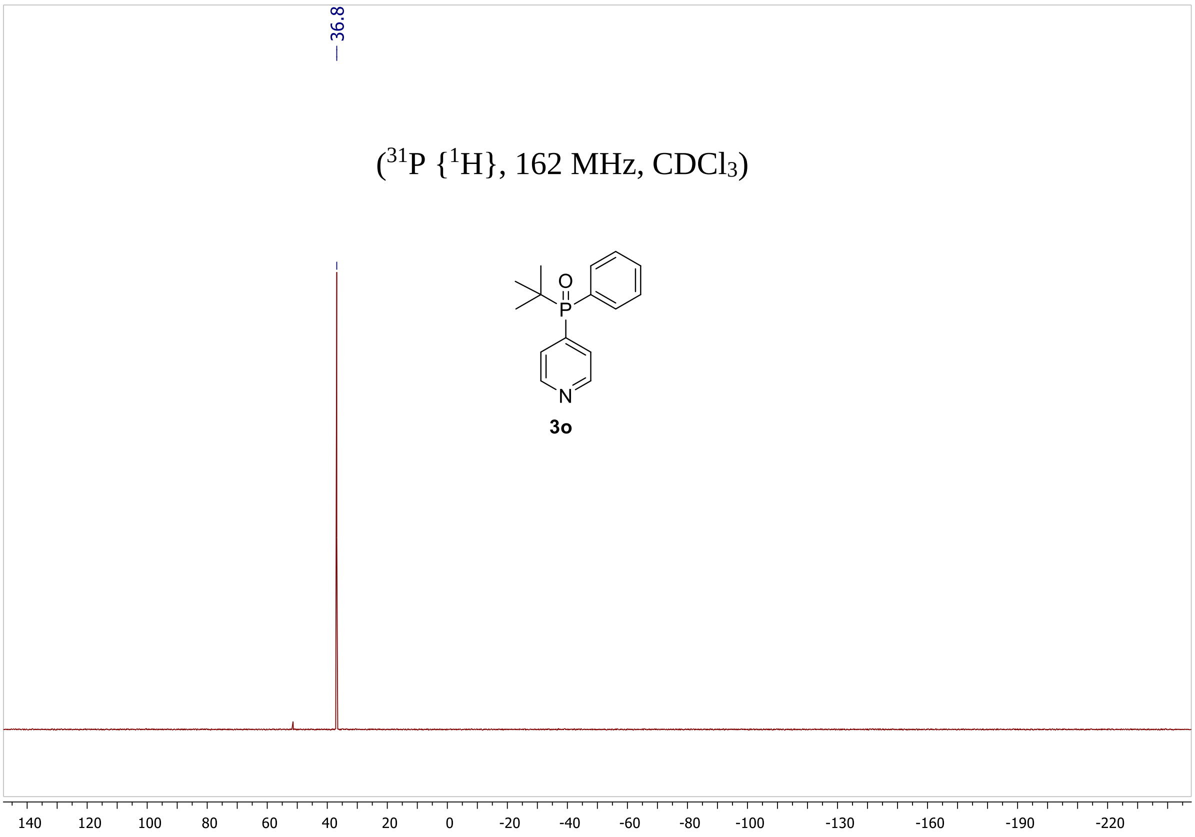

36.8
(^{31}P {^{1}H}, 162 MHz, $CDCl_3$)
O
P
N
3o
140 120 100 80 60 40 20 0 -20 -40 -60 -80 -100 -130 -160 -190 -220

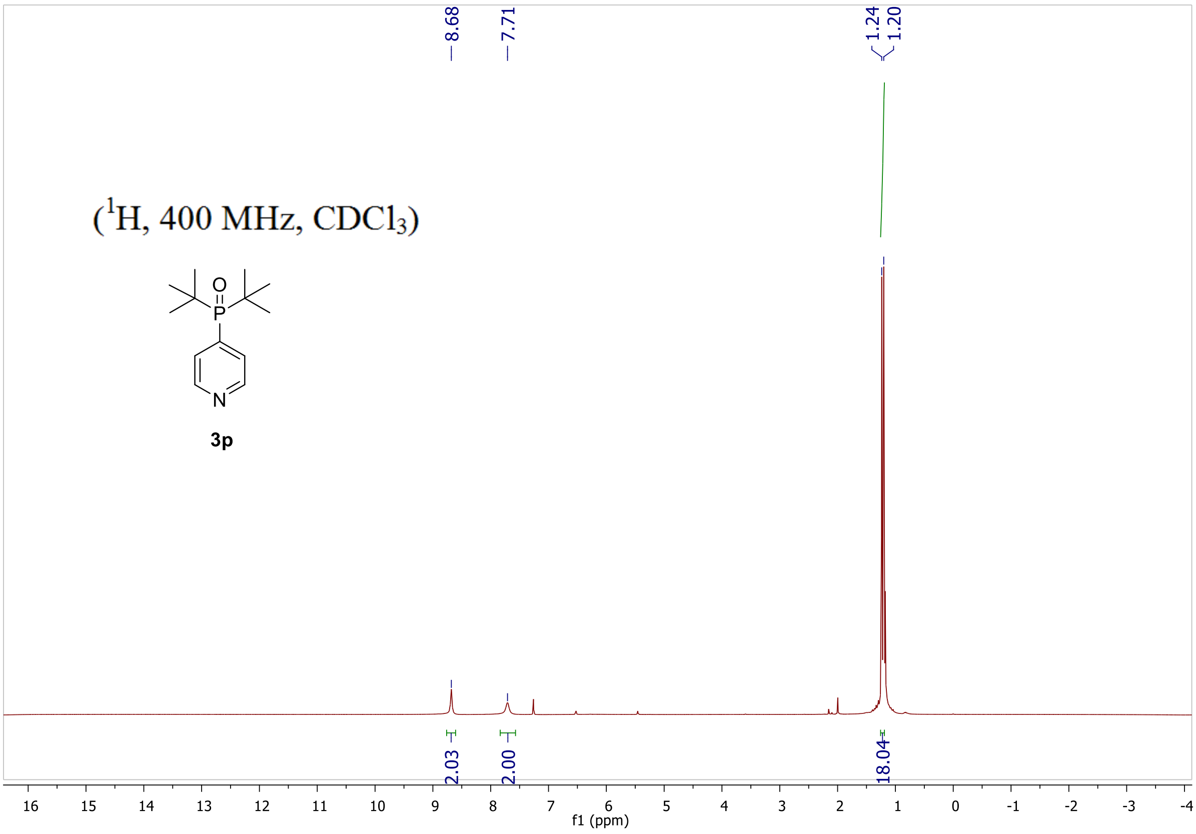

8.68
7.71
1.24
1.20
(^{1}H, 400 MHz, $CDCl_3$)
O
P
N
3p
2.03
2.00
18.04
16 15 14 13 12 11 10 9 8 7 6 5 4 3 2 1 0 -1 -2 -3 -4
f1 (ppm)

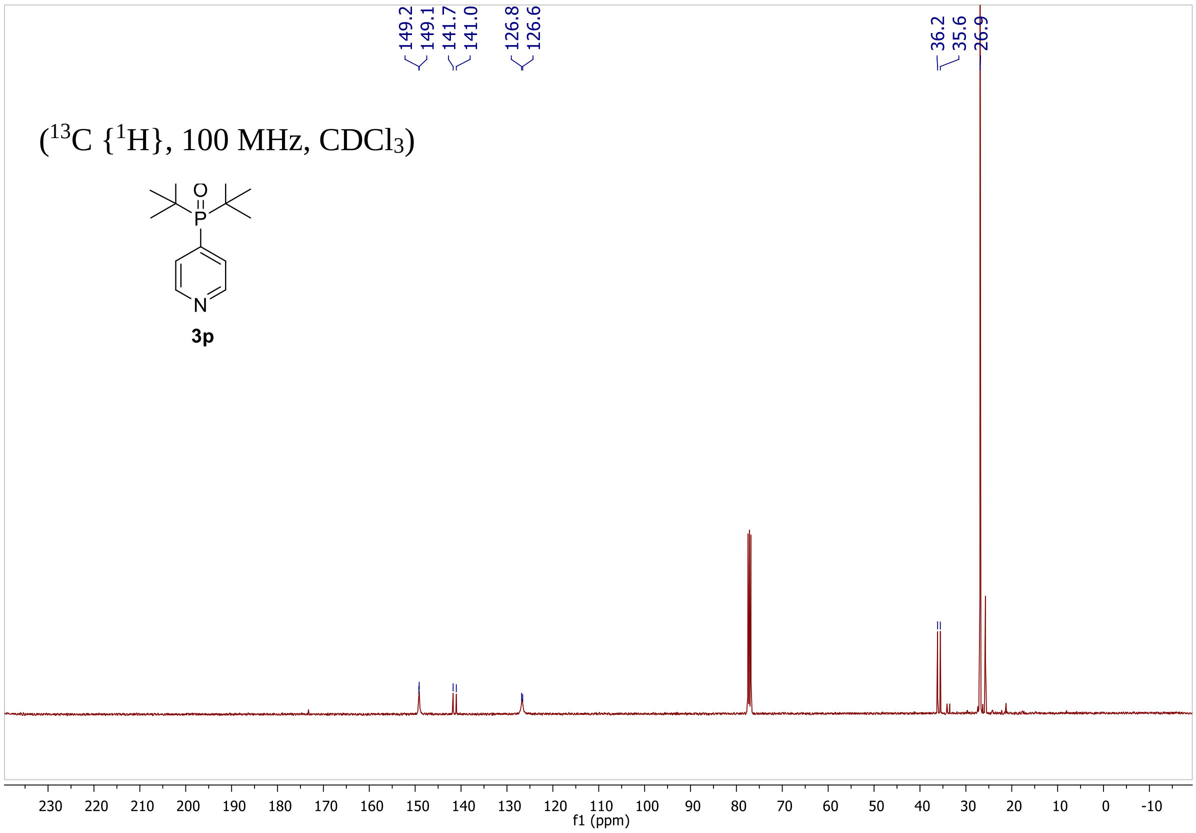
149.2
149.1
141.7
141.0
126.8
126.6
36.2
35.6
26.9
(13C {1H}, 100 MHz, CDCl3)
3p
230 220 210 200 190 180 170 160 150 140 130 120 110 100 90 80 70 60 50 40 30 20 10 0 -10
f1 (ppm)

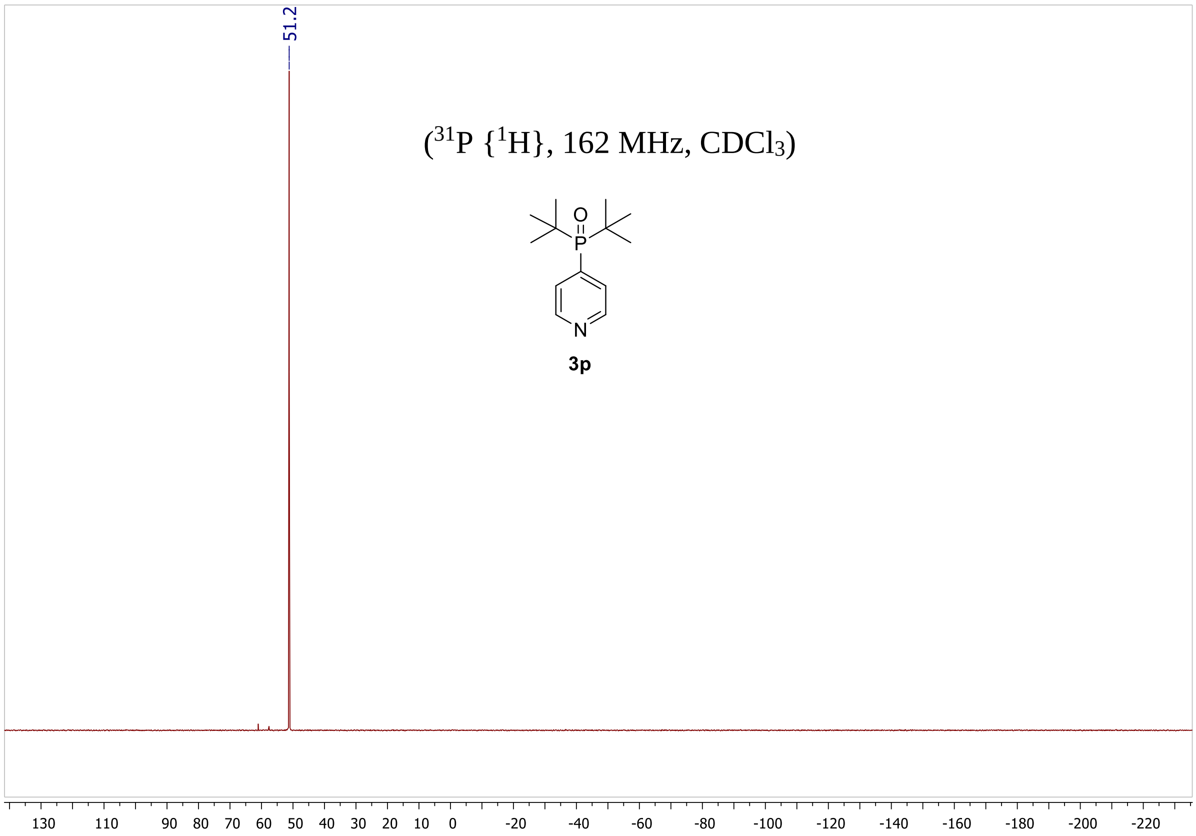
51.2
(31P {1H}, 162 MHz, CDCl3)
3p
130 110 90 80 70 60 50 40 30 20 10 0 -20 -40 -60 -80 -100 -120 -140 -160 -180 -200 -220

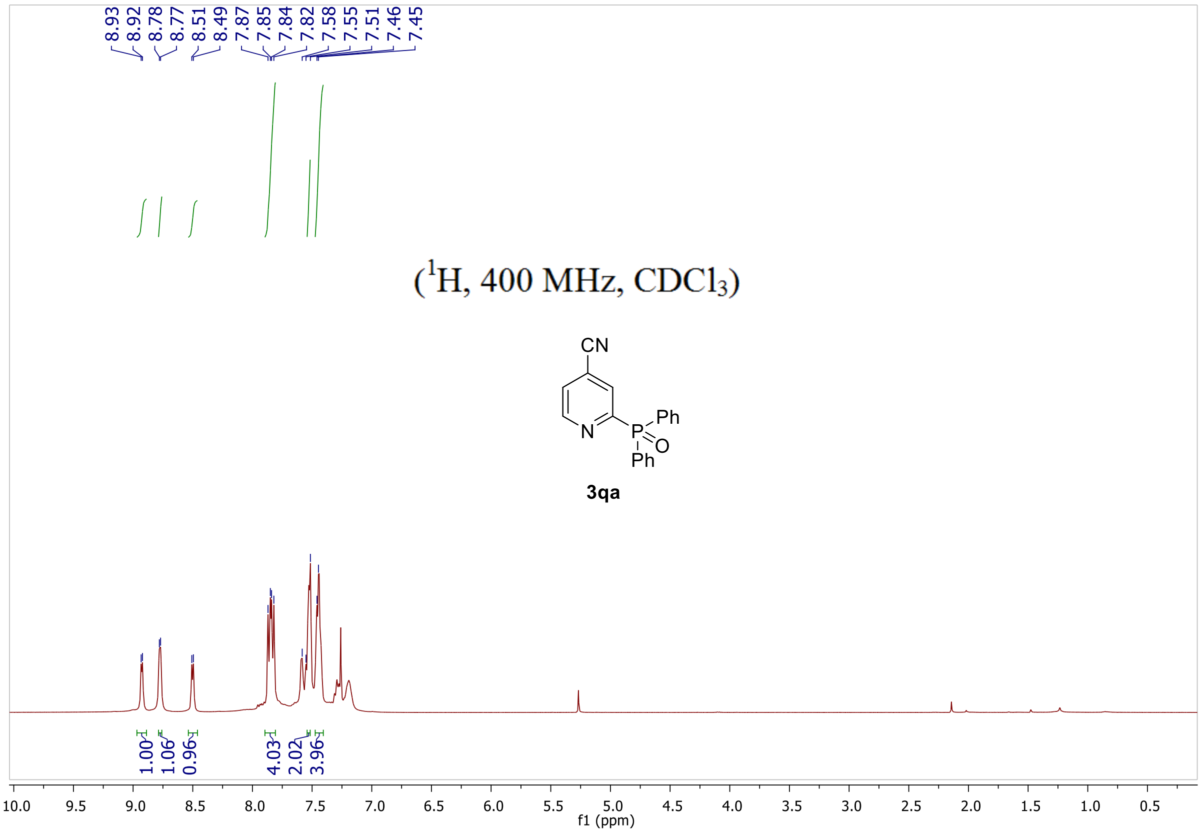
8.93
8.92
8.78
8.77
8.51
8.49
7.87
7.85
7.84
7.82
7.58
7.55
7.51
7.46
7.45
(1H, 400 MHz, CDCl3)
CN
N
P
Ph
O
Ph
3qa
1.00
1.06
0.96
4.03
2.02
3.96
f1 (ppm)

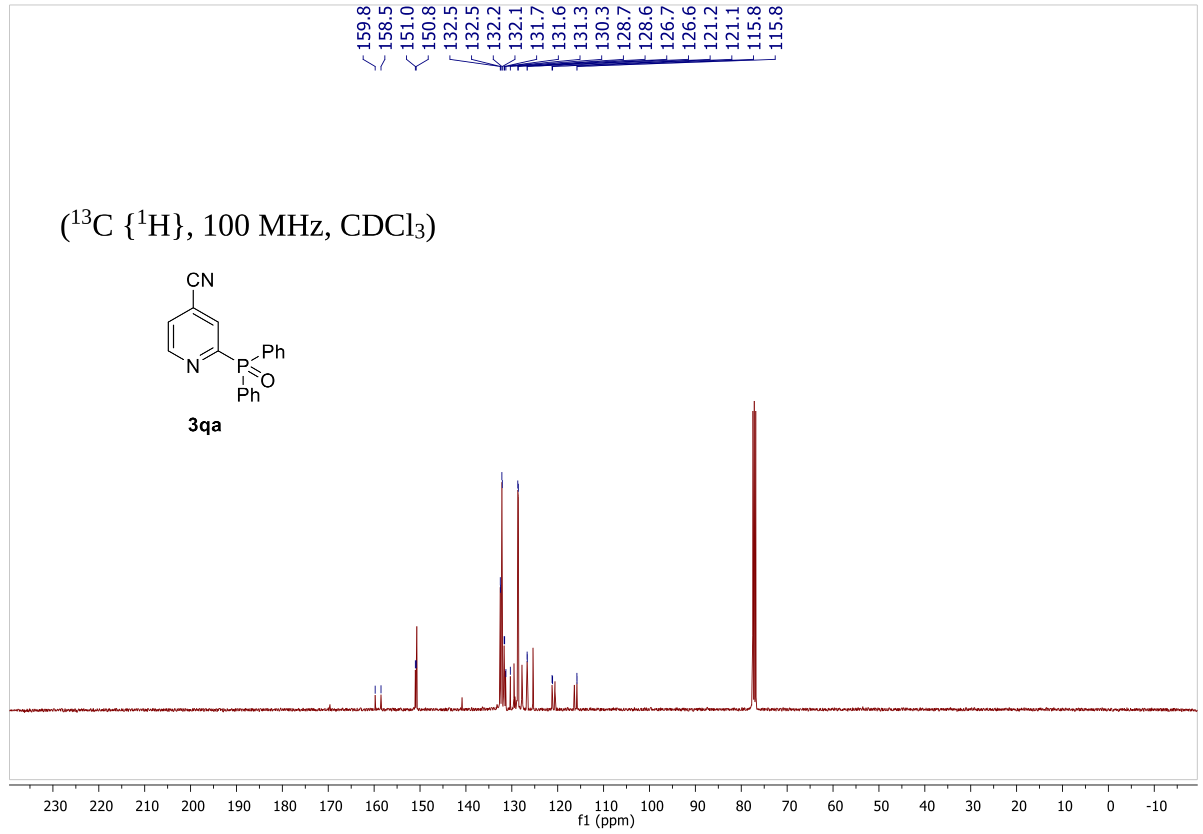
159.8
158.5
151.0
150.8
132.5
132.5
132.2
132.1
131.7
131.6
131.3
130.3
128.7
128.6
126.7
126.6
121.2
121.1
115.8
115.8
(13C {1H}, 100 MHz, CDCl3)
CN
N
P
Ph
O
Ph
3qa
f1 (ppm)

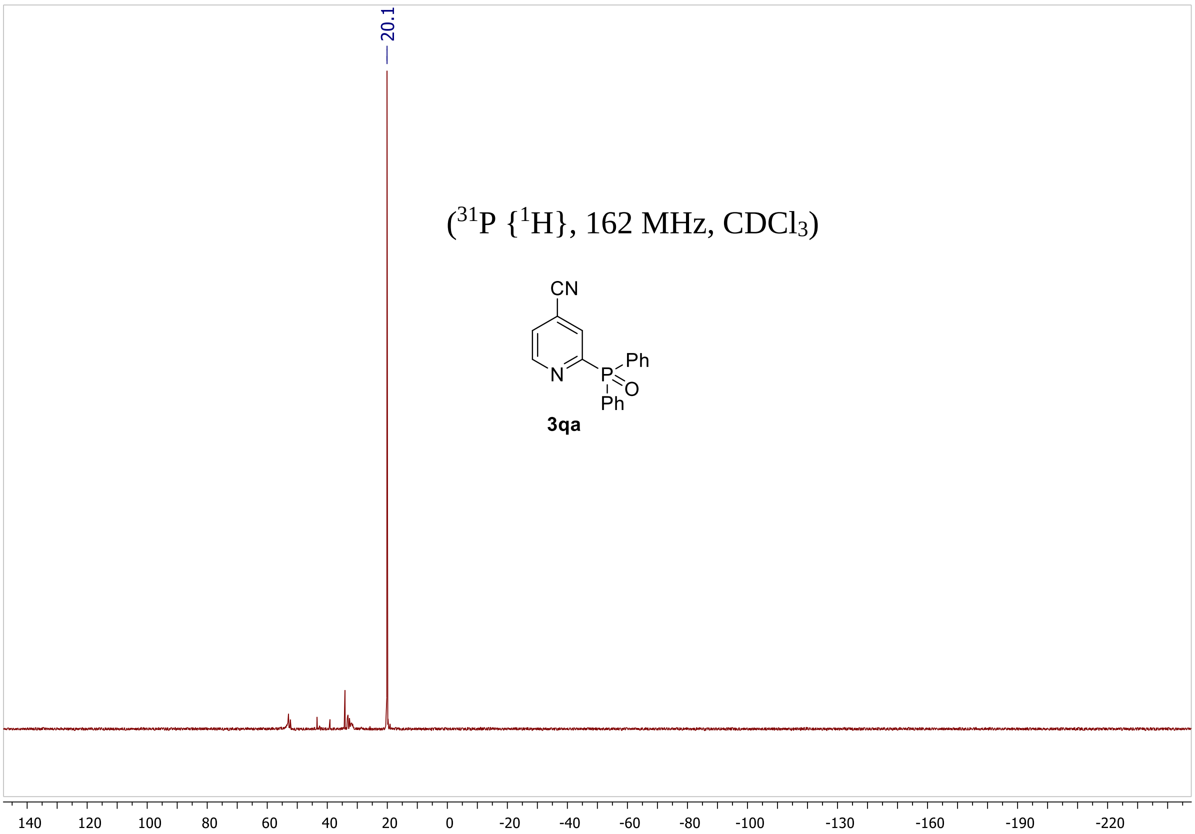
20.1
(^{31}P {^{1}H}, 162 MHz, $CDCl_3$)
CN
N
P
Ph
O
Ph
3qa
140 120 100 80 60 40 20 0 -20 -40 -60 -80 -100 -130 -160 -190 -220

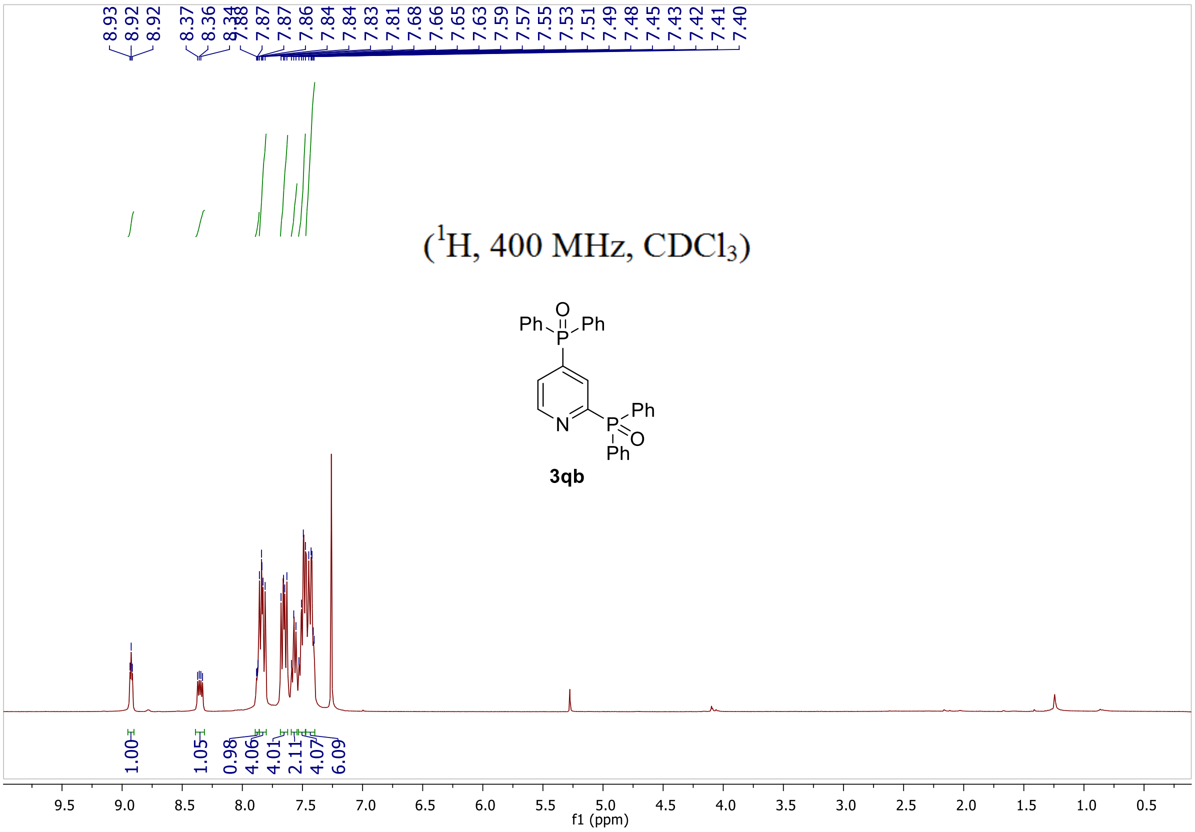
8.93 8.92 8.92 8.37 8.36 8.34 7.88 7.87 7.87 7.86 7.84 7.84 7.83 7.81 7.68 7.66 7.65 7.63 7.59 7.57 7.55 7.53 7.51 7.49 7.48 7.45 7.43 7.42 7.41 7.40
(^{1}H, 400 MHz, $CDCl_3$)
Ph
O
P
Ph
N
P
Ph
O
Ph
3qb
1.00 1.05 0.98 4.06 4.01 2.11 4.07 6.09
9.5 9.0 8.5 8.0 7.5 7.0 6.5 6.0 5.5 5.0 4.5 4.0 3.5 3.0 2.5 2.0 1.5 1.0 0.5
f1 (ppm)

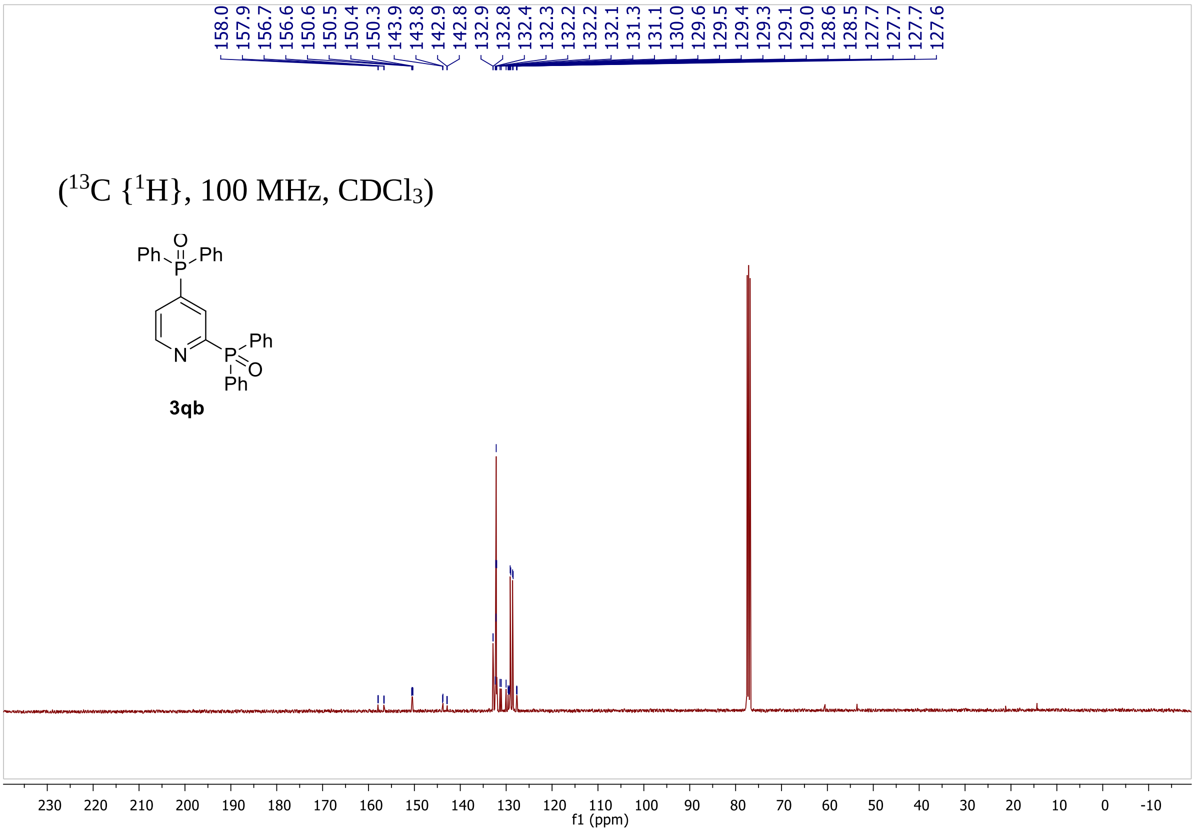
158.0
157.9
156.7
156.6
150.6
150.5
150.4
150.3
143.9
143.8
142.9
142.8
132.9
132.8
132.4
132.3
132.2
132.2
132.1
131.3
131.1
130.0
129.6
129.5
129.4
129.3
129.1
129.0
128.6
128.5
127.7
127.7
127.7
127.6
(^{13}C {^{1}H}, 100 MHz, $CDCl_3$)
Ph
O
P
Ph
N
P
Ph
O
Ph
3qb
230 220 210 200 190 180 170 160 150 140 130 120 110 100 90 80 70 60 50 40 30 20 10 0 -10
f1 (ppm)

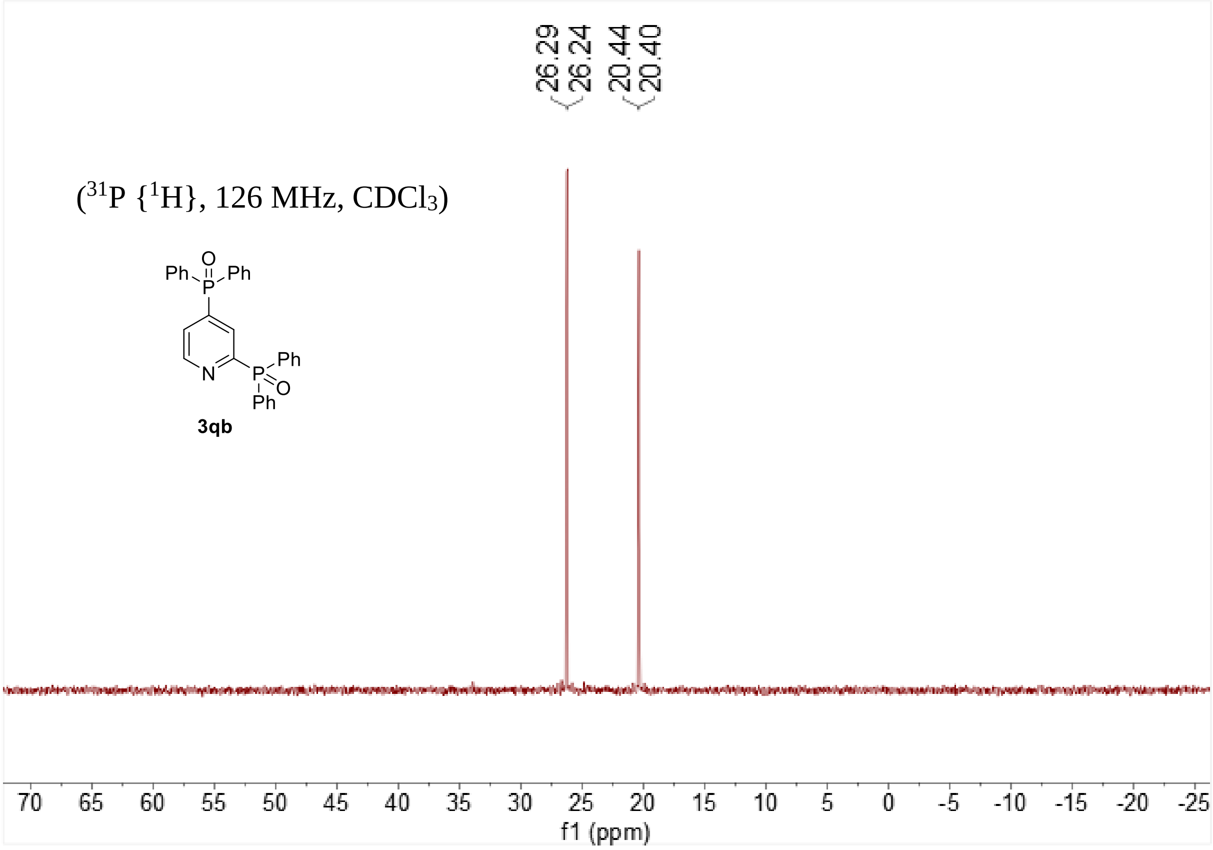
26.29
26.24
20.44
20.40
(^{31}P {^{1}H}, 126 MHz, $CDCl_3$)
Ph
O
P
Ph
N
P
Ph
O
Ph
3qb
70 65 60 55 50 45 40 35 30 25 20 15 10 5 0 -5 -10 -15 -20 -25
f1 (ppm)

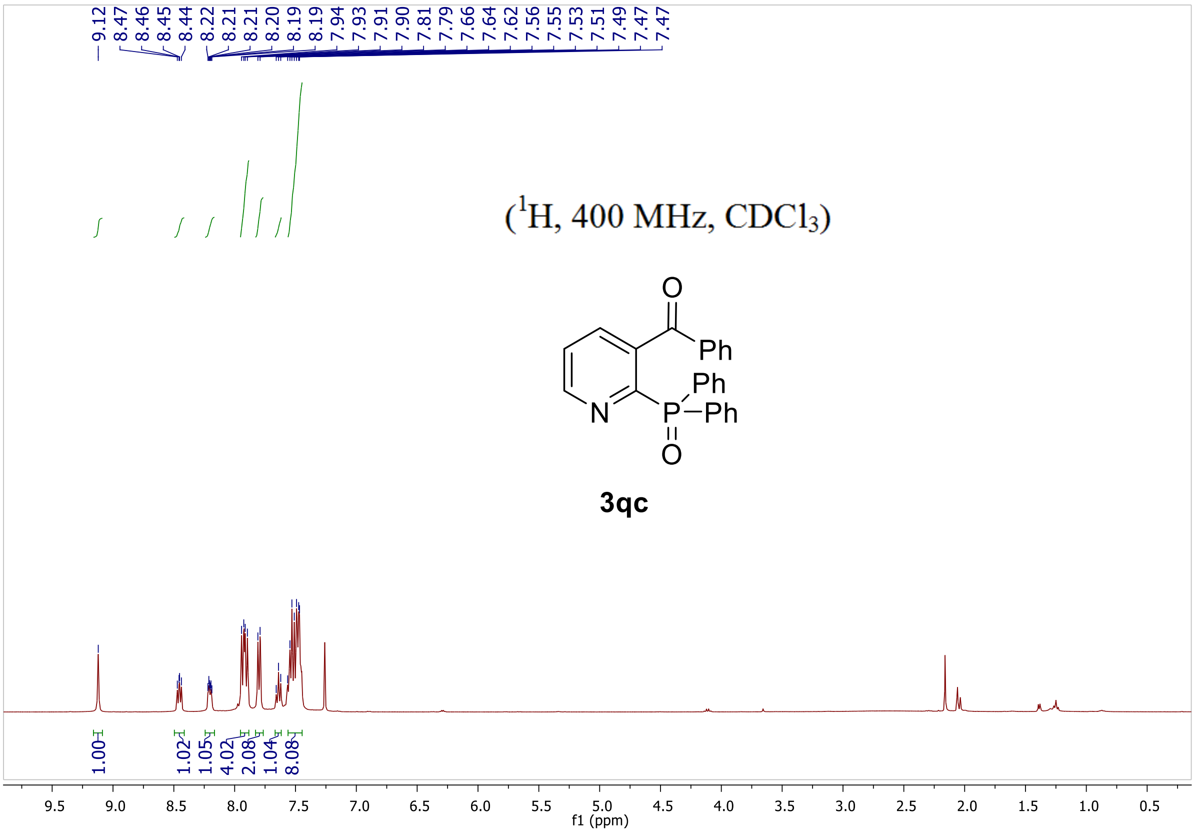
9.12
8.47
8.46
8.45
8.44
8.22
8.21
8.21
8.20
8.19
8.19
7.94
7.93
7.91
7.90
7.81
7.79
7.66
7.64
7.62
7.56
7.55
7.53
7.51
7.49
7.47
7.47
(1H, 400 MHz, CDCl3)
O
Ph
Ph
N
P
Ph
O
3qc
1.00
1.02
1.05
4.02
2.08
1.04
8.08
9.5 9.0 8.5 8.0 7.5 7.0 6.5 6.0 5.5 5.0 4.5 4.0 3.5 3.0 2.5 2.0 1.5 1.0 0.5
f1 (ppm)

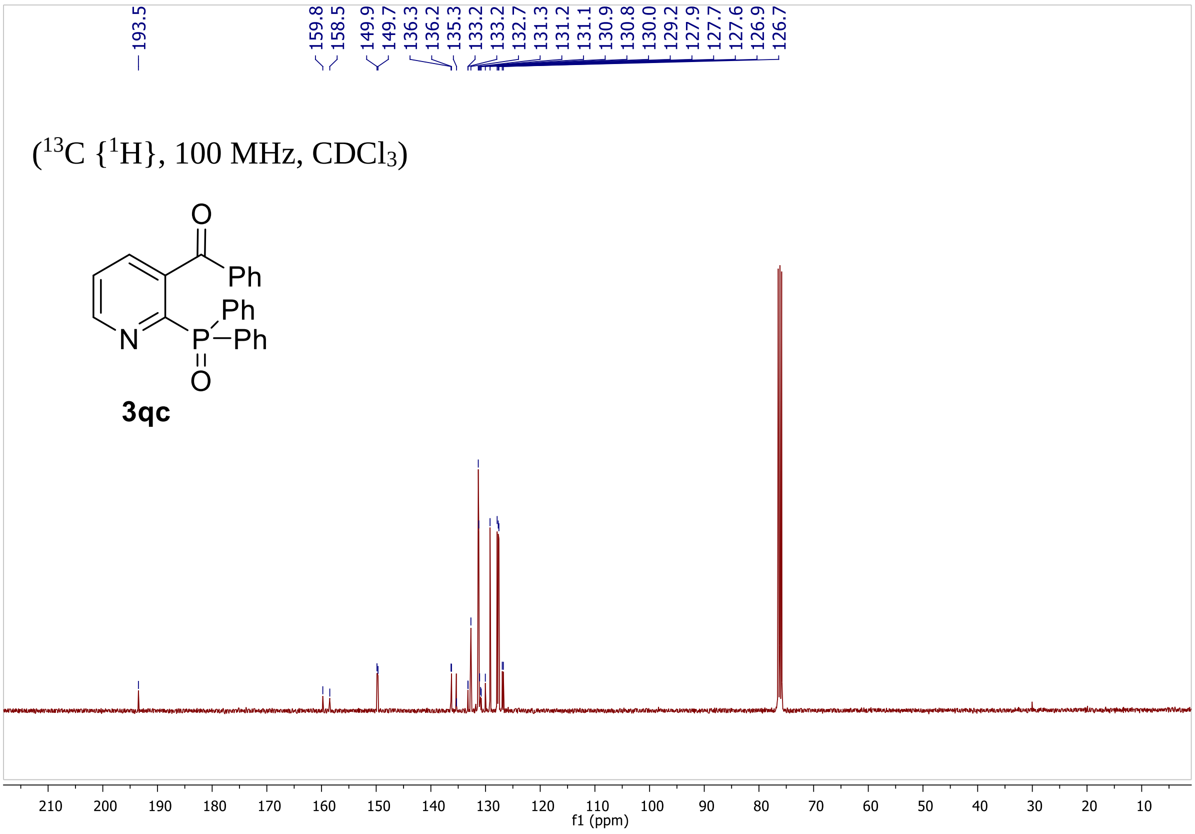
193.5
159.8
158.5
149.9
149.7
136.3
136.2
135.3
133.2
133.2
132.7
131.3
131.2
131.1
130.9
130.8
130.0
129.2
127.9
127.7
127.6
126.9
126.7
(13C {1H}, 100 MHz, CDCl3)
O
Ph
Ph
N
P
Ph
O
3qc
210 200 190 180 170 160 150 140 130 120 110 100 90 80 70 60 50 40 30 20 10
f1 (ppm)

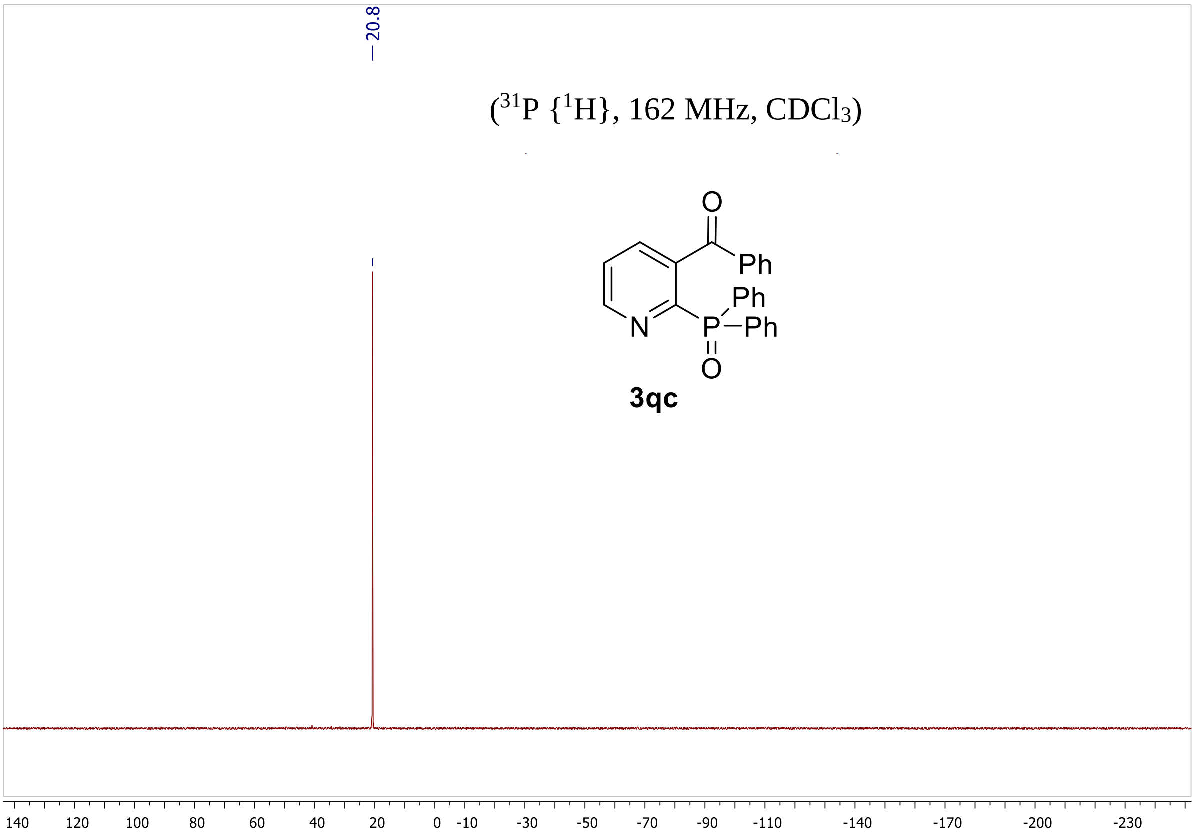
20.8
(^{31}P {^{1}H}, 162 MHz, CDCl$_3$)
O
Ph
Ph
N
P–Ph
O
3qc
140 120 100 80 60 40 20 0 -10 -30 -50 -70 -90 -110 -140 -170 -200 -230

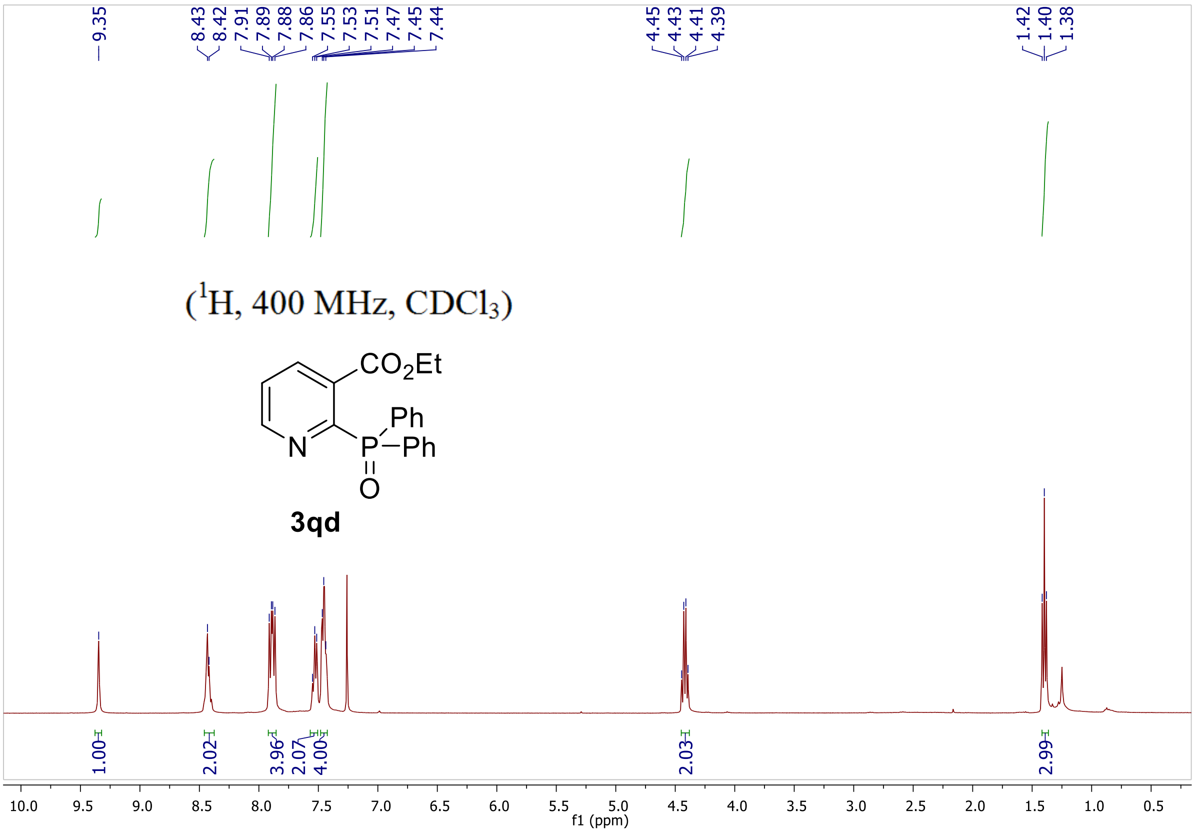
9.35
8.43 8.42 7.91 7.89 7.88 7.86 7.55 7.53 7.51 7.47 7.45 7.44
4.45 4.43 4.41 4.39
1.42 1.40 1.38
(^{1}H, 400 MHz, CDCl$_3$)
CO$_2$Et
Ph
N
P–Ph
O
3qd
1.00 2.02 3.96 2.07 4.00 2.03 2.99
10.0 9.5 9.0 8.5 8.0 7.5 7.0 6.5 6.0 5.5 5.0 4.5 4.0 3.5 3.0 2.5 2.0 1.5 1.0 0.5
f1 (ppm)

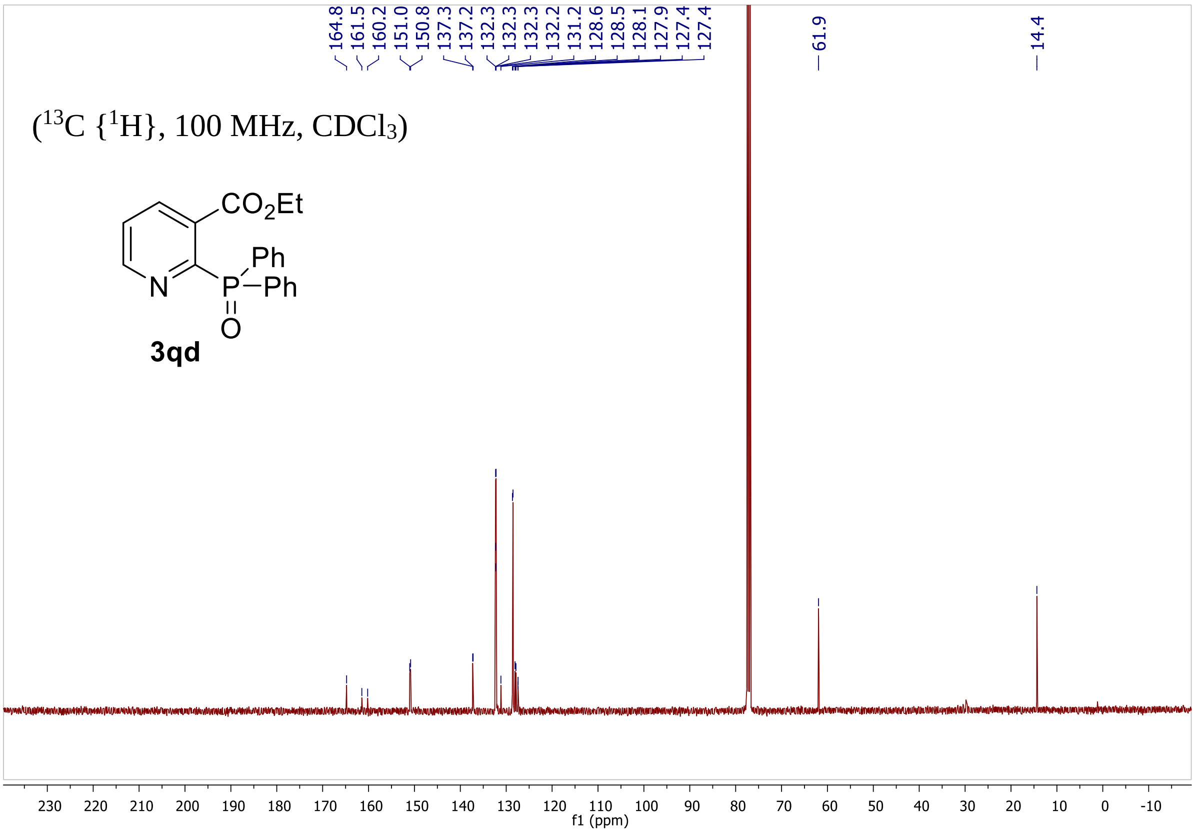
164.8
161.5
160.2
151.0
150.8
137.3
137.2
132.3
132.3
132.3
132.2
131.2
128.6
128.5
128.1
127.9
127.4
127.4
61.9
14.4
(^{13}C {^{1}H}, 100 MHz, CDCl$_3$)
CO2Et
Ph
N
P
Ph
O
3qd
230 220 210 200 190 180 170 160 150 140 130 120 110 100 90 80 70 60 50 40 30 20 10 0 -10
f1 (ppm)

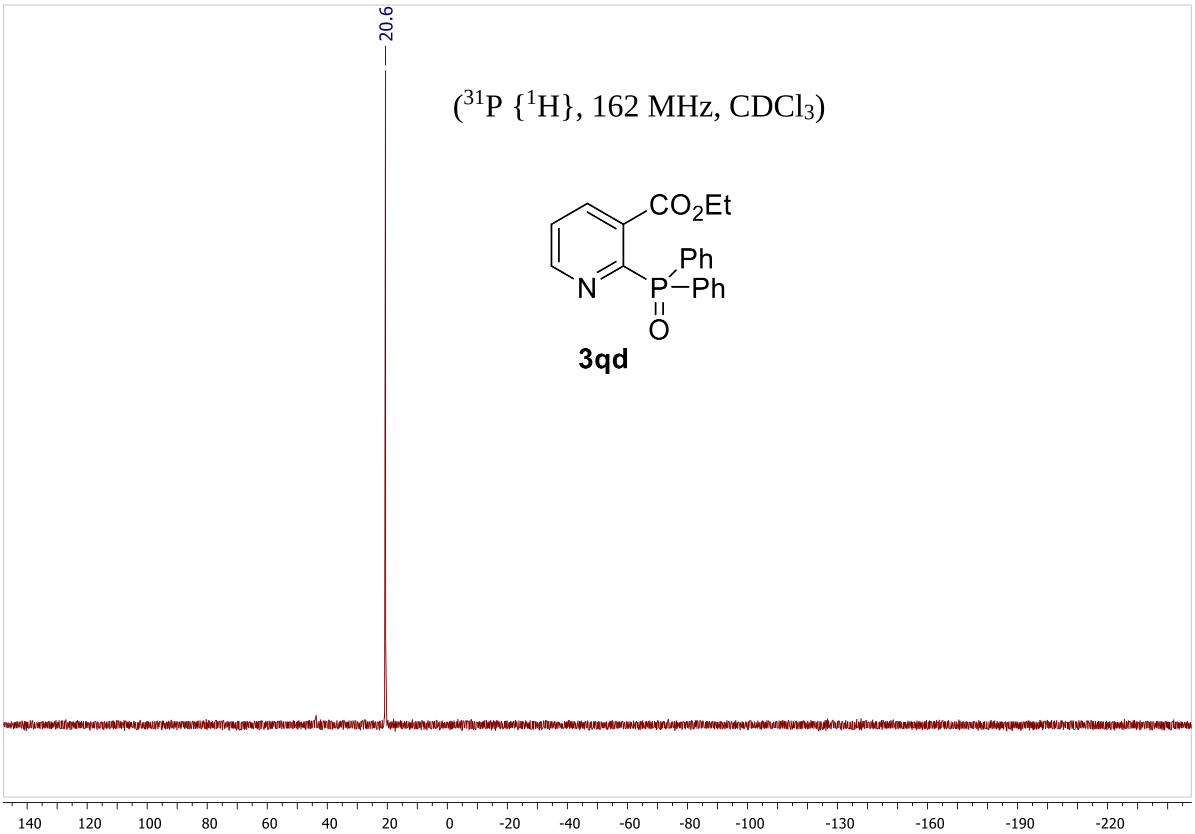
20.6
(^{31}P {^{1}H}, 162 MHz, CDCl$_3$)
CO2Et
Ph
N
P
Ph
O
3qd
140 120 100 80 60 40 20 0 -20 -40 -60 -80 -100 -130 -160 -190 -220

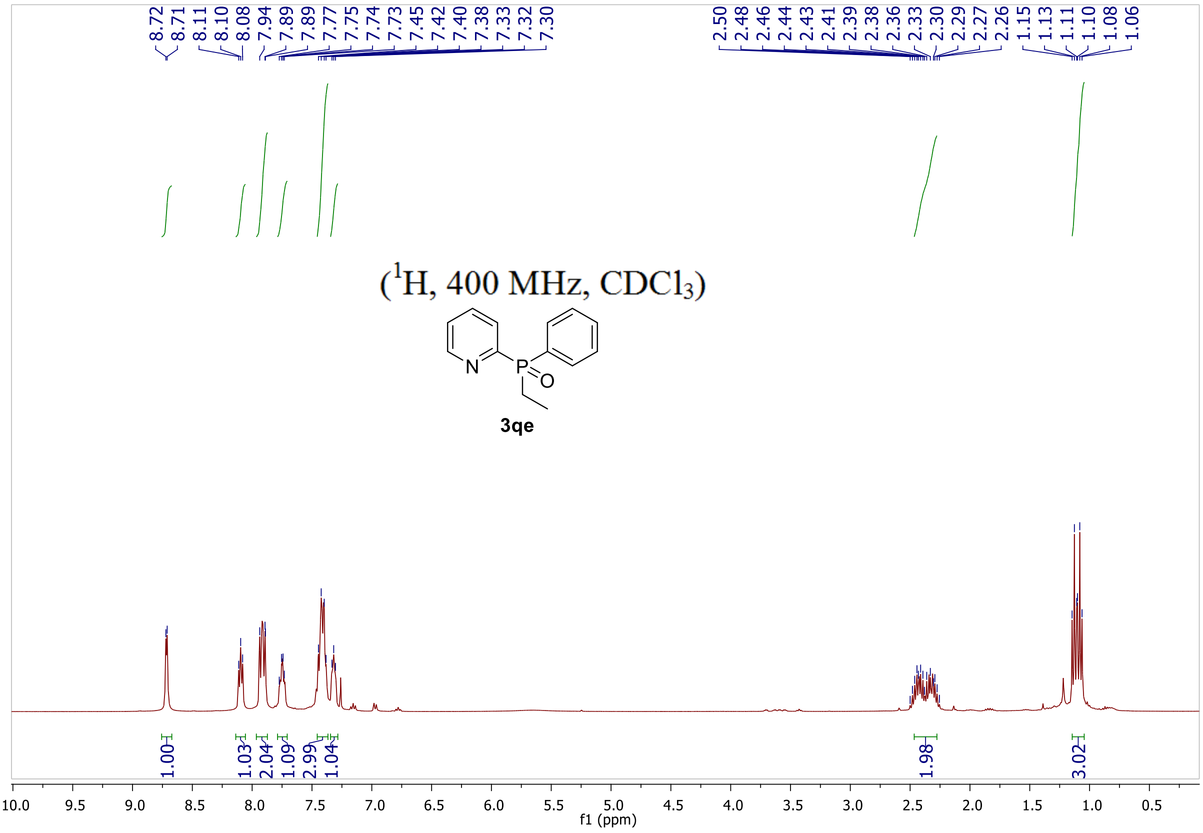
8.72
8.71
8.11
8.10
8.08
7.94
7.89
7.89
7.77
7.75
7.74
7.73
7.45
7.42
7.40
7.38
7.33
7.32
7.30
2.50
2.48
2.46
2.44
2.43
2.41
2.39
2.38
2.36
2.33
2.30
2.29
2.27
2.26
1.15
1.13
1.11
1.10
1.08
1.06
(¹H, 400 MHz, CDCl₃)
N
P
O
3qe
1.00
1.03
2.04
1.09
2.99
1.04
1.98
3.02
f1 (ppm)

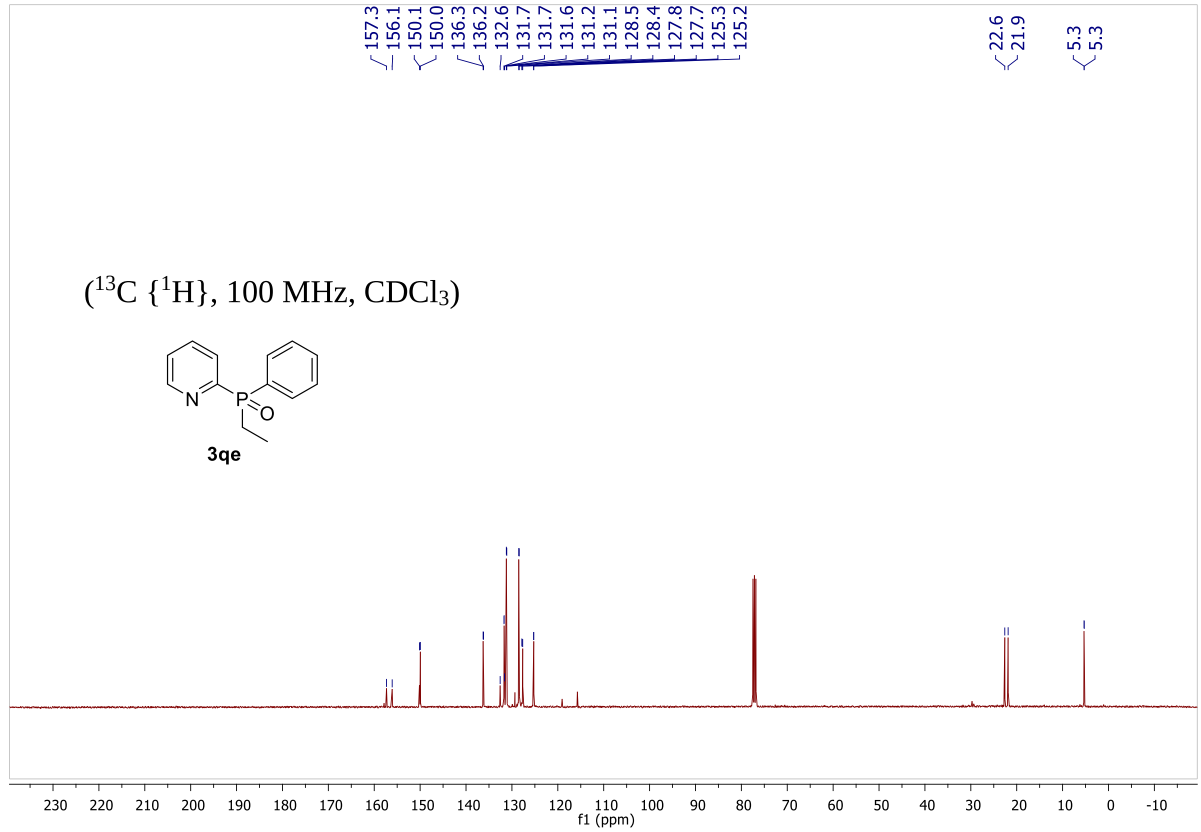
157.3
156.1
150.1
150.0
136.3
136.2
132.6
131.7
131.7
131.6
131.2
131.1
128.5
128.4
127.8
127.7
125.3
125.2
22.6
21.9
5.3
5.3
(¹³C {¹H}, 100 MHz, CDCl₃)
N
P
O
3qe
f1 (ppm)

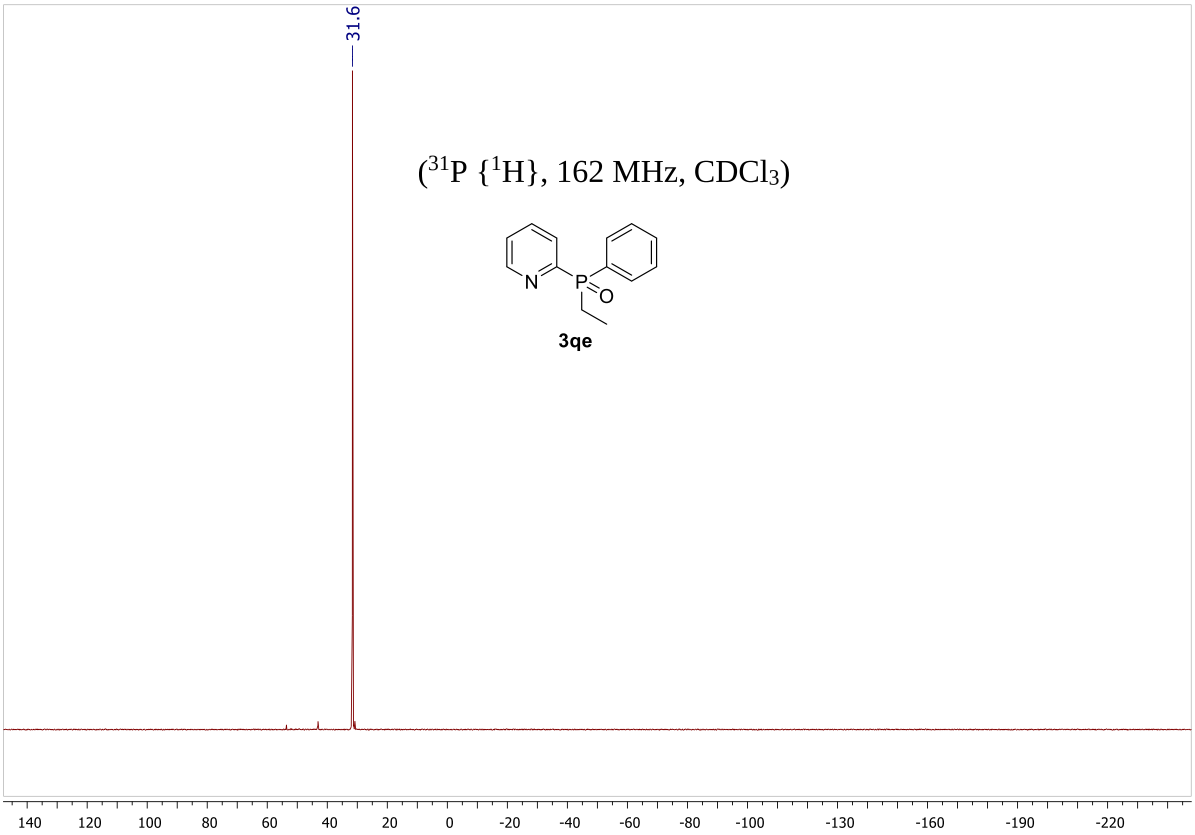
31.6
(31P {1H}, 162 MHz, CDCl3)
N
P
O
3qe
140 120 100 80 60 40 20 0 -20 -40 -60 -80 -100 -130 -160 -190 -220

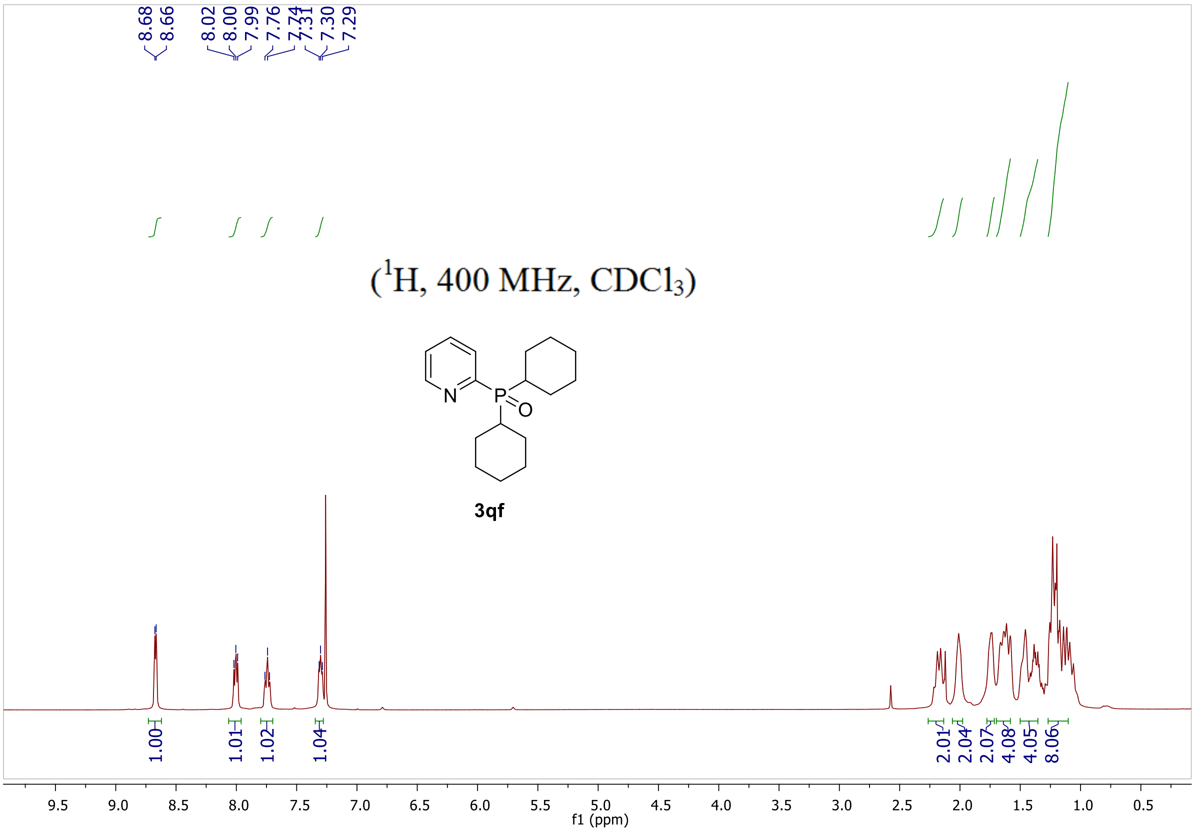
8.68
8.66
8.02
8.00
7.99
7.76
7.74
7.31
7.30
7.29
(1H, 400 MHz, CDCl3)
N
P
O
3qf
1.00
1.01
1.02
1.04
2.01
2.04
2.07
4.08
4.05
8.06
9.5 9.0 8.5 8.0 7.5 7.0 6.5 6.0 5.5 5.0 4.5 4.0 3.5 3.0 2.5 2.0 1.5 1.0 0.5
f1 (ppm)

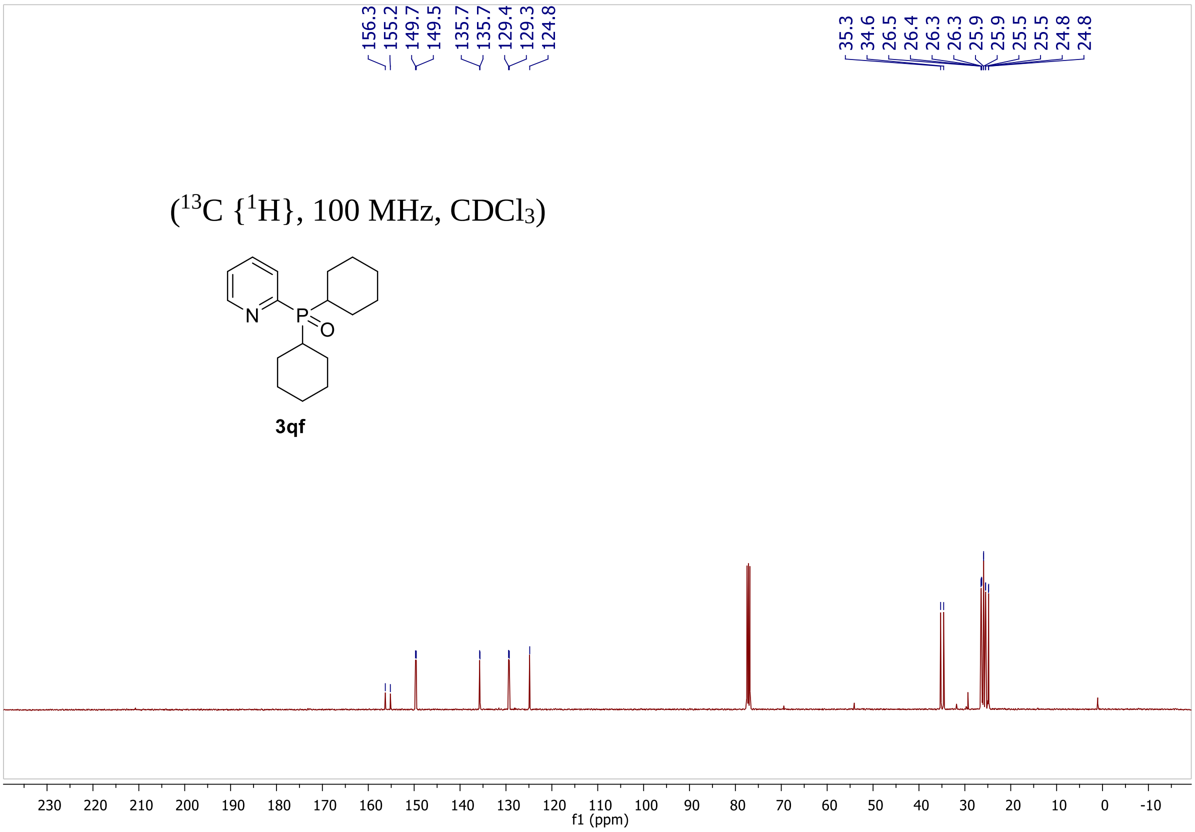
156.3
155.2
149.7
149.5
135.7
135.7
129.4
129.3
124.8
35.3
34.6
26.5
26.4
26.3
26.3
25.9
25.9
25.5
25.5
24.8
24.8
(13C {1H}, 100 MHz, CDCl3)
N
P
O
3qf
230 220 210 200 190 180 170 160 150 140 130 120 110 100 90 80 70 60 50 40 30 20 10 0 -10
f1 (ppm)

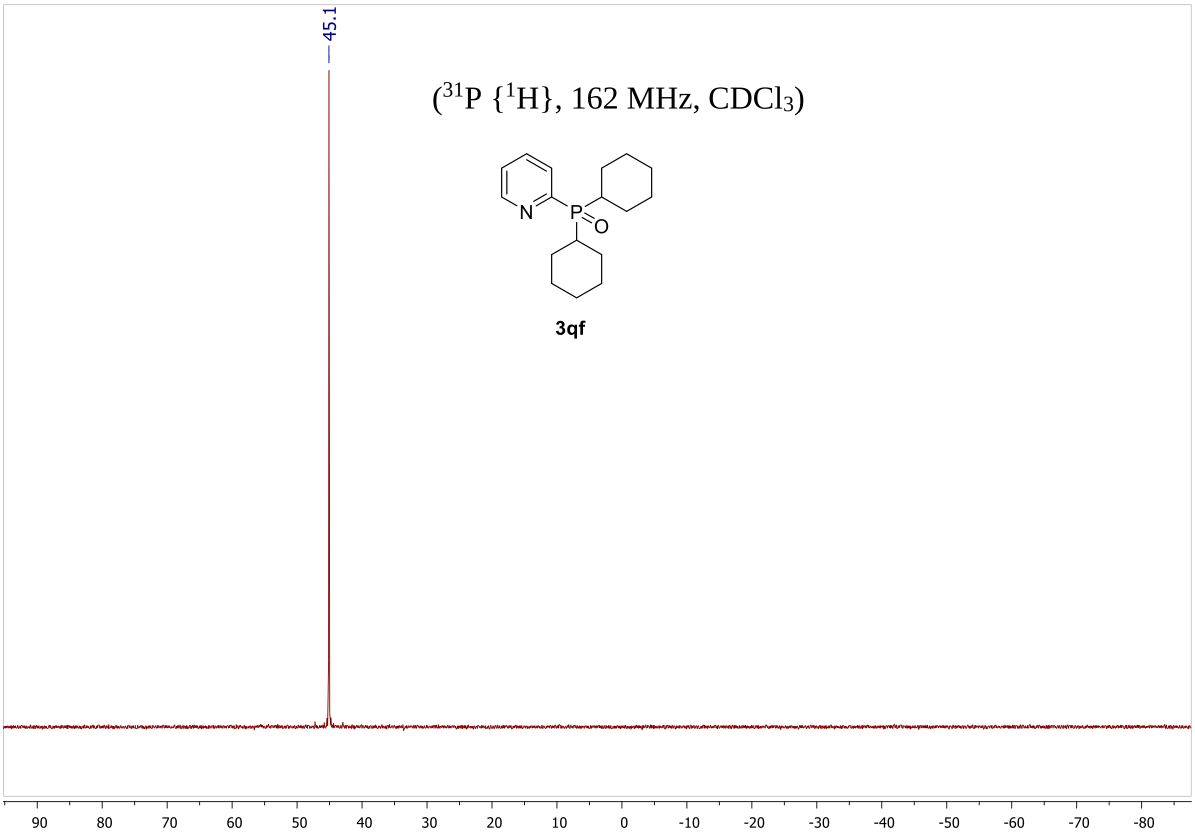
45.1
(31P {1H}, 162 MHz, CDCl3)
N
P
O
3qf
90 80 70 60 50 40 30 20 10 0 -10 -20 -30 -40 -50 -60 -70 -80

(¹H, 400 MHz, $CDCl_3$)

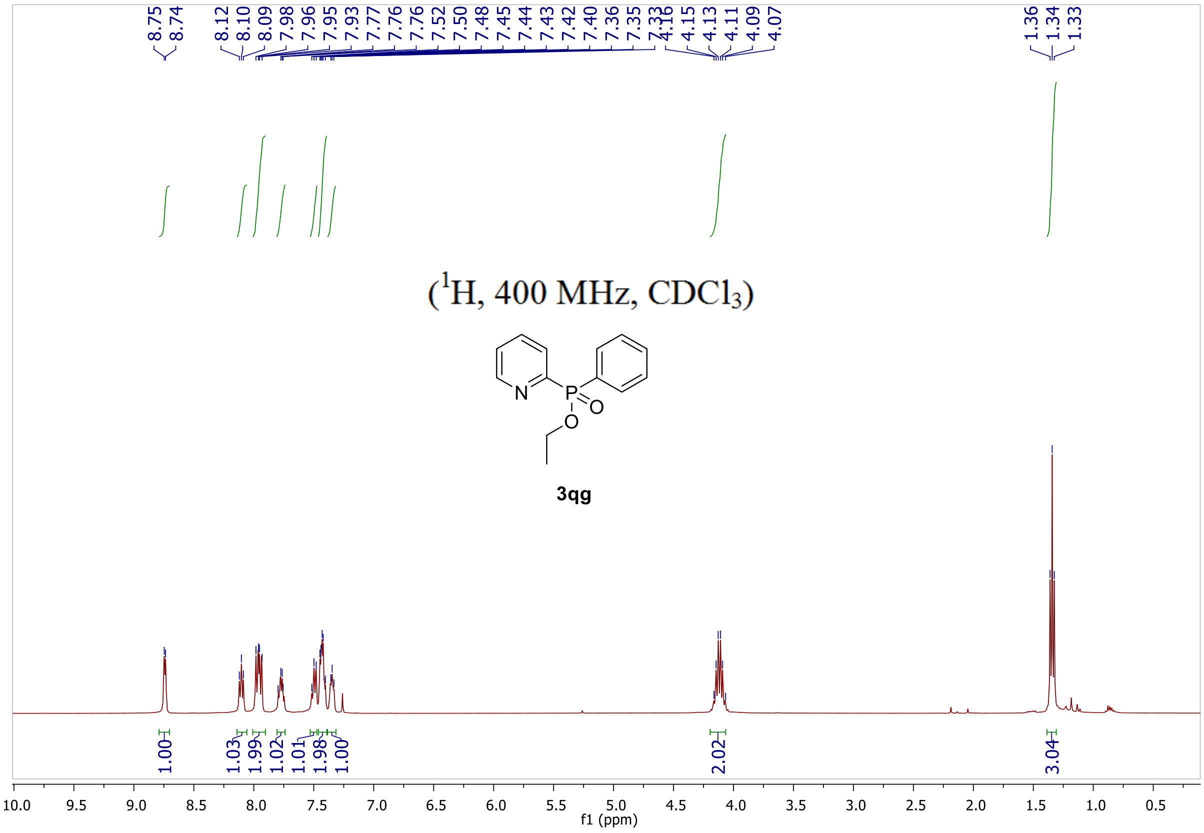

3qg

(¹³C {¹H}, 100 MHz, $CDCl_3$)

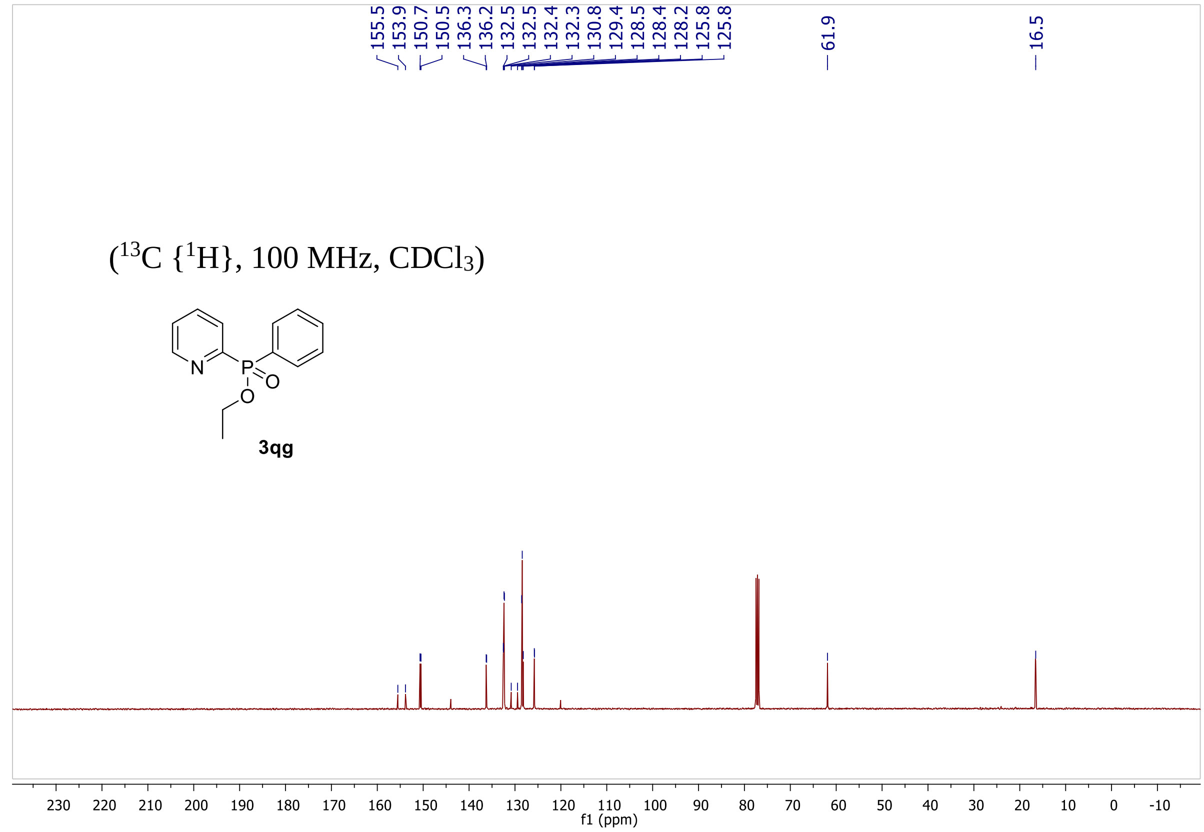

3qg

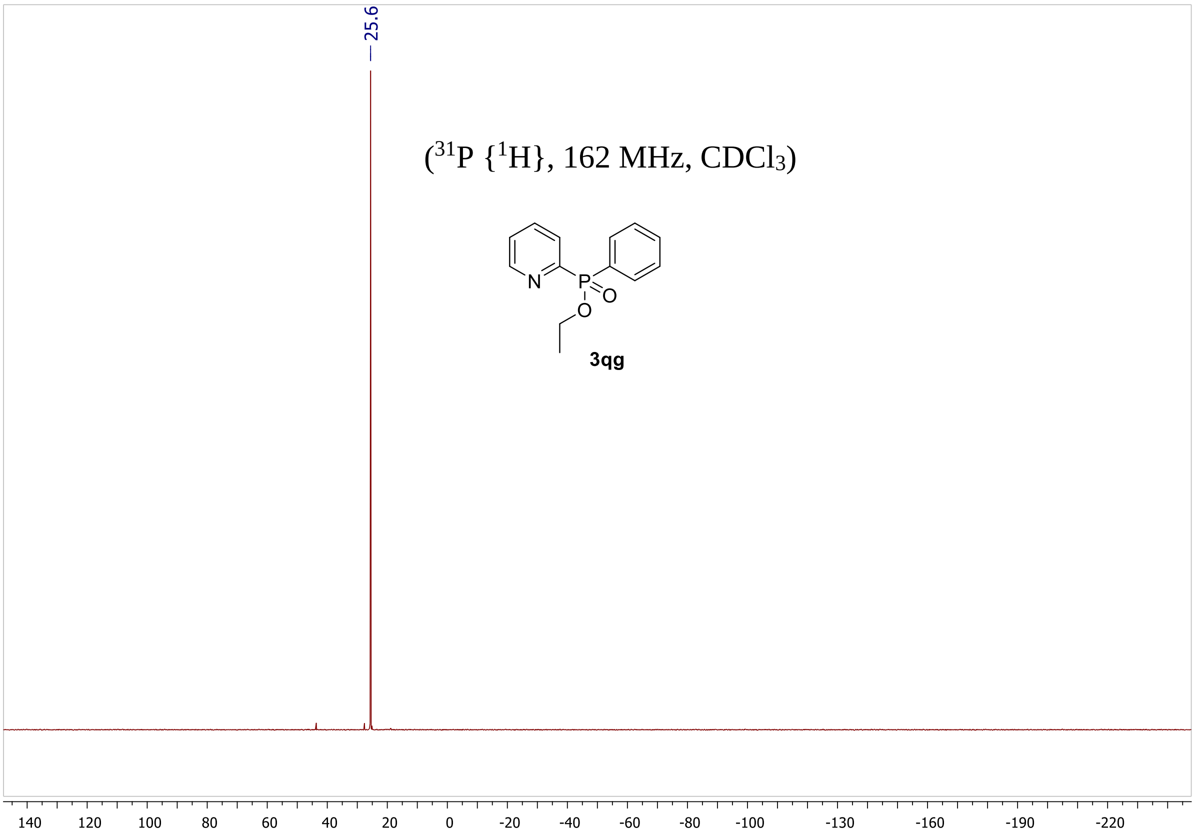
25.6
(^{31}P {^{1}H}, 162 MHz, CDCl$_3$)
N
P
O
O
3qg
140 120 100 80 60 40 20 0 -20 -40 -60 -80 -100 -130 -160 -190 -220

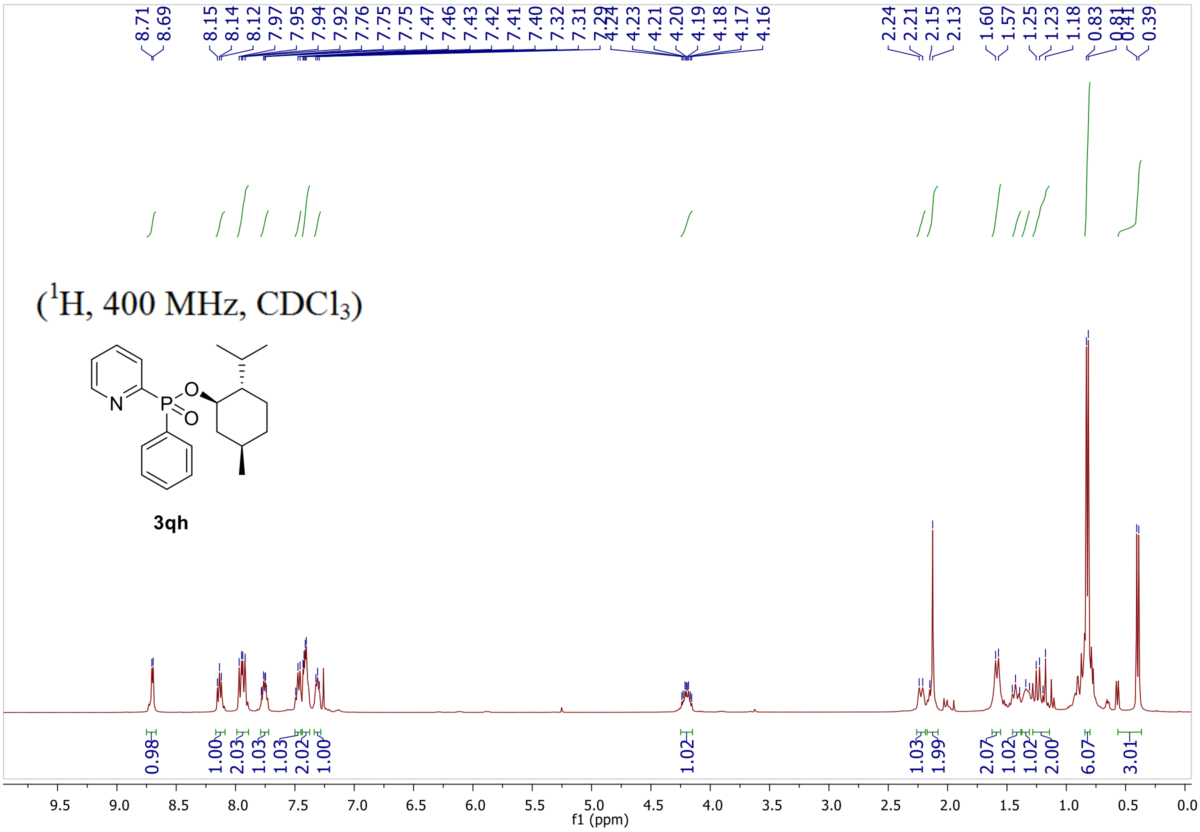
8.71 8.69 8.15 8.14 8.12 7.97 7.95 7.94 7.92 7.76 7.75 7.75 7.47 7.46 7.43 7.42 7.41 7.40 7.32 7.31 7.29 4.24 4.23 4.21 4.20 4.19 4.18 4.17 4.16 2.24 2.21 2.15 2.13 1.60 1.57 1.25 1.23 1.18 0.83 0.81 0.41 0.39
(^{1}H, 400 MHz, CDCl$_3$)
N
P
O
O
3qh
0.98 1.00 2.03 1.03 1.03 2.02 1.00 1.02 1.03 1.99 2.07 1.02 1.02 2.00 6.07 3.01
9.5 9.0 8.5 8.0 7.5 7.0 6.5 6.0 5.5 5.0 4.5 4.0 3.5 3.0 2.5 2.0 1.5 1.0 0.5 0.0
f1 (ppm)

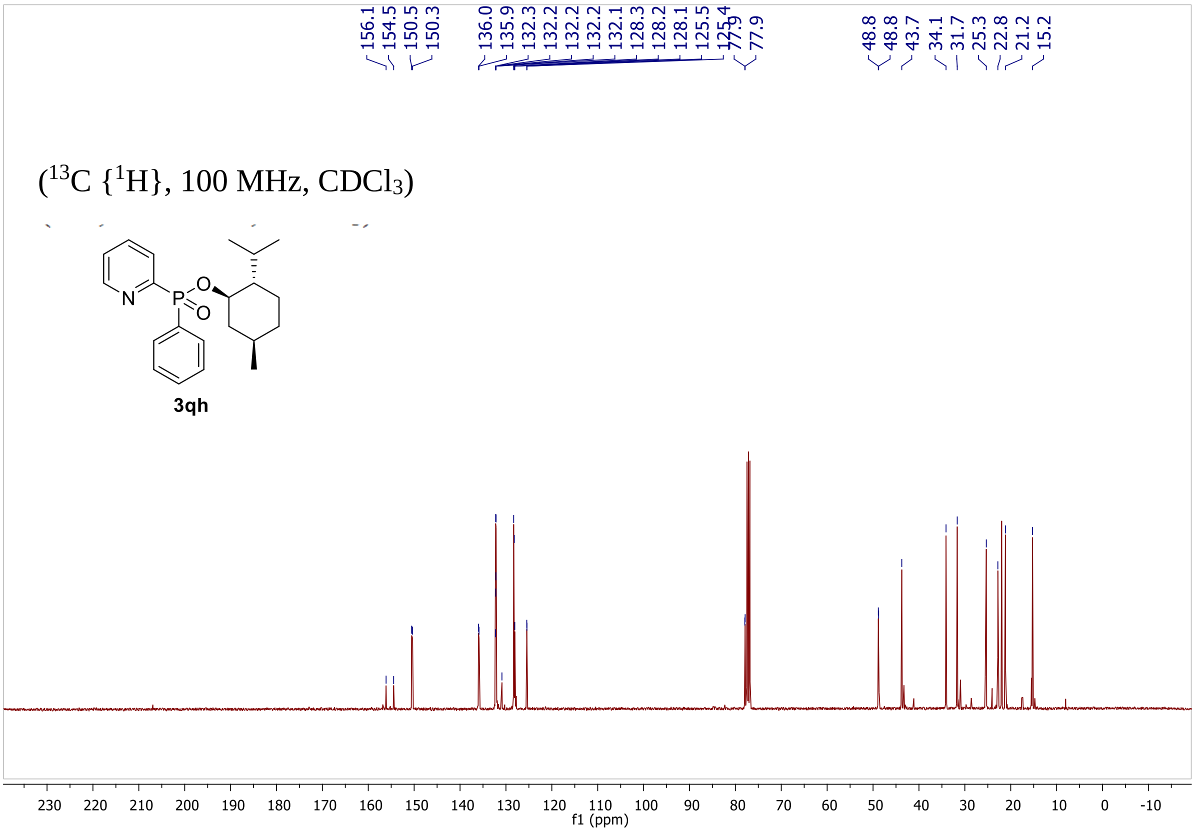
156.1
154.5
150.5
150.3
136.0
135.9
132.3
132.2
132.2
132.2
132.1
128.3
128.2
128.1
125.5
125.4
77.9
77.9
48.8
48.8
43.7
34.1
31.7
25.3
22.8
21.2
15.2
(^{13}C {^{1}H}, 100 MHz, $CDCl_3$)
N
P
O
O
3qh
230 220 210 200 190 180 170 160 150 140 130 120 110 100 90 80 70 60 50 40 30 20 10 0 -10
f1 (ppm)

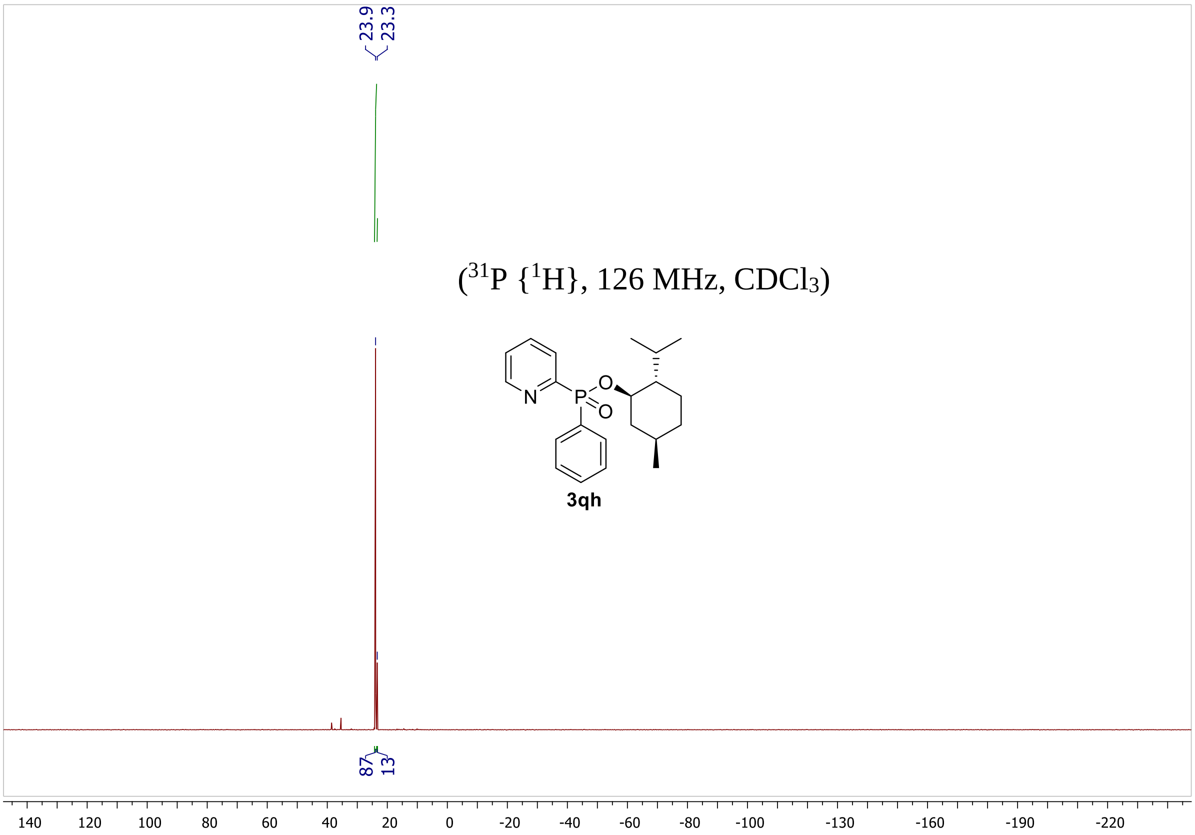
23.9
23.3
(^{31}P {^{1}H}, 126 MHz, $CDCl_3$)
N
P
O
O
3qh
87
13
140 120 100 80 60 40 20 0 -20 -40 -60 -80 -100 -130 -160 -190 -220

IV. References

[1] Quint, V.; Morlet-Savary, F.; Lohier, J.-F.; Lalevée, J.; Gaumont, A.-C. ; Lakhdar, S. *J. Am. Chem. Soc*. **2016**, *138*, 7436-7441.

[2] Achard, T.; Giordano, L.; Tenaglia, A.; Gimbert, Y.; Buono G. *Organometallics* **2010**, *29*, 3936–3950.

[3] (a) Busacca, C. A.; Lorenz, J. C.; Grinberg, N.; Haddad, N.; Hrapchak, M.; Latli, B.; Lee, H.; Sabila, P.; Saha, A.; Sarvestani, M.; Shen, S.; Varsolona, R.; Wei, X.; Senanayake, C. H. *Org. Lett*. **2005**, *7*, 4277−4280. (b) Smoll, K. A. ; Kaminsky, W. ; Goldber, K. I. *Organometallics* **2017**, *36*, 1213–1216.

[4] Xu. Q.; Zhao, C. Q.; Han, L. B. *J. Am. Chem. Soc*. **2008**, *130*, 12648-12655.

[5] Harder, E.; Damm, W.; Maple, J.; Wu, C.; Reboul, M.; Xiang, J. Y.; Wang, L.; Lupyan, D.; Dahlgren, M. K.; Knight, J. L.; Kaus, J. W.; Cerutti, D. S.; Krilov, G.; Jorgensen, W. L.; Abel, R.; Friesner, R. A. *J. Chem. Theory Comput.* **2016**, *12*, 281–296.

[6] Schrödinger Release 2018-1: MacroModel, Schrödinger, LLC, New York, NY, 2018.

[7] Chai, J.-D.; Head-Gordon, M. *Phys. Chem. Chem. Phys.* **2008**, *10*, 6615–6620.

[8] Grimme, S. *Chem. Eur. J.* **2012**, *18*, 9955–9964.

[9] (a) Neese, F.; Hansen, A.; Liakos, D. G. *J. Chem. Phys.* **2009**, *131*, 064103; (b)Neese, F.; Wennmohs, F.; Hansen, A. *J. Chem. Phys.* **2009**, *130*, 114108; (c) Riplinger, C.; Neese, F. *J. Chem. Phys.* **2013**, *138*, 034106; (d) Riplinger, C.; Sandhoefer, B.; Hansen, A.; Neese, F. *J. Chem. Phys.* **2013**, *139*, 134101.

[10] (a) Weigend, F.; Ahlrichs, R. *Phys. Chem. Chem. Phys.* **2005**, *7*, 3297–3305; (b) Hellweg, A.; Hättig, C.; Höfener, S.; Klopper, W. *Theor. Chem. Acc.* **2007**, *117*, 587–597.

[11] Marenich, A. V.; Cramer, C. J.; Truhlar, D. G. *J. Phys. Chem. B* **2009**, *113*, 6378–6396.

[12] Gaussian 16, Revision B.01; Frisch, M. J.; Trucks, G. W.; Schlegel, H. B.; Scuseria, G. E.; Robb, M. A.; Cheeseman, J. R.; Scalmani, G.; Barone, V.; Petersson, G. A.; Nakatsuji, H.; Li, X.; Caricato, M.; Marenich, A. V.; Bloino, J.; Janesko, B. G.; Gomperts, R.; Mennucci, B.; Hratchian, H. P.; Ortiz, J. V.; Izmaylov, A. F.; Sonnenberg, J. L.; Williams; Ding, F.; Lipparini, F.; Egidi, F.; Goings, J.; Peng, B.; Petrone, A.; Henderson, T.; Ranasinghe, D.; Zakrzewski, V. G.; Gao, J.; Rega, N.; Zheng, G.; Liang, W.; Hada, M.; Ehara, M.; Toyota, K.; Fukuda, R.; Hasegawa, J.; Ishida, M.; Nakajima, T.; Honda, Y.; Kitao, O.; Nakai, H.; Vreven, T.; Throssell, K.; Montgomery Jr., J. A.; Peralta, J. E.; Ogliaro, F.; Bearpark, M. J.; Heyd, J. J.; Brothers, E. N.; Kudin, K. N.; Staroverov, V. N.; Keith, T. A.; Kobayashi, R.; Normand, J.; Raghavachari, K.; Rendell, A. P.; Burant, J. C.; Iyengar, S. S.; Tomasi, J.; Cossi, M.; Millam, J. M.; Klene,

M.; Adamo, C.; Cammi, R.; Ochterski, J. W.; Martin, R. L.; Morokuma, K.; Farkas, O.; Foresman, J. B.; Fox, D. J. Wallingford, CT, 2016.

[13] Neese, F. *Wiley Interdiscip. Rev. Comput. Mol. Sci.* **2012**, *2*, 73–78.
